# Supplementary material for: Implication of Irisin in Different Types of Cancer: A Systematic Review and Meta-Analysis
Source: Int J Mol Sci. 2022 Sep 1;23(17):9971. doi: 10.3390/ijms23179971 (PMC9456043; doi:10.3390/ijms23179971)
Supplement: Supplementary file 1 [file ijms-23-09971-s001.zip › Supplement S1_R1.pdf]

Supplement S1

Implication of irisin in different types of cancer: A Systematic Review and Meta-analysis.

Maria Vliora[1,2], Eleni Nintou[1], Eleni Karligiotou[1], Leonidas Ioannou[1], Elisabetta Grillo[2], Stefania Mitola[2], Andreas Flouris[1]

1.FAME Laboratory, Department of Physical Education and Sport Science, University of Thessaly, Trikala, Greece

2. Department of Molecular and Translational Medicine, University of Brescia, Brescia, Italy

1. **Materials and Methods**

1.1. Searching algorithm used in PubMed

The following algorithm was applied to PubMed, EMBASE, and Cochrane library modified accordingly for each database:

Search ((((irisin[Title/Abstract]) OR fndc5/irisin[Title/Abstract]) OR fibronectin type III domain containing 5/irisin[Title/Abstract])) AND ((((((((((cancer[Title/Abstract]) OR tumor[Title/Abstract]) OR tumour[Title/Abstract]) OR tumor growth[Title/Abstract]) OR tumour growth[Title/Abstract]) OR tumor progression[Title/Abstract]) OR tumour progression[Title/Abstract]) OR cancer cachexia[Title/Abstract]) OR metastasis[Title/Abstract]) OR malignant growth[Title/Abstract]) OR malignancy[Title/Abstract]) Sort by: Best Match

1.2. Participants in *in vivo* studies

Table S1. Number of participants that were assessed in the systematic review

Author and year	Healthy	Diseased	Total
Altay et al 2016 [1]	12	48	60
Altay et al 2018 [2]	25	23	48
Aslan et al 2020 [3]	30	50	80
Castro G et al, 2020 [4]	-	94	94
Cheng et al., 2020 [5]	30	30	60
Coletta et al., 2021 [6]	33	-	33
Cebulski K et al 2022[7]	61	541	602
Gaggini et al 2017 [8]	18	18	36
Esawy et al 2020 [9]	75	75	150
Kim H. et al., 2019[10]	-	138	138
Provatopoulou X et al [11]	51	101	152
Pazgan-Simon et al [12]	20	69	89
Panagiotou et al 2016 [13]	80	16	96
Pinkowska A. et al 2022 [14]	14	140	154
Sadim M.et al., 2017 [15]	-	393	393
Shi G. et al. [16]	20	20	40
Taken K. et al 2021 [17]	30	90	120
Temur et al. 2021[18]	20	60	80
Wozniak S. et al 2022[19]	26	222	248
Zhu et al. 2018 [20]	40	42	82
Zhang J et al. 2019 [21]	102	117	219
Zhang Z. et. al, 2018 [22]	-	148	148
Zybek-Kocik et al.[23]	12	23	55
Shahidi et al.[24]	29	22	51
Ugur et al 2019 [25]	20	140	160
Nowinska et al [26]	240	729	969
**Total**	**988**	**3349**	**4357**

2. Results

2.1. Risk of Bias Assessment

Table S2. Office for Human and Animal Studies (OHAT) tool. Risk of bias Assessment.

	Selection Bias			Confounding bias	Performance bias		Attrition/ Exclusion bias	Detection Bias		Selective reporting bias	Other sources of bias	TOTAL SCORE
	Was administered dose or exposure level adequately randomized?	Was allocation to study groups adequately concealed?	Did selection of study participants result in appropriate comparison groups?	Did the study design or analysis account for important confounding and modifying variables?	Were experimental conditions identical across study groups?	Were the research personnel and human subjects blinded to the study group during the study?	Were outcome data complete without attrition or exclusion from analysis?	Can we be confident in the exposure characterization?	Can we be confident in the outcome assessment?	Were all measured outcomes reported?	Were there no other potential threats to internal validity (e.g., statistical methods were appropriate, and researchers adhered to the study protocol)?	
**CASE-CONTROL STUDIES**	(min = 0 – max = 21)											
Altay D. et. al, 2018			+	++			++	++	+	++	–	17
Aslan R. et al, 2020			++	++			++	++	+	++	++	20
Aydin S. et. al, 2016			+	++			++	–	–	++	–	14
Castro et al 2020			++	++			++	+	+	++	––	16
Cheng et al 2020			+	++			++	++	+	++	NR	17
Cebulski K et al 2022			++	+			++	++	++	++	++	20
Esawy et al 2020			++	++			++	++	++	++	++	21

Gaggini M et. al, 2017			++	++			++	–	++	++	– –	16
Nowinska K et al 2019			++	++			++	++	++	++	++	21
Pazgan-Simon et al 2020			+	++			++	–	– –	++	– –	12
Pinkowska A. et al 2022			++	+			++	++	++	++	++	20
Provatopoulou X. et. al, 2015			+	++			++	++	++	++	++	20
Shahidi S et al 2020			++	++			++	++	–	++	–	17
Shi G. et. al, 2017			++	++			++	++	+	++	NR	18
Taken K. et al 2021			++	+			++	++	++	++	++	20
Temur et al. 2021			++	+			++	++	++	++	+	19
Ugur et al, 2009			+	++			++	++	+	++	NR	17
Zhu H et. al, 2018			+	++			++	++	–	++	++	18
Zhang J. et al, 2019			++	++			++	++	++	++	+	20
Zybeck-Kocik et al, 2018			+	++			++	++	–	++	–	16
Wozniak S. et al 2022			++	++			++	++	++	++	++	21
Kuloglu et al_2019			++	++			++	++	++	++	++	21
Kuloglu et. al, 2016			++	++			++	++	++	++	++	21
**CROSS SECTIONAL STUDIES**	**(min = 0 – max = 24)**											
Sadim_et_al-2017			++	++		++	++	++	+	++	++	23
	**(min = 0 – max = 27)**											
Altay D. et al, 2016	++	++			++	NR	++	++	++	++	++	25
**HRCT**	**(min = 0 – max = 27)**											
Coletta et al 2021	++	+		++		NR	++	++	++	++	++	24
panagiotou et al 2016	++	+		++		NR	++	++	NR	++	++	23

COHORT STUDIES	(min = 0 – max = 21)							
Zhang Z. et. al, 2018	++	++	++	++	+	++	–	18
Kim et al, 2019	++	++	++	NR	++	++	+	18
panagiotou et al 2016	++	++	++	NR	++	++	++	19

Table S3. Office for Human and Animal Studies (OHAT) modified tool for *in vitro* studies. Risk of bias Assessment.

	Selection Bias			Confounding bias	Performance bias	Attrition/ Exclusion bias	Detection Bias		Selective reporting bias	Other sources of bias	TOTAL SCORE
	Were appropriate negative and positive controls applied in the study design?	Were the chosen cell lines appropriate and from reliable source?	Is there sufficient number of biological/ technical replicates?	Did the study design or analysis account for important confounding and modifying variables?	Were the research personnel and human subjects blinded to the study group during the study?	Were outcome data complete without attrition or exclusion from analysis?	Can we be confident in the exposure/treatment characterization?	Can we be confident in the outcome assessment?	Were all measured outcomes reported?	Were there no other potential threats to internal validity (e.g., statistical methods were appropriate, and researchers adhered to the study protocol)?	
***IN VITRO* STUDIES**					(min = 0 – max = 30)						
Fan G. et al, 2020	++	++	+	++	++	++	++	++	++	++	29
Gannon et al, 2015	++	++	++	++	++	++	++	++	++	++	30
Huang C.W. et al, 2020	++	++	++	++	++	++	++	++	++	++	30
Kong G. et. Al, 2017	++	++	+	++	++	++	++	++	++	++	29
Liu J et al 2019	+	++	++	++	++	++	++	++	++	++	29
Liu J. et. al, 2018	+	++	+	++	++	++	++	++	++	++	28
Moon et. al, 2014	++	++	++	++	++	++	++	++	++	++	30
Nowinska K. et al, 2019	++	++	++	++	++	++	++	++	++	++	30
Sadr A.S. et al 2022[27]	++	++	++	++	++	++	++	−	++	−	26
Shi G. et. al, 2017	++	−	++	++	++	++	++	++	++	++	28
Tekin S. et al, 2015	++	−	+	++	++	++	++	+	++	++	26
Zhang D et al., 2019	++	++	++	++	++	++	++	++	++	++	30
Shao L. et. al, 2017	++	++	+	++	++	++	++	++	++	++	29

2.2. Data extraction

The data that were extracted from the eligible publications have been divided in three tables: in vivo studies that have assessed irisin protein levels in blood samples, in vivo studies that assessed irisin protein levels on tissues, and in vitro studies.

Table S4. Data extraction table. *In vivo* studies that assessed serum irisin levels in blood samples.

Author and year	Type of cancer	Assay/ Antibody	General Conclusion
Altay et al 2016 [1]	gastric cancer	ELISA kit/ Sunred Biological Technology Co., Shanghai	Factors released from gastric tumor tissue activate a number of signaling pathways, stimulating FNDC5 and its coactivator PGC1-a in white and brown adipose tissue, and thus an increase in irisin in the circulation. No indication of how irisin is increased in the circulation though.
Shahidi et al 2020 [24]	gastric cancer	ELISA kit/ Bioassay Technology Laboratory, China	Decreased irisin serum level could be a prognostic factor for gastric cancers
Temur et al. 2021[18]	Gastric and colorectal cancer	ELISA kit /(Mybiosource, USA)	Reduced irisin level might lead to absence a potential protective mechanism of irisin against cancer. Importantly, irisin could be powerful potential diagnostic biomarker which would be use for early detection of gastric and colorectal cancers.
Altay et al 2018 [2]	renal cancer	ELISA kit/ Sunred Biological Technology Co., Shanghai	Increased irisin serum protein expression in renal cancer group. Increased irisin in the circulation leads to weight loss of mice with experimentally induced gastric cancer.
Aslan et al 2020 [3]	prostate cancer	ELISA kit/YL biont biotech Co. Shanghai	Decreased serum protein concentration of irisin in prostate cancer patients. Irisin can potentially be used as a biomarker for prostate cancer.
Esawy et al 2020 [9]	bladder cancer	ELISA kits/ BioVendor Laboratory Medicine, Brno, Czech Republic	Decreased serum irisin levels in cancer patients. Serum irisin can be a good biomarker for prognosis and diagnosis of bladder cancer
Taken K. et al 2021 [17]	Bladder cancer	ELISA kit/ Adipogen AG, Liestal, Switzerland	Serum irisin levels were significantly decreased in bladder cancer patients compared to healthy individuals. It can distinguish high-grade and stage tumors at presentation. Although detailed and long-term studies are required to elucidate the molecular and mechanistic basis of its use as well as biomarker dynamics in bladder cancer patients, serum irisin level seems to be

			a promising biomarker to detect bladder cancer.
Cheng et al., 2020 [5]	osteosarcoma	ELISA kit/ Adipogen AG, Liestal, Switzerland	Decreased serum protein concentration of irisin in osteosarcoma patients.Irisin/FNDC5 and miR-214-3p might be used for the treatment of osteosarcoma patients in the future.
Coletta et al., 2021 [6]	breast cancer	Luminex® Human Myokine Magnetic Bead Panel / HMYOMAG-56 K, Millipore Corporation, Billerica MA	Reduction of resting levels of irisin after moderate-intensity continuous-training. Exercise intensity does not impact the myokines levels in overweight postmenopausal women with increased risk for breast cancer.
Provatopoulou X et al [11]	breast cancer	ELISA kit/ AdipoGen International, Liestal, SW	Decreased serum levels of irisin breast cancer patients. Irisin is implicated in breast cancer development and can potentially serve as biomarker for the presence of the disease.
Sadim M.et al., 2017 [15]	breast cancer	Radio-immunoassay kit /Phoenix Pharmaceuticals, Burlingame,Calif)	Irisin/ FNDC-5 SNP, rs726344 was significantly associated with weight change at 18 months in univariate analysis.
Zhang Z. et. al, 2018 [22]	breast cancer to spinal metastasis	ELISA kit/ Aviscera Biosciences, Santa Clara, CA	The serum irisin was higher in patients without spinal metastasis. Higher serum irisin can be a protective factor of spinal metastasis in patients with breast cancer.
Kim H. et al., 2019[10]	Metastatic solid tumors	Luminex multiplex assay / Human Myokine magnetic bead panel kit, Merck Millipore	Irisin does not predict overall survival for patients with metastatic solid tumors.
Gaggini et al 2017 [8]	hepatocellular carcinoma	ELISA kit/ Adipogen AG, Liestal, Switzerland	No change in irisin protein level. Increased mRNA expression in HCC patients. Irisin could be used a s a future therapeutic agent for treatment of carcinogenesis.
Pazgan-Simon et al [12]	hepatocellular carcinoma	Elisa Kit / BioVendor-Laboratorni Medicina	Decreased serum levels of irisin in HCC patients. Lower irisin levels favor faster fibrosis progression and facilitate cancer progression. No clear effect of irisin on carcinogenesis
Shi G. et al. [16]	hepatocellular carcinoma	ELISA kit/ USCN Life Science, Wuhan, China	Serum irisin level unaltered in healthy and diseased participants. Irisin mRNA expression increased in patients, may have protective roles in liver cancer cells through activation of the PI3K/AKT pathway, which may induce liver cancer progression and decreased sensitivity to chemotherapy.
Zhang J et al. 2019 [21]	hepatocellular carcinoma	ELISA kit/ USCN Life Science, Wuhan, China	Decreased Irisin serum level in patients. Irisin may be a novel serum biomarker in the diagnosis of HCC and a predictor of complications after hepatectomy.
Pinkowska A. et al 2022	Laryngeal squamous cell cancer	Western blot/ polyclonal anti-irisin/FNDC5 antibody (1:200, code no. NBP2-	Higher irisin expression for LSCC compared to control tissues and association with tumor growth and lymph node metastasis. Potential role

		14024, Novus Biologicals)	of irisin as a biomarker in the diagnostic process of LSCC.
Panagiotou et al 2016 [13]	thyroid cancer	ELISA kit/ Phoenix Pharmaceuticals, Burlingame, CA	Decreased serum irisin levels in patients. subclinical or interventional changes of thyroid state do not affect the levels of irisin in humans
Zybek-Kocik et al.[23]	thyroid cancer	ELISA kit/ Phoenix Pharmaceuticals, Burlingame, CA	Irisin concentration changes are associated with prolonged hypothyroidism and might primarily be the result of prolonged myopathy.
Zhu et al. 2018 [20]	colorectal cancer	ELISA kit/ USCN Life Science, Wuhan, China	Individuals with high ATF3 and low irisin levels were more likely to have CRC. Irisin may represent diagnostic biomarkers for CRC patients together with ATF3.
Wozniak S. et al 2022[19]	Colorectal cancer	rabbit polyclonal anti-irisin/FNDC5 antibody (NBP2-14024; Novus Biologicals, Littleton, CO, USA)	There is a possibility that the situation in the gastrointestinal tract is similar to that of adipose tissue, in which the level of irisin expression is different in brown, white and beige tissues. Therefore, the final results in various types of cancer are also divergent. However, the data from our study may be useful for understanding the role of this peptide in CRC progression.
Castro G et al, 2020 [4]	Cancer associated cachexia	Myokine quantification kit/ HMYOMAG-56 K, Merck-Millipore, St. Charles, MO, USA	Different myokine content in skeletal muscle, plasma, and tumor from patients with cancer-associated cachexia may have a role in tumor evasion, inflammation, and tissue remodeling. These changes may be implicated in the decreased capacity for skeletal muscle regeneration, increased muscle breakdown and tumor aggressiveness associated with cachexia

Table S5. Data extraction table. *In vivo* studies that assessed irisin protein levels on various tissues.

Author and year	Type of cancer	Antibody	General Conclusion
Aydin et al 2016 [28]	Hepatocellular carcinoma, brain cancer, colon cancer, stomach cancer, esophageal cancer, pancreatic cancer	anti-irisin, Phoenix Pharmaceuticals, Inc., Burlingame, CA	Increased irisin immunoreactivity on tissues of gastrointestinal system cancers but not on liver cancer. Additional investigation required for the use of irisin treatment of cancer by induction of hyperthermia.
Kuloglu et al 2016 [29]	Breast cancer, ovarian cancer	Not reported	Increased immunoreactivity of irisin in breast and ovarian cancers. Accordingly, irisin may regulate the thermal activity inside breast and ovarian tumors

			and then prohibit the proliferation of cancer cells.
Cebulski K et al 2022[7]	Breast cancer	IHC/ polyclonal rabbit anti-irisin (code no. NBP2-14024; Novus Biologicals, Littleton, CO, USA)	Decrease in irisin expression levels in breast cancer. High irisin levels were associated with longer survival. Further studies are warranted to explain the mechanism of the effect of irisin on proliferation, migration, and EMT, and to test the possibility of using it as a target in potential therapy.
Kuloglu et al 2019 [30]	Renal cancers	anti-irisin, Phoenix Pharmaceuticals, Inc., Burlingame, CA	Decreased irisin expression was detected in chromophobe RCC, while strong immunostaining was detected in oncocytomas. Irisin immunoreactivity may be a useful test for differentiating benign lesions from renal cancer.
Ugur et al 2019 [25]	Thyroid cancer	anti-irisin, Phoenix Pharmaceuticals, Inc., Burlingame, CA	Irisin immunoreactivity can be used as a biomarker for differentiating oncocytic variants of thyroid carcinomas from other types of thyroid cancer. Irisin is involved in carcinogenesis in the thyroid gland.
Nowinska et al [26]	Lung cancer	anti-irisin/FNDC5; Novus Biologicals, Littleton, CO, USA	Irisin was expressed in NSCLC tumors. Significant difference in the levels of irisin expression in cancer cells of SCC compared to the AC subtype. Expression of irisin in stromal fibroblasts may be associated with an increased proliferation of cancer cells and may also be an independent prognostic factor for survival in patients with NSCLC.

Table S6. Data extraction table. *In vitro* studies.

Author and year	Type of cancer/ cell lines	Type of irisin	General conclusions
Fan G. et al. 2020 [31]	Lung cancer/ A549, H358, H1299, H1650 HBE and BEAS-2	Human recombinant irisin, non-modified, Sigma-Aldrich (St. Louis, MO, USA).	Lower irisin expression in cancer cell lines. Irisin inhibits cell proliferation in cancer cell lines. Irisin a promising therapeutic agent for lung cancer.
Nowinska K et al 2019 [26]	Lung cancer/ NCI-H1703 (SCC) and NCI-H522 (AC)	polyclonal rabbit anti-irisin/FNDC5, (Novus Biologicals, Littleton, CO, USA)	Irisin expression in stromal fibroblasts may influence cancer cell proliferation and may be a prognostic factor for survival in Lung cancer.
Shao L. et. al, 2017 [32]	Lung cancer/ A549 cells and NCIH446 cells	Not reported	Irisin inhibits EMT and reduces the invasion of lung cancer cells via mediating the PI3K/AKT/Snail signaling pathway. The migration and invasive ability of lung cancer cells may be controlled by irisin, by inhibiting the PI3K/AKT/Snail signaling pathway and EMT.
Gannon_et_al-2015 [33]	Breast cancer/MCF-10a, MCF-7, MDA-MB-231	Human recombinant non-modified irisin (INM), Cayman Chemical (Ann Arbor, MI)	Irisin may allow for reduced doses of common antineoplastic agents (Increased tumor sensitivity) thereby improving patient tolerance and prognosis. Irisin may be a future therapeutic agent.
Huang C.W. et al. 2020 [34]	Glioblastoma/ U-87 MG, T98G, LN-18 and 3T3-L1 cell line	radioactive 68Ga-DOTA-irisin (no further info)	Consistent irisin administration can effectively inhibit cell proliferation, reduce protease secretion, impede cell invasion and restrict tumor growth. Irisin may be useful as a prognostic biomarker.
Kong G. et. al 2017 [35]	Osteosarcoma/ U2OS and MG-63	Irisin (modified) Phoenix Pharmaceuticals (Burlingame, CA, USA)	Irisin suppressed the migration and invasion of osteosarcoma Cells reversed the EMT induced by IL-6 in osteosarcoma cells. It has an inhibitory role in IL-6-induced EMT modulated via the STAT3/Snail pathway. Irisin is a promising agent in osteosarcoma treatment.
Liu J et al 2019 [36]	Pancreatic cancer/ MIA PaCa-2, BxPC-3, and H9c2	Non modified irisin by Sangon Biotech (Shanghai, China)	Irisin increases the chemosensitivity of PC cells to DOX or GEM and enhance DOX-induced apoptosis in PC cancer cells through upregulation of PARP and caspase-3 and downregulation Bcl-2, BCL-xL, and PI3K/AKT/NF-kB signaling pathway. irisin could be used as an adjunctive agent combined

			with chemotherapy for the treating PC cells.
Liu J. et. al, 2018 [37]	Pancreatic cancer/ MIA PaCa-2 and Panc03.27	Anti-Irisin (Human, Rat, Mouse, Canine specific) antibody, Phoenix Pharmaceuticals (CA, USA).	Irisin inhibits migration and invasion of PC cells via inhibition of epithelial-to-mesenchymal transition pathway. Irisin activates the AMPK-mTOR signaling pathway, which may play a critical role in irisin-induced inhibition of pancreatic cancer cell growth. Irisin may be employed as a therapeutic candidate for the treatment of pancreatic cancer in clinical practices.
Zhang D et al., 2019 [38]	Pancreatic cancer/ PANC-1 and BxPC-3	Irisin human recombinant (modified), (Cayman Chemicals, MI, USA)	Irisin could inhibit cell proliferation and induce the apoptosis of pancreatic cancer cells. In addition, irisin was able to downregulate the activation of the PI3K/AKT signaling pathway in pancreatic cancer cells. Irisin could be used as a therapeutic agent for pancreatic cancer.
Moon et. al, 2014 [39]	Endometrial, colon, thyroid and esophageal cancer/ KLE and RL95-2, HT29, MCA38, SW579, BHP7, OE13 and OE33	Human recombinant irisin made by Aviscera Bioscience (Santa Clara, CA) by Phoenix Pharmaceuticals (Burlingame, CA).	Irisin, in physiological and high physiological/ pharmacological concentrations, has no in vitro effect on cell proliferation and malignant potential of obesity-related cancer cell lines.
Shi G. et. al, 2017 [16]	Hepatocellular carcinoma/ SMMC7721 cells	human recombinant modified (glycosylated) irisin (IM) from PlexBio (San Francisco, CA, USA) and human recombinant nonmodified irisin (INM; Cayman Chemical, Ann Arbor, MI, USA)	Increased irisin levels may have protective roles in liver cancer cells through partial activation of the PI3K/AKT pathway, which may facilitate liver cancer progression and decrease the sensitivity to chemotherapy.
Tekin S. et al, 2015 [40]	Prostate cancer/ LNCaP, DU-145, PC3	Irisin (modified) Phoenix Pharmaceuticals (Burlingame, CA, USA)	Our study results exerted that treatment with the physiological and pharmacological concentrations of irisin decreased cell viability in androgen receptor positive and negative prostate cancer cell lines in a dose dependent manner. Cytotoxic effects of irisin emerge via an androgen receptor independent mechanism.
Sadr A.S. et al 2022 [27]	Prostate cancer cell lines LNCaP and DU-145	Not reported	Irisin can inhibit tumor development and induce apoptosis by inhibition of EMT through various signaling pathways. Some of the results are contradicting to previous studies which may be due to

	experimental methods used or tissue or cell line properties. It is possible using irisin as a new attractive and potential therapeutic target drug and as prognostic and diagnostic biomarker to treat prostate cancers in the future.

3. References

1. Us Altay D, Keha EE, Ozer Yaman S, Ince I, Alver A, Erdogan B, et al. Investigation of the expression of irisin and some cachectic factors in mice with experimentally induced gastric cancer. Qjm. 2016;109(12):785-90. Epub 2016/06/04. doi: 10.1093/qjmed/hcw074. PubMed PMID: 27256459.
2. Altay DU, Keha EE, Karagüzel E, Menteşe A, Yaman SO, Alver A. The Diagnostic Value of FNDC5/Irisin in Renal Cell Cancer. Int Braz J Urol. 2018;44(4):734-9. Epub 2018/03/10. doi: 10.1590/s1677-5538.ibju.2017.0404. PubMed PMID: 29522296; PubMed Central PMCID: PMCPMC6092672.
3. Aslan R, Alp HH, Eryılmaz R, Huyut Z, Sevim M, Araz Ş, et al. Can the Irisin be a Biomarker for Prostate Cancer? A Case Control Study. Asian Pac J Cancer Prev. 2020;21(2):505-9. Epub 2020/02/28. doi: 10.31557/apjcp.2020.21.2.505. PubMed PMID: 32102531; PubMed Central PMCID: PMCPMC7332134.
4. de Castro GS, Correia-Lima J, Simoes E, Orsso CE, Xiao J, Gama LR, et al. Myokines in treatment-naïve patients with cancer-associated cachexia. Clinical nutrition (Edinburgh, Scotland). 2021;40(4):2443-55. Epub 2020/11/17. doi: 10.1016/j.clnu.2020.10.050. PubMed PMID: 33190987.
5. Cheng G, Xu D, Chu K, Cao Z, Sun X, Yang Y. The Effects of MiR-214-3p and Irisin/FNDC5 on the Biological Behavior of Osteosarcoma Cells. Cancer biotherapy & radiopharmaceuticals. 2020;35(2):92-100. Epub 2020/02/20. doi: 10.1089/cbr.2019.2933. PubMed PMID: 32073886.
6. Coletta AM, Agha NH, Baker FL, Niemiro GM, Mylabathula PL, Brewster AM, et al. The impact of high-intensity interval exercise training on NK-cell function and circulating myokines for breast cancer prevention among women at high risk for breast cancer. Breast Cancer Res Treat. 2021;187(2):407-16. Epub 2021/02/09. doi: 10.1007/s10549-021-06111-z. PubMed PMID: 33555464; PubMed Central PMCID: PMCPMC8189992.
7. Cebulski K, Nowinska K, Jablonska K, Romanowicz H, Smolarz B, Dziegiel P, et al. Expression of Irisin/FNDC5 in Breast Cancer. International Journal of Molecular Sciences. 2022;23(7):3530. doi: https://dx.doi.org/10.3390/ijms23073530.
8. Gaggini M, Cabiati M, Del Turco S, Navarra T, De Simone P, Filipponi F, et al. Increased FNDC5/Irisin expression in human hepatocellular carcinoma. Peptides. 2017;88:62-6. Epub 2016/12/26. doi: 10.1016/j.peptides.2016.12.014. PubMed PMID: 28012856.
9. Esawy MM, Abdel-Samd KM. The diagnostic and prognostic roles of serum irisin in bladder cancer. Curr Probl Cancer. 2020;44(4):100529. Epub 2020/03/07. doi: 10.1016/j.currproblcancer.2019.100529. PubMed PMID: 32139156.
10. Kim SH, Kim JW, Hwang IG, Jang JS, Hong S, Kim TY, et al. Serum biomarkers for predicting overall survival and early mortality in older patients with metastatic solid tumors. J Geriatr Oncol. 2019;10(5):749-56. Epub 2019/04/07. doi: 10.1016/j.jgo.2019.03.015. PubMed PMID: 30952517.

11. Provatopoulou X, Georgiou GP, Kalogera E, Kalles V, Matiatou MA, Papapanagiotou I, et al. Serum irisin levels are lower in patients with breast cancer: association with disease diagnosis and tumor characteristics. BMC Cancer. 2015;15:898. Epub 2015/11/13. doi: 10.1186/s12885-015-1898-1. PubMed PMID: 26560078; PubMed Central PMCID: PMCPMC4642638.
12. Pazgan-Simon M, Zuwala-Jagiello J, Menzyk T, Bator M, Derra A, Lekstan A, et al. Serum betatrophin and irisin levels in hepatocellular carcinoma. J Physiol Pharmacol. 2020;71(1). Epub 2020/06/20. doi: 10.26402/jpp.2020.1.11. PubMed PMID: 32554846.
13. Panagiotou G, Pazaitou-Panayiotou K, Paschou SA, Komninou D, Kalogeris N, Vryonidou A, et al. Changes in Thyroid Hormone Levels Within the Normal and/or Subclinical Hyper- or Hypothyroid Range Do Not Affect Circulating Irisin Levels in Humans. Thyroid : official journal of the American Thyroid Association. 2016;26(8):1039-45. Epub 2016/06/09. doi: 10.1089/thy.2016.0098. PubMed PMID: 27267080.
14. Pinkowska A, Podhorska-Okolow M, Dziegiel P, Nowinska K. The role of irisin in cancer disease. Cells. 2021;10(6):1479. doi: https://dx.doi.org/10.3390/cells10061479.
15. Sadim M, Xu Y, Selig K, Paulus J, Uthe R, Agarwl S, et al. A prospective evaluation of clinical and genetic predictors of weight changes in breast cancer survivors. Cancer. 2017;123(13):2413-21. Epub 2017/02/15. doi: 10.1002/cncr.30628. PubMed PMID: 28195643.
16. Shi G, Tang N, Qiu J, Zhang D, Huang F, Cheng Y, et al. Irisin stimulates cell proliferation and invasion by targeting the PI3K/AKT pathway in human hepatocellular carcinoma. Biochem Biophys Res Commun. 2017;493(1):585-91. Epub 2017/09/05. doi: 10.1016/j.bbrc.2017.08.148. PubMed PMID: 28867187.
17. Taken K, Aslan R, Eryilmaz R, Alp HH, Huyut Z, Donmez MI. Serum irisin is a novel biomarker for bladder cancer detection. International Urology and Nephrology. 2022;54(1):55-61. doi: https://dx.doi.org/10.1007/s11255-021-03074-4.
18. Abd Temur A, Aqeel Rashid F. Irisin and Carcinoembryonic Antigen (CEA) as Potential Diagnostic Biomarkers in Gastric and Colorectal Cancers. Reports of biochemistry & molecular biology. 2021;10(3):488-94. Epub 2022/01/05. doi: 10.52547/rbmb.10.3.488. PubMed PMID: 34981027; PubMed Central PMCID: PMCPMC8718786.
19. Wozniak S, Nowinska K, Chabowski M, Dziegiel P. Significance of Irisin (FNDC5) Expression in Colorectal Cancer. In Vivo. 2022;36(1):180-8. doi: https://dx.doi.org/10.21873/invivo.12689.
20. Zhu H, Liu M, Zhang N, Pan H, Lin G, Li N, et al. Serum and Adipose Tissue mRNA Levels of ATF3 and FNDC5/Irisin in Colorectal Cancer Patients With or Without Obesity. Front Physiol. 2018;9:1125. Epub 2018/09/25. doi: 10.3389/fphys.2018.01125. PubMed PMID: 30246803; PubMed Central PMCID: PMCPMC6140752.
21. Zhang J, Ke M, Ren Y, Bi J, Du Z, Zhang M, et al. Serum Irisin Predicts Posthepatectomy Complications in Patients with Hepatocellular Carcinoma. Dis Markers. 2019;2019:9850191. Epub 2020/01/25. doi: 10.1155/2019/9850191. PubMed PMID: 31976024; PubMed Central PMCID: PMCPMC6955133.
22. Zhang ZP, Zhang XF, Li H, Liu TJ, Zhao QP, Huang LH, et al. Serum irisin associates with breast cancer to spinal metastasis. Medicine. 2018;97(17):e0524. Epub 2018/04/29. doi: 10.1097/md.0000000000010524. PubMed PMID: 29703023; PubMed Central PMCID: PMCPMC5944558 declare that this manuscript has not been submitted or is not simultaneously being submitted elsewhere, and that no portion of the data has been or will be published in proceedings or transactions of meetings or symposium volumes. The authors report no conflicts of interest.
23. Zybek-Kocik A, Sawicka-Gutaj N, Szczepanek-Parulska E, Andrusiewicz M, Waligórska-Stachura J, Białas P, et al. The association between irisin and muscle metabolism

in different thyroid disorders. Clin Endocrinol (Oxf). 2018;88(3):460-7. Epub 2017/12/03. doi: 10.1111/cen.13527. PubMed PMID: 29197093.
24. Shahidi S, Hejazi J, Moghimi M, Borji S, Zabihian S, Fathi M. Circulating Irisin Levels and Redox Status Markers in Patients with Gastric Cancer: A Case-Control Study. Asian Pac J Cancer Prev. 2020;21(10):2847-51. Epub 2020/10/29. doi: 10.31557/apjcp.2020.21.10.2847. PubMed PMID: 33112539; PubMed Central PMCID: PMCPMC7798161.
25. Ugur K, Aydin S, Kuloglu T, Artas G, Kocdor MA, Sahin İ, et al. Comparison of irisin hormone expression between thyroid cancer tissues and oncocytic variant cells. Cancer Manag Res. 2019;11:2595-603. Epub 2019/05/23. doi: 10.2147/cmar.s201979. PubMed PMID: 31114326; PubMed Central PMCID: PMCPMC6497896.
26. Nowinska K, Jablonska K, Pawelczyk K, Piotrowska A, Partynska A, Gomulkiewicz A, et al. Expression of Irisin/FNDC5 in Cancer Cells and Stromal Fibroblasts of Non-small Cell Lung Cancer. Cancers (Basel). 2019;11(10). Epub 2019/10/17. doi: 10.3390/cancers11101538. PubMed PMID: 31614634; PubMed Central PMCID: PMCPMC6826442.
27. Saeedi Sadr A, Ehteram H, Seyed Hosseini E, Alizadeh Zarei M, Alizadeh Bafrani H, Haddad Kashani H. The Effect of Irisin on Proliferation, Apoptosis, and Expression of Metastasis Markers in Prostate Cancer Cell Lines. Oncology and Therapy. 2022. doi: https://dx.doi.org/10.1007/s40487-022-00194-4.
28. Aydin S, Kuloglu T, Ozercan MR, Albayrak S, Aydin S, Bakal U, et al. Irisin immunohistochemistry in gastrointestinal system cancers. Biotech Histochem. 2016;91(4):242-50. Epub 2016/03/11. doi: 10.3109/10520295.2015.1136988. PubMed PMID: 26963139.
29. Kuloglu T, Celik O, Aydin S, Hanifi Ozercan I, Acet M, Aydin Y, et al. Irisin immunostaining characteristics of breast and ovarian cancer cells. Cellular and molecular biology (Noisy-le-Grand, France). 2016;62(8):40-4. Epub 2016/08/23. PubMed PMID: 27545213.
30. Kuloğlu T, Artaş G, Yardim M, Sahin I, Aydin Y, Beyoğlu N, et al. Immunostaining characteristics of irisin in benign and malignant renal cancers. Biotech Histochem. 2019;94(6):435-41. Epub 2019/03/22. doi: 10.1080/10520295.2019.1586998. PubMed PMID: 30896263.
31. Fan GH, Zhu TY, Huang J. FNDC5 promotes paclitaxel sensitivity of non-small cell lung cancers via inhibiting MDR1. Cellular signalling. 2020;72:109665. Epub 2020/05/01. doi: 10.1016/j.cellsig.2020.109665. PubMed PMID: 32353410.
32. Shao L, Li H, Chen J, Song H, Zhang Y, Wu F, et al. Irisin suppresses the migration, proliferation, and invasion of lung cancer cells via inhibition of epithelial-to-mesenchymal transition. Biochem Biophys Res Commun. 2017;485(3):598-605. Epub 2016/12/18. doi: 10.1016/j.bbrc.2016.12.084. PubMed PMID: 27986567.
33. Gannon NP, Vaughan RA, Garcia-Smith R, Bisoffi M, Trujillo KA. Effects of the exercise-inducible myokine irisin on malignant and non-malignant breast epithelial cell behavior in vitro. Int J Cancer. 2015;136(4):E197-202. Epub 2014/08/16. doi: 10.1002/ijc.29142. PubMed PMID: 25124080.
34. Huang CW, Chang YH, Lee HH, Wu JY, Huang JX, Chung YH, et al. Irisin, an exercise myokine, potently suppresses tumor proliferation, invasion, and growth in glioma. FASEB journal : official publication of the Federation of American Societies for Experimental Biology. 2020;34(7):9678-93. Epub 2020/05/30. doi: 10.1096/fj.202000573RR. PubMed PMID: 32469121.
35. Kong G, Jiang Y, Sun X, Cao Z, Zhang G, Zhao Z, et al. Irisin reverses the IL-6 induced epithelial-mesenchymal transition in osteosarcoma cell migration and invasion through the STAT3/Snail signaling pathway. Oncol Rep. 2017;38(5):2647-56. Epub 2017/10/20. doi:

10.3892/or.2017.5973. PubMed PMID: 29048621; PubMed Central PMCID: PMCPMC5780017.
36. Liu J, Huang Y, Liu Y, Chen Y. Irisin Enhances Doxorubicin-Induced Cell Apoptosis in Pancreatic Cancer by Inhibiting the PI3K/AKT/NF-κB Pathway. Medical science monitor : international medical journal of experimental and clinical research. 2019;25:6085-96. Epub 2019/08/15. doi: 10.12659/msm.917625. PubMed PMID: 31412018; PubMed Central PMCID: PMCPMC6705179.
37. Liu J, Song N, Huang Y, Chen Y. Irisin inhibits pancreatic cancer cell growth via the AMPK-mTOR pathway. Sci Rep. 2018;8(1):15247. Epub 2018/10/17. doi: 10.1038/s41598-018-33229-w. PubMed PMID: 30323244; PubMed Central PMCID: PMCPMC6189061.
38. Zhang D, Zhang P, Li L, Tang N, Huang F, Kong X, et al. Irisin functions to inhibit malignant growth of human pancreatic cancer cells via downregulation of the PI3K/AKT signaling pathway. Onco Targets Ther. 2019;12:7243-9. Epub 2019/10/01. doi: 10.2147/ott.s214260. PubMed PMID: 31564907; PubMed Central PMCID: PMCPMC6732507.
39. Moon HS, Mantzoros CS. Regulation of cell proliferation and malignant potential by irisin in endometrial, colon, thyroid and esophageal cancer cell lines. Metabolism. 2014;63(2):188-93. Epub 2013/11/26. doi: 10.1016/j.metabol.2013.10.005. PubMed PMID: 24268368.
40. Suat Tekin YE, Suleyman Sandal , Bayram Yilmaz. Is Irisin an Anticarcinogenic Peptide? Medicine Science. Dec,2015;4(2):2172-80.
