# Supplementary material for: Implication of Irisin in Different Types of Cancer: A Systematic Review and Meta-Analysis
Source: Int J Mol Sci. 2022 Sep 1;23(17):9971. doi: 10.3390/ijms23179971 (PMC9456043; doi:10.3390/ijms23179971)
Supplement: Supplementary file 1 [file ijms-23-09971-s001.zip › Supplement S2_R1.pdf]

Supplement S2

Implication of irisin in different types of cancer: A Systematic Review and Meta-analysis.

Maria Vliora[1,2], Eleni Nintou[1], Eleni Karligiotou[1], Leonidas Ioannou[1], Elisabetta Grillo [2], Stefania Mitola[2], Andreas Flouris1

1.FAME Laboratory, Department of Physical Education and Sport Science, University of Thessaly, Trikala, Greece

2. Department of Molecular and Translational Medicine, University of Brescia, Brescia, Italy

Find below the list of the searched papers for this systematic review

1. Abd El-Mottaleb NA, Galal HM, El Maghraby KM, Gadallah AI. Serum irisin level in myocardial infarction patients with or without heart failure. Canadian Journal of Physiology and Pharmacology. 2019;97(10):932-8. doi: https://dx.doi.org/10.1139/cjpp-2018-0736.

2. Abd El-Mottaleb NA, Galal HM, El Maghraby KM, Gadallah AI. Serum irisin level in myocardial infarction patients with or without heart failure. Can J Physiol Pharmacol. 2019;97(10):932-8. Epub 2019/04/09. doi: 10.1139/cjpp-2018-0736. PubMed PMID: 30958967.

3. Abd Temur A, Aqeel Rashid F. Irisin and Carcinoembryonic Antigen (CEA) as Potential Diagnostic Biomarkers in Gastric and Colorectal Cancers. Reports of biochemistry & molecular biology. 2021;10(3):488-94. Epub 2022/01/05. doi: 10.52547/rbmb.10.3.488. PubMed PMID: 34981027; PubMed Central PMCID: PMCPMC8718786.

4. Abedpoor N, Taghian F, Hajibabaie F. Physical activity ameliorates the function of organs via adipose tissue in metabolic diseases. Acta Histochemica. 2022;124(2):151844. doi: https://dx.doi.org/10.1016/j.acthis.2022.151844.

5. Agra RM, Al-Daghri NM, Badimon L, Bodi V, Carbone F, Chen M, et al. Research update for articles published in EJCI in 2014. European Journal of Clinical Investigation. 2016;46(10):880-94. doi: https://dx.doi.org/10.1111/eci.12671.

6. Al-Daghri NM, Al-Attas OS, Alokail MS, Alkharfy KM, Yousef M, Vinodson B, et al. Maternal inheritance of circulating irisin in humans. Clinical Science. 2014;126(12):837-44. doi: https://dx.doi.org/10.1042/CS20130426.

7. Al-Daghri NM, Al-Attas OS, Alokail MS, Alkharfy KM, Yousef M, Vinodson B, et al. Maternal inheritance of circulating irisin in humans. Clinical science (London, England : 1979). 2014;126(12):837-44. Epub 2014/01/09. doi: 10.1042/cs20130426. PubMed PMID: 24397868.

8. Al-Daghri NM, Batzel JJ, Burgmann H, Carbone F, Charmandari E, Chrousos GP, et al. Research update for articles published in EJCI in 2015. European Journal of Clinical Investigation. 2017;47(10):775-88. doi: https://dx.doi.org/10.1111/eci.12819.

9. Algul S, Senol FF, Ugras S, Esmer F, Kara M, Ekici P, et al. Investigation of irisin level in brucella patients. Eastern Journal of Medicine. 2021;26(1):135-8. doi: https://dx.doi.org/10.5505/ejm.2021.70431.

10. Alizadeh Pahlavani H. Exercise Therapy for People With Sarcopenic Obesity: Myokines and Adipokines as Effective Actors. Frontiers in Endocrinology. 2022;13:811751. doi: https://dx.doi.org/10.3389/fendo.2022.811751.

11. Aljabban J, Khorfan K, Aljabban I, Aljabban H, Foflonker K, Panahiazar M, et al. Meta-analysis illustrates roles of cholesterol synthesis, dysregulated apoptosis, and other processes in nash pathogenesis. Hepatology. 2018;68(Supplement 1):747A. doi: https://dx.doi.org/10.1002/hep.30257.

12. Al-Rawaf HA, Alghadir AH, Gabr SA. Expression of Circulating MicroRNAs and Myokines and Interactions with Serum Osteopontin in Type 2 Diabetic Patients with Moderate and Poor Glycemic Control: A Biochemical and Molecular Study. BioMed Research International. 2021;2021:7453000. doi: https://dx.doi.org/10.1155/2021/7453000.

13. Altay DU, Keha EE, Karaguzel E, Mentese A, Yaman SO, Alver A. The Diagnostic Value of FNDC5/Irisin in Renal Cell Cancer. International braz j urol : official journal of the Brazilian Society of Urology. 2018;44(4):734-9. doi: https://dx.doi.org/10.1590/S1677-5538.IBJU.2017.0404.

14. Altay DU, Keha EE, Karagüzel E, Menteşe A, Yaman SO, Alver A. The Diagnostic Value of FNDC5/Irisin in Renal Cell Cancer. Int Braz J Urol. 2018;44(4):734-9. Epub 2018/03/10. doi: 10.1590/s1677-5538.ibju.2017.0404. PubMed PMID: 29522296; PubMed Central PMCID: PMCPMC6092672.

15. Altay DU, Keha EE, Yaman SO, Ince I, Alver A, Erdogan B, et al. Investigation of the expression of irisin and some cachectic factors in mice with experimentally induced gastric cancer. QJM. 2016;109(12):785-90. doi: https://dx.doi.org/10.1093/qjmed/hcw074.

16. Altlnova AE. Beige Adipocyte as the Flame of White Adipose Tissue: Regulation of Browning and Impact of Obesity. Journal of Clinical Endocrinology and Metabolism. 2022;107(5):E1778-E88. doi: https://dx.doi.org/10.1210/clinem/dgab921.

17. Alvarez AM, Deocesano-Pereira C, Teixeira C, Moreira V. Il-1beta and TNF-alpha modulation of proliferated and committed myoblasts: IL-6 and COX-2-derived prostaglandins as key actors in the mechanisms involved. Cells. 2020;9(9):1-17. doi: https://dx.doi.org/10.3390/cells9092005.

18. Amisi CA. Markers of insulin resistance in Polycystic ovary syndrome women: An update. World Journal of Diabetes. 2022;13(3):129-49. doi: https://dx.doi.org/10.4239/wjd.v13.i3.129.

19. Anand Narayanan S, Metzger CE, Bloomfield SA, Zawieja DC. Inflammation-induced lymphatic architecture and bone turnover changes are ameliorated by irisin treatment in chronic inflammatory bowel disease. FASEB Journal. 2018;32(9):4848-61. doi: https://dx.doi.org/10.1096/fj.201800178R.

20. Anaszewicz M, Wawrzenczyk A, Czerniak B, Banas W, Socha E, Lis K, et al. Leptin, adiponectin, tumor necrosis factor alpha, and irisin concentrations as factors linking obesity with the risk of atrial fibrillation among inpatients with cardiovascular diseases. Kardiologia Polska. 2019;77(11):1055-61. doi: https://dx.doi.org/10.33963/KP.14989.

21. Anaszewicz M, Wawrzeńczyk A, Czerniak B, Banaś W, Socha E, Lis K, et al. Leptin, adiponectin, tumor necrosis factor α, and irisin concentrations as factors linking obesity with the risk of atrial fibrillation among inpatients with cardiovascular diseases. Kardiol Pol. 2019;77(11):1055-61. Epub 2019/09/26. doi: 10.33963/kp.14989. PubMed PMID: 31553329.

22. Anonymous. ESICON 2016 Abstracts. Indian Journal of Endocrinology and Metabolism. 2017;21(7 Supplement 1).

23. Armamento-Villareal R, Napoli N, Waters D, Villareal D. Fat, Muscle, and Bone Interactions in Obesity and the Metabolic Syndrome. International Journal of Endocrinology. 2014;2014:247076. doi: https://dx.doi.org/10.1155/2014/247076.

24. Armandi A, Rosso C, Caviglia GP, Ribaldone DG, Bugianesi E. The Impact of Dysmetabolic Sarcopenia Among Insulin Sensitive Tissues: A Narrative Review. Frontiers in Endocrinology. 2021;12:716533. doi: https://dx.doi.org/10.3389/fendo.2021.716533.

25. Asghari E, Rashidlamir A, Hosseini SRA, Moazzami M, Samarghandian S, Farkhondeh T. Synergism effects of ursolic acid supplementation on the levels of irisin, C-reactive protein, IL-6, and TNF-alpha during high-intensity resistance training in low activity men. Cardiovascular and Hematological Disorders - Drug Targets. 2020;20(2):138-44. doi: https://dx.doi.org/10.2174/1871529X19666190918144727.

26. Asghari E, Rashidlamir A, Hosseini SRA, Moazzami M, Samarghandian S, Farkhondeh T. Synergism Effects of Ursolic Acid Supplementation on the Levels of Irisin, C-reactive Protein, IL-6, and TNF-α During High-intensity Resistance Training in Low Activity Men. Cardiovascular & hematological disorders drug targets. 2020;20(2):138-44. Epub 2019/09/20. doi: 10.2174/1871529x19666190918144727. PubMed PMID: 31533605.

27. Askari H, Rajani SF, Poorebrahim M, Haghi-Aminjan H, Raeis-Abdollahi E, Abdollahi M. A glance at the therapeutic potential of irisin against diseases involving inflammation, oxidative stress, and apoptosis: An introductory review. Pharmacological Research. 2018;129:44-55. doi: https://dx.doi.org/10.1016/j.phrs.2018.01.012.

28. Askari H, Rajani SF, Poorebrahim M, Haghi-Aminjan H, Raeis-Abdollahi E, Abdollahi M. A glance at the therapeutic potential of irisin against diseases involving inflammation, oxidative stress, and apoptosis: An introductory review. Pharmacol Res. 2018;129:44-55. Epub 2018/02/08. doi: 10.1016/j.phrs.2018.01.012. PubMed PMID: 29414191.

29. Aslan R, Alp HH, Eryilmaz R, Huyut Z, Sevim M, Araz S, et al. Can the Irisin be a Biomarker for Prostate Cancer? A Case Control Study. Asian Pacific journal of cancer prevention : APJCP. 2020;21(2):505-9. doi: https://dx.doi.org/10.31557/APJCP.2020.21.2.505.

30. Aslan R, Alp HH, Eryılmaz R, Huyut Z, Sevim M, Araz Ş, et al. Can the Irisin be a Biomarker for Prostate Cancer? A Case Control Study. Asian Pac J Cancer Prev. 2020;21(2):505-9. Epub 2020/02/28. doi: 10.31557/apjcp.2020.21.2.505. PubMed PMID: 32102531; PubMed Central PMCID: PMCPMC7332134.

31. Atila Uslu G, Uslu H. Evaluating the effects of Juglans regia L. extract on hyperglycaemia and insulin sensitivity in experimental type 2 diabetes in rat. Archives of physiology and biochemistry. 2022;128(1):121-5. doi: https://dx.doi.org/10.1080/13813455.2019.1668018.

32. Aydin S. Three new players in energy regulation: Preptin, adropin and irisin. Peptides. 2014;56:94-110. doi: https://dx.doi.org/10.1016/j.peptides.2014.03.021.

33. Aydin S. Is irisin a decisive protein in cancer cachexia and death of cancer cells? Eur Rev Med Pharmacol Sci. 2016;20(18):3727-9. Epub 2016/10/14. PubMed PMID: 27735050.

34. Aydin S, Eren MN, Kuloglu T, Aydin S, Yilmaz M, Gul E, et al. Alteration of serum and cardiac tissue adropin, copeptin, irisin and TRPM2 expressions in DOX treated male rats. Biotech Histochem. 2015;90(3):197-205. Epub 2014/12/02. doi: 10.3109/10520295.2014.977949. PubMed PMID: 25434395.

35. Aydin S, Eren MN, Kuloglu T, Yilmaz M, Gul E, Kalayci M, et al. Alteration of serum and cardiac tissue adropin, copeptin, irisin and TRPM2 expressions in DOX treated male rats. Biotechnic & histochemistry : official publication of the Biological Stain Commission. 2015;90(3):197-205. doi: https://dx.doi.org/10.3109/10520295.2014.977949.

36. Aydin S, Kuloglu T, Artas G, Kocdor MA, Sahin I, Yardim M, et al. Irisin a candidate biomarker for distinguishing thyroid oncocytic variant carcinomas. Turkish Journal of Biochemistry. 2017;42(Supplement 2):79.

37. Aydin S, Kuloglu T, Ozercan MR, Albayrak S, Aydin S, Bakal U, et al. Irisin immunohistochemistry in gastrointestinal system cancers. Biotech Histochem. 2016;91(4):242-50. Epub 2016/03/11. doi: 10.3109/10520295.2015.1136988. PubMed PMID: 26963139.

38. Babaei P, Hoseini R. Exercise training modulates adipokine dysregulations in metabolic syndrome. Sports Medicine and Health Science. 2022;4(1):18-28. doi: https://dx.doi.org/10.1016/j.smhs.2022.01.001.

39. Bagheri R, Rashidlamir A, Ashtary-Larky D, Wong A, Alipour M, Motevalli MS, et al. Does green tea extract enhance the anti-inflammatory effects of exercise on fat loss? British Journal of Clinical Pharmacology. 2020;86(4):753-62. doi: https://dx.doi.org/10.1111/bcp.14176.

40. Bagheri R, Rashidlamir A, Ashtary-Larky D, Wong A, Alipour M, Motevalli MS, et al. Does green tea extract enhance the anti-inflammatory effects of exercise on fat loss? Br J Clin Pharmacol. 2020;86(4):753-62. Epub 2019/11/21. doi: 10.1111/bcp.14176. PubMed PMID: 31747468; PubMed Central PMCID: PMCPMC7098875.

41. Bagheri R, Rashidlamir A, Ashtary-Larky D, Wong A, Grubbs B, Motevalli MS, et al. Effects of green tea extract supplementation and endurance training on irisin, pro-inflammatory cytokines, and adiponectin concentrations in overweight middle-aged men. European journal of applied physiology. 2020;120(4):915-23. doi: https://dx.doi.org/10.1007/s00421-020-04332-6.

42. Bagheri R, Rashidlamir A, Ashtary-Larky D, Wong A, Grubbs B, Motevalli MS, et al. Effects of green tea extract supplementation and endurance training on irisin, pro-inflammatory cytokines, and adiponectin concentrations in overweight middle-aged men. Eur

J Appl Physiol. 2020;120(4):915-23. Epub 2020/02/26. doi: 10.1007/s00421-020-04332-6. PubMed PMID: 32095935.

43. Bao ZH, Hou XB, Li HL, Mao YF, Wang WR. The mechanism and progress of ferroptosis in pancreatic cancer. Acta Histochemica. 2022;124(6):151919. doi: https://dx.doi.org/10.1016/j.acthis.2022.151919.

44. Batirel S, Bozaykut P, Mutlu Altundag E, Kartal Ozer N, Mantzoros CS. The effect of Irisin on antioxidant system in liver. Free radical biology & medicine. 2014;75 Suppl 1:S16. Epub 2015/10/16. doi: 10.1016/j.freeradbiomed.2014.10.592. PubMed PMID: 26461295.

45. Baumgartner M, Lischka J, Schanzer A, de Gier C, Walleczek NK, Greber-Platzer S, et al. Plasma Myostatin Increases with Age in Male Youth and Negatively Correlates with Vitamin D in Severe Pediatric Obesity. Nutrients. 2022;14(10):2133. doi: https://dx.doi.org/10.3390/nu14102133.

46. Bautmans I, Salimans L, Njemini R, Beyer I, Lieten S, Liberman K. The effects of exercise interventions on the inflammatory profile of older adults: A systematic review of the recent literature. Experimental Gerontology. 2021;146:111236. doi: https://dx.doi.org/10.1016/j.exger.2021.111236.

47. Ben-Zeev T, Okun E. High-Intensity Functional Training: Molecular Mechanisms and Benefits. NeuroMolecular Medicine. 2021;23(3):335-8. doi: https://dx.doi.org/10.1007/s12017-020-08638-8.

48. Berezin AE, Berezin AA, Lichtenauer M. Myokines and Heart Failure: Challenging Role in Adverse Cardiac Remodeling, Myopathy, and Clinical Outcomes. Disease Markers. 2021;2021:6644631. doi: https://dx.doi.org/10.1155/2021/6644631.

49. Bettis T, Kim BJ, Hamrick MW. Impact of muscle atrophy on bone metabolism and bone strength: implications for muscle-bone crosstalk with aging and disuse. Osteoporosis International. 2018;29(8):1713-20. doi: https://dx.doi.org/10.1007/s00198-018-4570-1.

50. Bhanji RA, Saiman Y, Watt KD. Nonalcoholic Steatohepatitis, Sarcopenia, and Liver Transplantation. Clinical Liver Disease. 2021;17(1):2-5. doi: https://dx.doi.org/10.1002/cld.1000.

51. Bi J, Ren Y, Du Z, Li Q, Wang Y, Wei S, et al. Irisin alleviates liver ischemia-reperfusion injury by inhibiting excessive mitochondrial fission, promoting mitochondrial biogenesis and decreasing oxidative stress. Redox Biology. 2019;20:296-306. doi: https://dx.doi.org/10.1016/j.redox.2018.10.019.

52. Bi J, Zhang J, Ren Y, Du Z, Li T, Wang T, et al. Irisin reverses intestinal epithelial barrier dysfunction during intestinal injury via binding to the integrin alphaVbeta5 receptor. Journal of Cellular and Molecular Medicine. 2020;24(1):996-1009. doi: https://dx.doi.org/10.1111/jcmm.14811.

53. Bilski J, Mazur-Bialy A, Hubalewska-Mazgaj M, Brzozowski B, Surmiak M, Wojcik D, et al. Role of gut-adipose-muscle axis in beneficial effect of voluntary exercise on experimental colitis in mice fed a diet-induced obesity. Involvement of protective irisin and proinflammatory biomarkers released from mesenteric fat and colonic mucosa. Gastroenterology. 2017;152(5 Supplement 1):S828.

54. Bilski J, Mazur-Bialy A, Magierowski M, Wojcik D, Brzozowski B, Sliwowski Z, et al. Voluntary exercise improves the healing of experimental colitis in mice fed diet-induced obesity. Essential role of endocrine factors released from skeletal muscles and adipose tissue. United European Gastroenterology Journal. 2016;4(5 Supplement 1):A240. doi: https://dx.doi.org/10.1177/2050640616663689.

55. Bilski J, Mazur-Bialy AI, Brzozowski B, Magierowski M, Jasnos K, Krzysiek-Maczka G, et al. Moderate exercise training attenuates the severity of experimental rodent colitis: The importance of crosstalk between adipose tissue and skeletal muscles. Mediators of Inflammation. 2015;2015:605071. doi: https://dx.doi.org/10.1155/2015/605071.

56. Bilski J, Mazur-Bialy AI, Wierdak M, Brzozowski T. The impact of physical activity and nutrition on inflammatory bowel disease: The potential role of cross talk between adipose tissue and skeletal muscle. Journal of Physiology and Pharmacology. 2013;64(2).

57. Bilski J, Surmiak M, Mazur-Bialy A, Magierowski M, Wojcik D, Hubalewska-Mazgaj M, et al. Role of factors released from adipose tissue, cytokines and myokines in forced treadmill exerciseinduced exacerbation of experimental colitis in mice fed standard diet and diet-induced obesity. Relevance of physical activity to the course of IBD in humans. United European Gastroenterology Journal. 2018;6(8 Supplement):A632. doi: https://dx.doi.org/10.1177/2050640618792819.

58. Bluher S, Panagiotou G, Petroff D, Markert J, Wagner A, Klemm T, et al. Effects of a 1-year exercise and lifestyle intervention on irisin, adipokines, and inflammatory markers in obese children. Obesity. 2014;22(7):1701-8. doi: https://dx.doi.org/10.1002/oby.20739.

59. Blüher S, Panagiotou G, Petroff D, Markert J, Wagner A, Klemm T, et al. Effects of a 1-year exercise and lifestyle intervention on irisin, adipokines, and inflammatory markers in obese children. Obesity (Silver Spring, Md). 2014;22(7):1701-8. Epub 2014/03/20. doi: 10.1002/oby.20739. PubMed PMID: 24644099.

60. Bo W, Xi Y, Tian Z. The role of exercise in rehabilitation of discharged COVID-19 patients. Sports Medicine and Health Science. 2021;3(4):194-201. doi: https://dx.doi.org/10.1016/j.smhs.2021.09.001.

61. Bonfante ILP, Chacon-Mikahil MPT, Brunelli DT, Gaspari AF, Duft RG, Oliveira AG, et al. Obese with higher FNDC5/Irisin levels have a better metabolic profile, lower lipopolysaccharide levels and type 2 diabetes risk. Archives of endocrinology and metabolism. 2017;61(6):524-33. doi: https://dx.doi.org/10.1590/2359-3997000000305.

62. Bonfante ILP, Chacon-Mikahil MPT, Brunelli DT, Gáspari AF, Duft RG, Oliveira AG, et al. Obese with higher FNDC5/Irisin levels have a better metabolic profile, lower lipopolysaccharide levels and type 2 diabetes risk. Arch Endocrinol Metab. 2017;61(6):524-33. Epub 2018/02/08. doi: 10.1590/2359-3997000000305. PubMed PMID: 29412381.

63. Bosma M, Gerling M, Pasto J, Georgiadi A, Graham E, Shilkova O, et al. FNDC4 acts as an anti-inflammatory factor on macrophages and improves colitis in mice. Nature Communications. 2016;7:11314. doi: https://dx.doi.org/10.1038/ncomms11314.

64. Bretland KA, Lin L, Bretland KM, Smith MA, Fleming SM, Dengler-Crish CM. Irisin treatment lowers levels of phosphorylated tau in the hippocampus of pre-symptomatic

female but not male htau mice. Neuropathology and Applied Neurobiology. 2021;47(7):967-78. doi: https://dx.doi.org/10.1111/nan.12711.

65. Bretland KA, Lin L, Bretland KM, Smith MA, Fleming SM, Dengler-Crish CM. Irisin treatment lowers levels of phosphorylated tau in the hippocampus of pre-symptomatic female but not male htau mice. Neuropathol Appl Neurobiol. 2021;47(7):967-78. Epub 2021/03/27. doi: 10.1111/nan.12711. PubMed PMID: 33768561; PubMed Central PMCID: PMCPMC9292848.

66. Bulut T, Yalcin AD, Celik B, Genc GE, Kose S, Gocmen AY, et al. Omalizumab treatment decreased IL-1beta and irisin but increased chemerin without any impact on NK cells and APC cells in cases of severe asthma. Allergy: European Journal of Allergy and Clinical Immunology. 2016;71(Supplement 102):257. doi: https://dx.doi.org/10.1111/all.12972.

67. Buscemi S, Corleo D, Vasto S, Buscemi C, Barile AM, Rosafio G, et al. Serum Irisin Concentrations in Severely Inflamed Patients. Hormone and Metabolic Research. 2020;52(4):246-50. doi: https://dx.doi.org/10.1055/a-1111-9249.

68. Buscemi S, Corleo D, Vasto S, Buscemi C, Barile AM, Rosafio G, et al. Serum Irisin Concentrations in Severely Inflamed Patients. Hormone and metabolic research = Hormon- und Stoffwechselforschung = Hormones et metabolisme. 2020;52(4):246-50. Epub 2020/02/23. doi: 10.1055/a-1111-9249. PubMed PMID: 32079027.

69. Butt ZD, Hackett JD, Volkoff H. Irisin in goldfish (Carassius auratus): Effects of irisin injections on feeding behavior and expression of appetite regulators, uncoupling proteins and lipoprotein lipase, and fasting-induced changes in FNDC5 expression. Peptides. 2017;90:27-36. doi: https://dx.doi.org/10.1016/j.peptides.2017.02.003.

70. Cai C, Qian L, Jiang S, Sun Y, Wang Q, Ma D, et al. Loss-of-function myostatin mutation increases insulin sensitivity and browning of white fat in Meishan pigs. Oncotarget. 2017;8(21):34911-22. doi: https://dx.doi.org/10.18632/oncotarget.16822.

71. Carsote M, Albu SE, Ghemigian A, Valea A. Particular types of non - Age related sarcopenia: Osteosarcopenic obesity and malignancy - Associated muscle waste (a mini-review). Archives of the Balkan Medical Union. 2016;51(3):369-72.

72. Castillo EC, Elizondo-Montemayor L, Hernandez-Brenes C, Rodriguez-Sanchez DG, Silva-Platas C, Marin-Obispo LM, et al. Integrative analysis of lipid profiles in plasma allows cardiometabolic risk factor clustering in children with metabolically unhealthy obesity. Oxidative Medicine and Cellular Longevity. 2020;2020:2935278. doi: https://dx.doi.org/10.1155/2020/2935278.

73. Cebulski K, Nowinska K, Jablonska K, Romanowicz H, Smolarz B, Dziegiel P, et al. Expression of Irisin/FNDC5 in Breast Cancer. International Journal of Molecular Sciences. 2022;23(7):3530. doi: https://dx.doi.org/10.3390/ijms23073530.

74. Cebulski K, Nowińska K, Jablońska K, Romanowicz H, Smolarz B, Dzięgiel P, et al. Expression of Irisin/FNDC5 in Breast Cancer. Int J Mol Sci. 2022;23(7). Epub 2022/04/13. doi: 10.3390/ijms23073530. PubMed PMID: 35408891; PubMed Central PMCID: PMCPMC8998925.

75. Chakravarthy MV, Siddiqui MS, Forsgren MF, Sanyal AJ. Harnessing Muscle-Liver Crosstalk to Treat Nonalcoholic Steatohepatitis. Frontiers in Endocrinology. 2020;11:592373. doi: https://dx.doi.org/10.3389/fendo.2020.592373.

76. Chen JR, Yeh WJ, Tan HY, Yang HY. Antroquinonol Attenuated Abdominal and Hepatic Fat Accumulation in Rats Fed an Obesogenic Diet. Journal of food science. 2019;84(9):2682-7. doi: https://dx.doi.org/10.1111/1750-3841.14746.

77. Chen T, Lin Y, Wu Z, Shi H, Hu W, Li S, et al. Irisin Ameliorates Intervertebral Disc Degeneration by Activating LATS/YAP/CTGF Signaling. Oxidative Medicine and Cellular Longevity. 2022;2022:9684062. doi: https://dx.doi.org/10.1155/2022/9684062.

78. Chen X, Hodges PW, James G, Diwan AD. Do markers of inflammation and/or muscle regeneration in lumbar multifidus muscle and fat differ between individuals with good or poor outcome following microdiscectomy for lumbar disc herniation? Spine. 2021;46(10):678-86. doi: https://dx.doi.org/10.1097/BRS.0000000000003863.

79. Cheng CF, Ku HC, Lin H. Pgc-1alpha as a pivotal factor in lipid and metabolic regulation. International Journal of Molecular Sciences. 2018;19(11):3447. doi: https://dx.doi.org/10.3390/ijms19113447.

80. Cheng CF, Ku HC, Lin H. PGC-1α as a Pivotal Factor in Lipid and Metabolic Regulation. Int J Mol Sci. 2018;19(11). Epub 2018/11/08. doi: 10.3390/ijms19113447. PubMed PMID: 30400212; PubMed Central PMCID: PMCPMC6274980.

81. Cheng G, Xu D, Chu K, Cao Z, Sun X, Yang Y. The Effects of MiR-214-3p and Irisin/FNDC5 on the Biological Behavior of Osteosarcoma Cells. Cancer Biotherapy and Radiopharmaceuticals. 2020;35(2):92-100. doi: https://dx.doi.org/10.1089/cbr.2019.2933.

82. Cheng G, Xu D, Chu K, Cao Z, Sun X, Yang Y. The Effects of MiR-214-3p and Irisin/FNDC5 on the Biological Behavior of Osteosarcoma Cells. Cancer biotherapy & radiopharmaceuticals. 2020;35(2):92-100. Epub 2020/02/20. doi: 10.1089/cbr.2019.2933. PubMed PMID: 32073886.

83. Cheng YY, Zhang P, Tan XY. Expression of Irisin in cervical cancer tissues and its clinical significance. Chinese Journal of Cancer Prevention and Treatment. 2017;24(21):1523-9.

84. Chiu H, Chang W, Lin Y, Chung Y, Yen T, Huang F, et al. Irisin, an exercise-induced hormone, targets glioblastoma tumor in vivo PET/CT imaging may serve as a novel theranostic agent. European Journal of Nuclear Medicine and Molecular Imaging. 2017;44(2 Supplement 1):S468. doi: https://dx.doi.org/10.1007/s00259-017-3822-1.

85. Christodoulatos GS, Spyrou N, Kadillari J, Psallida S, Dalamaga M. The Role of Adipokines in Breast Cancer: Current Evidence and Perspectives. Current Obesity Reports. 2019;8(4):413-33. doi: https://dx.doi.org/10.1007/s13679-019-00364-y.

86. Christodoulatos GS, Spyrou N, Kadillari J, Psallida S, Dalamaga M. The Role of Adipokines in Breast Cancer: Current Evidence and Perspectives. Curr Obes Rep. 2019;8(4):413-33. Epub 2019/10/23. doi: 10.1007/s13679-019-00364-y. PubMed PMID: 31637624.

87. Chung HS, Choi KM. Adipokines and myokines: A pivotal role in metabolic and cardiovascular disorders. Current Medicinal Chemistry. 2018;25(20):2401-15. doi: https://dx.doi.org/10.2174/0929867325666171205144627.

88. Clark IA, Vissel B. Neurodegenerative disease treatments by direct TNF reduction, SB623 cells, maraviroc and irisin and MCC950, from an inflammatory perspective-a Commentary. Expert Review of Neurotherapeutics. 2019;19(6):535-43. doi: https://dx.doi.org/10.1080/14737175.2019.1618710.

89. Clark IA, Vissel B. Neurodegenerative disease treatments by direct TNF reduction, SB623 cells, maraviroc and irisin and MCC950, from an inflammatory perspective - a Commentary. Expert Rev Neurother. 2019;19(6):535-43. Epub 2019/05/17. doi: 10.1080/14737175.2019.1618710. PubMed PMID: 31092047.

90. Coletta AM, Agha NH, Baker FL, Niemiro GM, Mylabathula PL, Brewster AM, et al. The impact of high-intensity interval exercise training on NK-cell function and circulating myokines for breast cancer prevention among women at high risk for breast cancer. Breast Cancer Research and Treatment. 2021;187(2):407-16. doi: https://dx.doi.org/10.1007/s10549-021-06111-z.

91. Coletta AM, Agha NH, Baker FL, Niemiro GM, Mylabathula PL, Brewster AM, et al. The impact of high-intensity interval exercise training on NK-cell function and circulating myokines for breast cancer prevention among women at high risk for breast cancer. Breast Cancer Res Treat. 2021;187(2):407-16. Epub 2021/02/09. doi: 10.1007/s10549-021-06111-z. PubMed PMID: 33555464; PubMed Central PMCID: PMCPMC8189992.

92. Colucci S, Colaianni G, Brunetti G, Ferranti F, Mascetti G, Mori G, et al. Irisin prevents microgravity-induced impairment of osteoblast differentiation in vitro during the space flight CRS-14 mission. FASEB Journal. 2020;34(8):10096-106. doi: https://dx.doi.org/10.1096/fj.202000216R.

93. Comassi M, Vitolo E, Pratali L, Del Turco S, Dellanoce C, Rossi C, et al. Acute effects of different degrees of ultra-endurance exercise on systemic inflammatory responses. Internal Medicine Journal. 2015;45(1):74-9. doi: https://dx.doi.org/10.1111/imj.12625.

94. Comassi M, Vitolo E, Pratali L, Del Turco S, Dellanoce C, Rossi C, et al. Acute effects of different degrees of ultra-endurance exercise on systemic inflammatory responses. Intern Med J. 2015;45(1):74-9. Epub 2014/11/06. doi: 10.1111/imj.12625. PubMed PMID: 25371101.

95. Correa MP, Burrows R, Albala C, Salech F, Sanhueza G, Christian GB. THE OBAGE STUDY: A PROSPECTIVE CASE-CONTROL STUDY EMBEDDED IN A PROSPECTIVE BIRTH COHORT TO IDENTIFY SYSTEMIC, CELLULAR, AND MOLECULAR BIOMARKERS OF OBESITY-INDUCED ACCELERATED AGING IN 30-YEARS OLD CHILEAN ADULTS. Journal of Nutrition, Health and Aging. 2022;26(4):472-3. doi: https://dx.doi.org/10.1007/s12603-022-1772-1.

96. Crujeiras AB, Pardo M, Roca-Rivada A, Navas-Carretero S, Zulet MA, Martinez JA, et al. Longitudinal variation of circulating irisin after an energy restriction-induced weight loss and following weight regain in obese men and women. American Journal of Human Biology. 2014;26(2):198-207. doi: https://dx.doi.org/10.1002/ajhb.22493.

97. Crujeiras AB, Zulet MA, Lopez-Legarrea P, De La Iglesia R, Pardo M, Carreira MC, et al. Association between circulating irisin levels and the promotion of insulin resistance during

the weight maintenance period after a dietary weight-lowering program in obese patients. Metabolism: Clinical and Experimental. 2014;63(4):520-31. doi: https://dx.doi.org/10.1016/j.metabol.2013.12.007.

98. Curcio F, Ferro G, Basile C, Liguori I, Parrella P, Pirozzi F, et al. Biomarkers in sarcopenia: A multifactorial approach. Experimental Gerontology. 2016;85:1-8. doi: https://dx.doi.org/10.1016/j.exger.2016.09.007.

99. Daas SI, Rizeq BR, Nasrallah GK. Adipose tissue dysfunction in cancer cachexia. Journal of Cellular Physiology. 2018;234(1):13-22. doi: https://dx.doi.org/10.1002/jcp.26811.

100. Daneshi-Maskooni M, Keshavarz SA, Mansouri S, Qorbani M, Alavian SM, Badri-Fariman M, et al. The effects of green cardamom on blood glucose indices, lipids, inflammatory factors, paraxonase-1, sirtuin-1, and irisin in patients with nonalcoholic fatty liver disease and obesity: Study protocol for a randomized controlled trial. Trials. 2017;18(1):260. doi: https://dx.doi.org/10.1186/s13063-017-1979-3.

101. Danielak A, Wojcik D, Mazur-Bialy A, Surmiak M, Bilski J, Targosz A, et al. Intestinal Alkaline Phosphatase Combined with Voluntary Physical Activity Alleviates Experimental Colitis in Obese Mice. Involvement of Oxidative Stress, Myokines, Adipokines and Proinflammatory Biomarkers. Antioxidants (Basel, Switzerland). 2021;10(2). Epub 2021/02/10. doi: 10.3390/antiox10020240. PubMed PMID: 33557311; PubMed Central PMCID: PMCPMC7914798.

102. Darkwah S, Park EJ, Myint PK, Ito A, Appiah MG, Obeng G, et al. Potential Roles of Muscle-Derived Extracellular Vesicles in Remodeling Cellular Microenvironment: Proposed Implications of the Exercise-Induced Myokine, Irisin. Frontiers in Cell and Developmental Biology. 2021;9:634853. doi: https://dx.doi.org/10.3389/fcell.2021.634853.

103. Darkwah S, Park EJ, Myint PK, Ito A, Appiah MG, Obeng G, et al. Potential Roles of Muscle-Derived Extracellular Vesicles in Remodeling Cellular Microenvironment: Proposed Implications of the Exercise-Induced Myokine, Irisin. Front Cell Dev Biol. 2021;9:634853. Epub 2021/02/23. doi: 10.3389/fcell.2021.634853. PubMed PMID: 33614663; PubMed Central PMCID: PMCPMC7892973.

104. de Castro GS, Correia-Lima J, Simoes E, Orsso CE, Xiao J, Gama LR, et al. Myokines in treatment-naive patients with cancer-associated cachexia. Clinical Nutrition. 2021;40(4):2443-55. doi: https://dx.doi.org/10.1016/j.clnu.2020.10.050.

105. de Castro GS, Correia-Lima J, Simoes E, Orsso CE, Xiao J, Gama LR, et al. Myokines in treatment-naïve patients with cancer-associated cachexia. Clinical nutrition (Edinburgh, Scotland). 2021;40(4):2443-55. Epub 2020/11/17. doi: 10.1016/j.clnu.2020.10.050. PubMed PMID: 33190987.

106. de Sousa CAZ, Sierra APR, Martinez Galan BS, Maciel JFDS, Manoel R, Barbeiro HV, et al. Time Course and Role of Exercise-Induced Cytokines in Muscle Damage and Repair After a Marathon Race. Frontiers in Physiology. 2021;12:752144. doi: https://dx.doi.org/10.3389/fphys.2021.752144.

107. de Sousa CAZ, Sierra APR, Martínez Galán BS, Maciel JFS, Manoel R, Barbeiro HV, et al. Time Course and Role of Exercise-Induced Cytokines in Muscle Damage and Repair After a Marathon Race. Front Physiol. 2021;12:752144. Epub 2021/11/02. doi:

10.3389/fphys.2021.752144. PubMed PMID: 34721075; PubMed Central PMCID: PMCPMC8554198.

108. De Sousa Gehrke F, Gouveia MC, Barbosa CGM, Murad N, Da Costa Aguiar Alves Reis B, Fonseca FLA, et al. Irisin and troponin i expression in dialysis patients submitted to remote ischemic preconditioning: A pilot study. Jornal Brasileiro de Nefrologia. 2020;42(1):4752. doi: https://dx.doi.org/10.1590/2175-8239-JBN-2019-0051.

109. Dechaumet B, Cleret D, Laroche N, Vanden-Bossche A, Lafage-Proust MH, Vico L. Is chronic hypergravity able to protect the musculoskeletal system in a murine model of knee osteoarthritis? Journal of Bone and Mineral Research. 2018;33(Supplement 1):359.

110. Dechaumet B, Cleret D, Linossier MT, Vanden-Bossche A, Chanon S, Lefai E, et al. Hypergravity as a gravitational therapy mitigates the effects of knee osteoarthritis on the musculoskeletal system in a murine model. PLoS ONE. 2020;15(12 December):e0243098. doi: https://dx.doi.org/10.1371/journal.pone.0243098.

111. Deibert C, Ferrari N, Flock A, Merz WM, Gembruch U, Lehmacher W, et al. Adipokine-myokine-hepatokine compartment-system in mothers and children: An explorative study. Contemporary Clinical Trials Communications. 2016;3:1-5. doi: https://dx.doi.org/10.1016/j.conctc.2016.02.002.

112. Deng CY, Zhu TT, Lian S, Wang JF, Wu R, Zheng JS. Estrogen-related receptor alpha (ERRalpha) functions in the hypoxic injury of microglial cells. Journal of Veterinary Research (Poland). 2022;66(1):131-40. doi: https://dx.doi.org/10.2478/jvetres-2022-0009.

113. Deng J, Zhang N, Chen F, Yang C, Ning H, Xiao C, et al. Irisin ameliorates high glucose-induced cardiomyocytes injury via AMPK/mTOR signal pathway. Cell Biology International. 2020;44(11):2315-25. doi: https://dx.doi.org/10.1002/cbin.11441.

114. Deng J, Zhang N, Chen F, Yang C, Ning H, Xiao C, et al. Irisin ameliorates high glucose-induced cardiomyocytes injury via AMPK/mTOR signal pathway. Cell Biol Int. 2020;44(11):2315-25. Epub 2020/08/10. doi: 10.1002/cbin.11441. PubMed PMID: 32770767.

115. Diaz BB, Gonzalez DA, Gannar F, Perez MCR, de Leon AC. Myokines, physical activity, insulin resistance and autoimmune diseases. Immunology Letters. 2018;203:1-5. doi: https://dx.doi.org/10.1016/j.imlet.2018.09.002.

116. Díaz BB, González DA, Gannar F, Pérez MCR, de León AC. Myokines, physical activity, insulin resistance and autoimmune diseases. Immunol Lett. 2018;203:1-5. Epub 2018/09/09. doi: 10.1016/j.imlet.2018.09.002. PubMed PMID: 30194964.

117. Dimberg J, Shamoun L, Landerholm K, Wagsater D. Effects of diabetes type 2 and metformin treatment in Swedish patients with colorectal cancer. World Journal of Gastroenterology. 2022;28(19):2148-51. doi: https://dx.doi.org/10.3748/wjg.v28.i19.2148.

118. Dimberg J, Shamoun L, Landerholm K, Wågsäter D. Effects of diabetes type 2 and metformin treatment in Swedish patients with colorectal cancer. World J Gastroenterol. 2022;28(19):2148-51. Epub 2022/06/07. doi: 10.3748/wjg.v28.i19.2148. PubMed PMID: 35664033; PubMed Central PMCID: PMCPMC9134133.

119. Dobrodeeva LK, Samodova AV, Balashova SN. Cellular reactions in capillary and venous blood in northerners to a short-term period in a climatic chamber. Immunity, Inflammation and Disease. 2020;8(3):408-14. doi: https://dx.doi.org/10.1002/iid3.322.

120. Dobrodeeva LK, Samodova AV, Balashova SN. Cellular reactions in capillary and venous blood in northerners to a short-term period in a climatic chamber. Immun Inflamm Dis. 2020;8(3):408-14. Epub 2020/06/20. doi: 10.1002/iid3.322. PubMed PMID: 32558359; PubMed Central PMCID: PMCPMC7416046.

121. Domaszewska K, Boraczynski M, Tang YY, Gronek J, Wochna K, Boraczynski T, et al. Protective Effects of Exercise Become Especially Important for the Aging Immune System in The Covid-19 Era. Aging and Disease. 2022;13(1):129-43. doi: https://dx.doi.org/10.14336/AD.2021.1219.

122. Dos Santos ARO, Zanuso BO, Miola VFB, Barbalho SM, Santos Bueno PC, Flato UAP, et al. Adipokines, myokines, and hepatokines: Crosstalk and metabolic repercussions. International Journal of Molecular Sciences. 2021;22(5):1-23. doi: https://dx.doi.org/10.3390/ijms22052639.

123. Dos Santos JPM, de Maio MC, Lemes MA, Laurindo LF, Haber JFS, Bechara MD, et al. Non-alcoholic steatohepatitis (NASH) and organokines: What is now and what will be in the future. International Journal of Molecular Sciences. 2022;23(1):498. doi: https://dx.doi.org/10.3390/ijms23010498.

124. Drabik A, Mudlaff K, Silberring J, Kulig J, Sierzega M. New method of Irisin levels identification in gastric cancer patients with cachexia. FEBS Journal. 2017;284(Supplement 1):284. doi: https://dx.doi.org/10.1111/febs.14174.

125. Du J, Fan X, Yang B, Chen Y, Liu KX, Zhou J. Irisin pretreatment ameliorates intestinal ischemia/reperfusion injury in mice through activation of the Nrf2 pathway. International Immunopharmacology. 2019;73:225-35. doi: https://dx.doi.org/10.1016/j.intimp.2019.05.011.

126. Du J, Fan X, Yang B, Chen Y, Liu KX, Zhou J. Irisin pretreatment ameliorates intestinal ischemia/reperfusion injury in mice through activation of the Nrf2 pathway. Int Immunopharmacol. 2019;73:225-35. Epub 2019/05/21. doi: 10.1016/j.intimp.2019.05.011. PubMed PMID: 31108387.

127. Duffles LF, Hermont AP, Abreu LG, Pordeus IA, Silva TA. Association between obesity and adipokines levels in saliva and gingival crevicular fluid: A systematic review and meta-analysis. Journal of Evidence-Based Medicine. 2019;12(4):313-24. doi: https://dx.doi.org/10.1111/jebm.12363.

128. Dunnwald T, Melmer A, Gatterer H, Salzmann K, Ebenbichler C, Burtscher M, et al. Supervised Short-term High-intensity Training on Plasma Irisin Concentrations in Type 2 Diabetic Patients. International journal of sports medicine. 2019;40(3):158-64. doi: https://dx.doi.org/10.1055/a-0828-8047.

129. Dupuis C, Berthon JY, Larue J, Rouge S, Filaire M, Filaire E. Effects of 6 weeks of betaine or C-phycocyanin supplementation associated or not with wheel running on redox status. Science and Sports. 2018;33(1):47-55. doi: https://dx.doi.org/10.1016/j.scispo.2017.08.006.

130. El Rabey HA, Rezk SM, Sakran MI, Mohammed GM, Bahattab O, Balgoon MJ, et al. Green coffee methanolic extract and silymarin protect against CCl4-induced hepatotoxicity in albino male rats. BMC Complementary Medicine and Therapies. 2021;21(1):19. doi: https://dx.doi.org/10.1186/s12906-020-03186-x.

131. Elizondo-Montemayor L, Gonzalez-Gil AM, Tamez-Rivera O, Toledo-Salinas C, Peschard-Franco M, Rodriguez-Gutierrez NA, et al. Association between irisin, hs-CRP, and metabolic status in children and adolescents with type 2 diabetes mellitus. Mediators of Inflammation. 2019;2019:6737318. doi: https://dx.doi.org/10.1155/2019/6737318.

132. Emami MR, Alipoor E, Hosseinzadeh-Attar MJ. Irisin - A potential contributor of insulin resistance in kidney failure. European Journal of Internal Medicine. 2017;44:e22-e3. doi: https://dx.doi.org/10.1016/j.ejim.2017.07.030.

133. Ercan Z, Deniz G, Yentur SB, Arikan FB, Karatas A, Alkan G, et al. Effects of acute aerobic exercise on cytokines, klotho, irisin, and vascular endothelial growth factor responses in rheumatoid arthritis patients. Irish Journal of Medical Science. 2022. doi: https://dx.doi.org/10.1007/s11845-022-02970-7.

134. Ercan Z, Deniz G, Yentur SB, Arikan FB, Karatas A, Alkan G, et al. Effects of acute aerobic exercise on cytokines, klotho, irisin, and vascular endothelial growth factor responses in rheumatoid arthritis patients. Ir J Med Sci. 2022. Epub 2022/03/18. doi: 10.1007/s11845-022-02970-7. PubMed PMID: 35296975.

135. Esawy MM, Abdel-Samd KM. The diagnostic and prognostic roles of serum irisin in bladder cancer. Current Problems in Cancer. 2020;44(4):100529. doi: https://dx.doi.org/10.1016/j.currproblcancer.2019.100529.

136. Esawy MM, Abdel-Samd KM. The diagnostic and prognostic roles of serum irisin in bladder cancer. Curr Probl Cancer. 2020;44(4):100529. Epub 2020/03/07. doi: 10.1016/j.currproblcancer.2019.100529. PubMed PMID: 32139156.

137. Eser N, Yoldas A, Turk A, Kalaycı Yigin A, Yalcin A, Cicek M. Ameliorative effects of garlic oil on FNDC5 and irisin sensitivity in liver of streptozotocin-induced diabetic rats. The Journal of pharmacy and pharmacology. 2021;73(6):824-34. Epub 2021/03/20. doi: 10.1093/jpp/rgab023. PubMed PMID: 33739409.

138. Eser N, Yoldas A, Turk A, Kalaycl Yigin A, Yalcin A, Cicek M. Ameliorative effects of garlic oil on FNDC5 and irisin sensitivity in liver of streptozotocin-induced diabetic rats. Journal of Pharmacy and Pharmacology. 2021;73(6):824-34. doi: https://dx.doi.org/10.1093/jpp/rgab023.

139. Eskandari M, Moghadam BH, Bagheri R, Ashtary-Larky D, Eskandari E, Nordvall M, et al. Effects of interval jump rope exercise combined with dark chocolate supplementation on inflammatory adipokine, cytokine concentrations, and body composition in obese adolescent boys. Nutrients. 2020;12(10):1-16. doi: https://dx.doi.org/10.3390/nu12103011.

140. Etnier JL, Wideman L, Karper WB, Labban JD, Wahlheim CN, Williams TM, et al. Physical activity and Alzheimer's disease-2: Clinical trial protocol. Journal of Prevention of Alzheimer's Disease. 2019;6(Supplement 1):S50. doi: https://dx.doi.org/10.14283/jpad.2019.48.

141. Fabian SG. The skeletal muscle-metabolism axis in prostate-cancer therapy. New England Journal of Medicine. 2012;367(23):2257-8. doi: https://dx.doi.org/10.1056/NEJMc1211955.

142. Fan GH, Zhu TY, Huang J. FNDC5 promotes paclitaxel sensitivity of non-small cell lung cancers via inhibiting MDR1. Cellular Signalling. 2020;72:109665. doi: https://dx.doi.org/10.1016/j.cellsig.2020.109665.

143. Fan X, Du J, Wang MH, Li JM, Yang B, Chen Y, et al. Irisin contributes to the hepatoprotection of dexmedetomidine during intestinal ischemia/reperfusion. Oxidative Medicine and Cellular Longevity. 2019;2019:7857082. doi: https://dx.doi.org/10.1155/2019/7857082.

144. Fan X, Du J, Wang MH, Li JM, Yang B, Chen Y, et al. Irisin Contributes to the Hepatoprotection of Dexmedetomidine during Intestinal Ischemia/Reperfusion. Oxid Med Cell Longev. 2019;2019:7857082. Epub 2019/06/14. doi: 10.1155/2019/7857082. PubMed PMID: 31191804; PubMed Central PMCID: PMCPMC6525857.

145. Farr OM, Mantzoros CS. Sleep apnea in relation to metabolism: An urgent need to study underlying mechanisms and to develop novel treatments for this unmet clinical need. Metabolism: Clinical and Experimental. 2017;69:207-10. doi: https://dx.doi.org/10.1016/j.metabol.2017.01.028.

146. Febbraio MA, Pedersen BK. Who would have thought - myokines two decades on. Nature Reviews Endocrinology. 2020;16(11):619-20. doi: https://dx.doi.org/10.1038/s41574-020-00408-7.

147. Fei Y, Guo P, Wang F, Li H, Lei Y, Li W, et al. Identification of miRNA-mRNA crosstalk in laryngeal squamous cell carcinoma. Molecular Medicine Reports. 2017;16(4):4179-86. doi: https://dx.doi.org/10.3892/mmr.2017.7123.

148. Fernandez-Mincone T, Contreras-Briceno F, Espinosa-Ramirez M, Garcia-Valdes P, Lopez-Fuenzalida A, Riquelme A, et al. Nonalcoholic fatty liver disease and sarcopenia: pathophysiological connections and therapeutic implications. Expert Review of Gastroenterology and Hepatology. 2020;14(12):1141-57. doi: https://dx.doi.org/10.1080/17474124.2020.1810563.

149. Formigari GP, Datilo MN, Lopes De Faria JM, Lopes De Faria JB. The role of the Irisin-AMPK axis in the improvement of diabetic nephropathy in exercised rats. Diabetologia. 2020;63(SUPPL 1):S90-S1. doi: https://dx.doi.org/10.1007/s00125-020-05221-5.

150. Fornelli G, Isaia GC, D'Amelio P. Ageing, muscle and bone. Journal of Gerontology and Geriatrics. 2016;64(3):75-80.

151. Fraga VG, Ferreira CN, Oliveira FR, Candido AL, das Gracas Carvalho M, Reis FM, et al. Irisin levels are correlated with inflammatory markers in frontotemporal dementia. Journal of Clinical Neuroscience. 2021;93:92-5. doi: https://dx.doi.org/10.1016/j.jocn.2021.09.005.

152. Frille A, Kuhn H, Ebert T, Seyfarth HJ, Wirtz H. Non-small cell lung cancer cells induce the expression of adipokines in brown adipose tissue in the context of cancer cachexia. Pneumologie. 2019;73(SUPPPL. 1). doi: https://dx.doi.org/10.1055/s-0039-1678077.

153. Frille A, Kuhn H, Ebert T, Seyfarth HJ, Wirtz H. Brown adipose tissue impairs chemosensitivity in non-small cell lung cancer cells in the context of cancer cachexia. Pneumologie. 2019;73(SUPPPL. 1). doi: https://dx.doi.org/10.1055/s-0039-1678076.

154. Frille A, Kuhn H, Ebert T, Seyfarth HJ, Wirtz H. Brown adipose tissue derived media impairs chemosensitivity in non-small lung cancer cells in the context of cancer cachexia. Pneumologie. 2019;73(3):189. doi: https://dx.doi.org/10.1055/s-0038-1676413.

155. Fruhbeck G, Fernandez-Quintana B, Paniagua M, Hernandez-Pardos AW, Valenti V, Moncada R, et al. FNDC4, a novel adipokine that reduces lipogenesis and promotes fat browning in human visceral adipocytes. Metabolism: Clinical and Experimental. 2020;108:154261. doi: https://dx.doi.org/10.1016/j.metabol.2020.154261.

156. Gaggini M, Cabiati M, Del Turco S, Navarra T, De Simone P, Filipponi F, et al. Increased FNDC5/Irisin expression in human hepatocellular carcinoma. Peptides. 2017;88:62-6. doi: https://dx.doi.org/10.1016/j.peptides.2016.12.014.

157. Galicia-Garcia U, Benito-Vicente A, Jebari S, Larrea-Sebal A, Siddiqi H, Uribe KB, et al. Pathophysiology of type 2 diabetes mellitus. International Journal of Molecular Sciences. 2020;21(17):1-34. doi: https://dx.doi.org/10.3390/ijms21176275.

158. Gandolfi M, Smania N, Vella A, Picelli A, Chirumbolo S. Assessed and Emerging Biomarkers in Stroke and Training-Mediated Stroke Recovery: State of the Art. Neural Plasticity. 2017;2017:1389475. doi: https://dx.doi.org/10.1155/2017/1389475.

159. Gannon NP, Vaughan RA, Garcia-Smith R, Bisoffi M, Trujillo KA. Effects of the exercise-inducible myokine irisin on malignant and non-malignant breast epithelial cell behavior in vitro. International Journal of Cancer. 2015;136(4):E197-E202. doi: https://dx.doi.org/10.1002/ijc.29142.

160. Gannon NP, Vaughan RA, Garcia-Smith R, Bisoffi M, Trujillo KA. Effects of the exercise-inducible myokine irisin on malignant and non-malignant breast epithelial cell behavior in vitro. Int J Cancer. 2015;136(4):E197-202. Epub 2014/08/16. doi: 10.1002/ijc.29142. PubMed PMID: 25124080.

161. Gao Y, Yuan X, Zhu Z, Wang D, Liu Q, Gu W. Research and prospect of peptides for use in obesity treatment (review). Experimental and Therapeutic Medicine. 2020;20(1). doi: https://dx.doi.org/10.3892/ETM.2020.9364.

162. Gawlinska K, Gawlinski D, Przegalinski E, Filip M. Maternal high-fat diet during pregnancy and lactation provokes depressive-like behavior and influences the irisin/brain-derived neurotrophic factor axis and inflammatory factors in male and female offspring in rats. Journal of Physiology and Pharmacology. 2019;70(3):407-11. doi: https://dx.doi.org/10.26402/jpp.2019.3.07.

163. Gawlinska K, Gawlinski D, Przegalinski E, Filip M. Maternal high-fat diet during pregnancy and lactation provokes depressive-like behavior and influences the irisin/brain-derived neurotrophic factor axis and inflammatory factors in male and female offspring in rats. J Physiol Pharmacol. 2019;70(3). Epub 2019/09/21. doi: 10.26402/jpp.2019.3.07. PubMed PMID: 31539886.

164. Gerhard GS, Styer AM, Strodel WE, Roesch SL, Yavorek A, Carey DJ, et al. Gene expression profiling in subcutaneous, visceral and epigastric adipose tissues of patients with

extreme obesity. International Journal of Obesity. 2014;38(3):371-8. doi: https://dx.doi.org/10.1038/ijo.2013.152.

165. Gerhard GS, Styer AM, Strodel WE, Roesch SL, Yavorek A, Carey DJ, et al. Gene expression profiling in subcutaneous, visceral and epigastric adipose tissues of patients with extreme obesity. International journal of obesity (2005). 2014;38(3):371-8. Epub 2013/08/21. doi: 10.1038/ijo.2013.152. PubMed PMID: 23949615; PubMed Central PMCID: PMCPMC3925764.

166. Gharanei S, Shabir K, Brown JE, Weickert MO, Barber TM, Kyrou I, et al. Regulatory microRNAs in brown, brite and white adipose tissue. Cells. 2020;9(11):1-34. doi: https://dx.doi.org/10.3390/cells9112489.

167. Ghayomzadeh M, Earnest CP, Hackett D, SeyedAlinaghi S, Navalta JW, Gholami M, et al. Combination of resistance and aerobic exercise for six months improves bone mass and physical function in HIV infected individuals: A randomized controlled trial. Scandinavian journal of medicine & science in sports. 2021;31(3):720-32. doi: https://dx.doi.org/10.1111/sms.13871.

168. Ghazvini Zadegan F, Seyed Frotan F, Ghaedi K, Nasr Esfahani M. Fibronectin type III domain containing 5(FNDC5) gene expression level in colorectal cancer cell lines. European Journal of Human Genetics. 2019;27(Supplement 1):1014. doi: https://dx.doi.org/10.1038/s41431-019-0408-3.

169. Gholamnezhad Z, Safarian B, Esparham A, Mirzaei M, Esmaeilzadeh M, Boskabady MH. The modulatory effects of exercise on lipopolysaccharide-induced lung inflammation and injury: A systemic review. Life Sciences. 2022;293:120306. doi: https://dx.doi.org/10.1016/j.lfs.2022.120306.

170. Gholamnezhad Z, Safarian B, Esparham A, Mirzaei M, Esmaeilzadeh M, Boskabady MH. The modulatory effects of exercise on lipopolysaccharide-induced lung inflammation and injury: A systemic review. Life Sci. 2022;293:120306. Epub 2022/01/13. doi: 10.1016/j.lfs.2022.120306. PubMed PMID: 35016883.

171. Giardullo L, Corrado A, Maruotti N, Cici D, Mansueto N, Cantatore FP. Adipokine role in physiopathology of inflammatory and degenerative musculoskeletal diseases. International Journal of Immunopathology and Pharmacology. 2021;35. doi: https://dx.doi.org/10.1177/20587384211015034.

172. Goncalves RA, De Felice FG. The crosstalk between brain and periphery: Implications for brain health and disease. Neuropharmacology. 2021;197:108728. doi: https://dx.doi.org/10.1016/j.neuropharm.2021.108728.

173. Gonzalez-Gil AM, Elizondo-Montemayor L. The role of exercise in the interplay between myokines, hepatokines, osteokines, adipokines, and modulation of inflammation for energy substrate redistribution and fat mass loss: A review. Nutrients. 2020;12(6):1. doi: https://dx.doi.org/10.3390/nu12061899.

174. Gouveia MC, Vella JP, Cafeo FR, Affonso Fonseca FL, Bacci MR. Association between irisin and major chronic diseases: A review. European Review for Medical and Pharmacological Sciences. 2016;20(19):4072-7.

175. Gouveia MC, Vella JP, Cafeo FR, Affonso Fonseca FL, Bacci MR. Association between irisin and major chronic diseases: a review. Eur Rev Med Pharmacol Sci. 2016;20(19):4072-7. Epub 2016/10/25. PubMed PMID: 27775791.

176. Gries KJ, Zysik VS, Jobe TK, Griffin N, Leeds BP, Lowery JW. Muscle-derived factors influencing bone metabolism. Seminars in Cell and Developmental Biology. 2022;123:57-63. doi: https://dx.doi.org/10.1016/j.semcdb.2021.10.009.

177. Grzebisz-zatonska N, Poprzecki S, Pokora I, Mikolajec K, Kaminski T. Effect of seasonal variation during annual cyclist training on somatic function, white blood cells composition, immunological system, selected hormones and their interaction with irisin. Journal of Clinical Medicine. 2021;10(15):3299. doi: https://dx.doi.org/10.3390/jcm10153299.

178. Gul-Kahraman K, Yilmaz-Bozoglan M, Sahna E. Physiological and pharmacological effects of melatonin on remote ischemic perconditioning after myocardial ischemia-reperfusion injury in rats: Role of Cybb, Fas, NfkappaB, Irisin signaling pathway. Journal of Pineal Research. 2019;67(2):e12589. doi: https://dx.doi.org/10.1111/jpi.12589.

179. Guo M, Xiang L, Yao J, Zhang J, Zhu S, Wang D, et al. Comprehensive Transcriptome Profiling of NAFLD- and NASH-Induced Skeletal Muscle Dysfunction. Frontiers in Endocrinology. 2022;13:851520. doi: https://dx.doi.org/10.3389/fendo.2022.851520.

180. Guo P, Jin Z, Wang J, Sang A, Wu H. Irisin Rescues Blood-Brain Barrier Permeability following Traumatic Brain Injury and Contributes to the Neuroprotection of Exercise in Traumatic Brain Injury. Oxidative Medicine and Cellular Longevity. 2021;2021:1118981. doi: https://dx.doi.org/10.1155/2021/1118981.

181. Guo P, Liu L, Yang X, Li M, Zhao Q, Wu H. Irisin improves BBB dysfunction in SAP rats by inhibiting MMP-9 via the ERK/NF-kappaB signaling pathway. Cellular Signalling. 2022;93:110300. doi: https://dx.doi.org/10.1016/j.cellsig.2022.110300.

182. Guo P, Liu L, Yang X, Li M, Zhao Q, Wu H. Irisin improves BBB dysfunction in SAP rats by inhibiting MMP-9 via the ERK/NF-κB signaling pathway. Cell Signal. 2022;93:110300. Epub 2022/03/09. doi: 10.1016/j.cellsig.2022.110300. PubMed PMID: 35259454.

183. Hagood KL, Peterson J. Effect of ethanol diet on the circulating levels of myonectin and irisin. FASEB Journal. 2017;31(1 Supplement 1).

184. He W, Tang Y, Li C, Zhang X, Huang S, Tan B, et al. Exercise Enhanced Cardiac Function in Mice With Radiation-Induced Heart Disease via the FNDC5/Irisin-Dependent Mitochondrial Turnover Pathway. Frontiers in Physiology. 2021;12:739485. doi: https://dx.doi.org/10.3389/fphys.2021.739485.

185. He W, Tang Y, Li C, Zhang X, Huang S, Tan B, et al. Exercise Enhanced Cardiac Function in Mice With Radiation-Induced Heart Disease via the FNDC5/Irisin-Dependent Mitochondrial Turnover Pathway. Front Physiol. 2021;12:739485. Epub 2021/12/14. doi: 10.3389/fphys.2021.739485. PubMed PMID: 34899376; PubMed Central PMCID: PMCPMC8660102.

186. He Z, Li H, Zhou F, Han X, Chu L, Zhang S, et al. IRISIN attenuates osteoarthritis by inhibiting apoptosis of osteocytes through activating erk signaling pathway. Osteoarthritis and Cartilage. 2019;27(Supplement 1):S194. doi: https://dx.doi.org/10.1016/j.joca.2019.02.298.

187. He Z, Yu Z. Irisin attenuates osteoarthritis by inhibiting apoptosis of osteocyte through activating erk signaling pathway. Journal of Bone and Mineral Research. 2019;34(Supplement 1):71.

188. Hocking S, Samocha-Bonet D, Milner KL, Greenfield JR, Chisholm DJ. Adiposity and insulin resistance in humans: The role of the different tissue and cellular lipid depots. Endocrine Reviews. 2013;34(4):463-500. doi: https://dx.doi.org/10.1210/er.2012-1041.

189. Hou N, Du G, Han F, Zhang J, Jiao X, Sun X. Irisin Regulates Heme Oxygenase-1/Adiponectin Axis in Perivascular Adipose Tissue and Improves Endothelial Dysfunction in Diet-Induced Obese Mice. Cellular Physiology and Biochemistry. 2017;42(2):603-14. doi: https://dx.doi.org/10.1159/000477864.

190. Hou N, Liu Y, Han F, Wang D, Hou X, Hou S, et al. Irisin improves perivascular adipose tissue dysfunction via regulation of the heme oxygenase-1/adiponectin axis in diet-induced obese mice. Journal of Molecular and Cellular Cardiology. 2016;99:188-96. doi: https://dx.doi.org/10.1016/j.yjmcc.2016.09.005.

191. Hu C, Zhang X, Hu M, Teng T, Yuan YP, Song P, et al. Fibronectin type III domain-containing 5 improves aging-related cardiac dysfunction in mice. Aging Cell. 2022;21(3):e13556. doi: https://dx.doi.org/10.1111/acel.13556.

192. Huang CW, Chang YH, Lee HH, Wu JY, Huang JX, Chung YH, et al. Irisin, an exercise myokine, potently suppresses tumor proliferation, invasion, and growth in glioma. FASEB Journal. 2020;34(7):9678-93. doi: https://dx.doi.org/10.1096/fj.202000573RR.

193. Huang CW, Chang YH, Lee HH, Wu JY, Huang JX, Chung YH, et al. Irisin, an exercise myokine, potently suppresses tumor proliferation, invasion, and growth in glioma. FASEB journal : official publication of the Federation of American Societies for Experimental Biology. 2020;34(7):9678-93. Epub 2020/05/30. doi: 10.1096/fj.202000573RR. PubMed PMID: 32469121.

194. Huang J, Wang S, Xu F, Wang D, Yin H, Lai Q, et al. Exercise training with dietary restriction enhances circulating irisin level associated with increasing endothelial progenitor cell number in obese adults: An intervention study. PeerJ. 2017;2017(8):e3669. doi: https://dx.doi.org/10.7717/peerj.3669.

195. Huang Q, Kahn CR, Altindis E. Viral hormones: Expanding dimensions in endocrinology. Endocrinology. 2019;160(9):2165-79. doi: https://dx.doi.org/10.1210/en.2019-00271.

196. Huang Q, Wu M, Wu X, Zhang Y, Xia Y. Muscle-to-tumor crosstalk: The effect of exercise-induced myokine on cancer progression. Biochimica et Biophysica Acta - Reviews on Cancer. 2022;1877(5):188761. doi: https://dx.doi.org/10.1016/j.bbcan.2022.188761.

197. Huang Q, Wu M, Wu X, Zhang Y, Xia Y. Muscle-to-tumor crosstalk: The effect of exercise-induced myokine on cancer progression. Biochimica et biophysica acta Reviews on cancer. 2022;1877(5):188761. Epub 2022/07/20. doi: 10.1016/j.bbcan.2022.188761. PubMed PMID: 35850277.

198. Huang SH, Yang SM, Lo JJ, Wu SH, Tai MH. Irisin gene delivery ameliorates burn-induced sensory and motor neuropathy. International Journal of Molecular Sciences. 2020;21(20):1-17. doi: https://dx.doi.org/10.3390/ijms21207798.

199. Huang SH, Yang SM, Lo JJ, Wu SH, Tai MH. Irisin Gene Delivery Ameliorates Burn-Induced Sensory and Motor Neuropathy. Int J Mol Sci. 2020;21(20). Epub 2020/10/25. doi: 10.3390/ijms21207798. PubMed PMID: 33096842; PubMed Central PMCID: PMCPMC7589574.

200. Hunter P. Exercise in a bottle: Elucidating how exercise conveys health benefits might lead to new therapeutic options for a range of diseases from cancer to metabolic syndrome. EMBO Reports. 2016;17(2):136-8. doi: https://dx.doi.org/10.15252/embr.201541835.

201. Invernizzi M, de Sire A, Carda S, Venetis K, Reno F, Cisari C, et al. Bone Muscle Crosstalk in Spinal Cord Injuries: Pathophysiology and Implications for Patients' Quality of Life. Current Osteoporosis Reports. 2020;18(4):422-31. doi: https://dx.doi.org/10.1007/s11914-020-00601-7.

202. Iolascon G, Paoletta M, Liguori S, Curci C, Moretti A. Neuromuscular Diseases and Bone. Frontiers in Endocrinology. 2019;10:794. doi: https://dx.doi.org/10.3389/fendo.2019.00794.

203. Jerkic M, Leung CH, Khan Z, Mukkala AN, Ailenberg M, Beckett A, et al. Remote ischemic conditioning in posttraumatic hemorrhagic shock patients: Potential role of irisin. Shock. 2020;53(Supplement 1):122. doi: https://dx.doi.org/10.1097/SHK.0000000000001570.

204. Jiang S, Oh DS, Dorotea D, Son E, Kim DS, Ha H. Dojuksan ameliorates tubulointerstitial fibrosis through irisin-mediated muscle-kidney crosstalk. Phytomedicine. 2021;80:153393. doi: https://dx.doi.org/10.1016/j.phymed.2020.153393.

205. Jiang X, Hu Y, Zhou Y, Chen J, Sun C, Chen Z, et al. Irisin protects female mice with LPS-induced endometritis through the AMPK/NF-kappaB pathway. Iranian Journal of Basic Medical Sciences. 2021;24(9):1247-53. doi: https://dx.doi.org/10.22038/ijbms.2021.56781.12678.

206. Jiang X, Shen Z, Chen J, Wang C, Gao Z, Yu S, et al. Irisin Protects Against Motor Dysfunction of Rats with Spinal Cord Injury via Adenosine 5'-Monophosphate (AMP)-Activated Protein Kinase-Nuclear Factor Kappa-B Pathway. Frontiers in Pharmacology. 2020;11:582484. doi: https://dx.doi.org/10.3389/fphar.2020.582484.

207. Jiang X, Shen Z, Chen J, Wang C, Gao Z, Yu S, et al. Irisin Protects Against Motor Dysfunction of Rats with Spinal Cord Injury via Adenosine 5'-Monophosphate (AMP)-Activated Protein Kinase-Nuclear Factor Kappa-B Pathway. Front Pharmacol. 2020;11:582484. Epub 2020/12/15. doi: 10.3389/fphar.2020.582484. PubMed PMID: 33312127; PubMed Central PMCID: PMCPMC7701590.

208. Jin YH, Li ZY, Jiang XQ, Wu F, Li ZT, Chen H, et al. Irisin alleviates renal injury caused by sepsis via the NF-kappaB signaling pathway. European Review for Medical and Pharmacological Sciences. 2020;24(11):6470-6. doi: https://dx.doi.org/10.26355/eurrev_202006_21546.

209. Jin Z, Guo P, Li X, Ke J, Wang Y, Wu H. Neuroprotective effects of irisin against cerebral ischemia/ reperfusion injury via Notch signaling pathway. Biomedicine and Pharmacotherapy. 2019;120:109452. doi: https://dx.doi.org/10.1016/j.biopha.2019.109452.

210. Joro R, Korkmaz A, Lakka TA, Uusitalo ALT, Atalay M. Plasma irisin and its associations with oxidative stress in athletes suffering from overtraining syndrome. Physiology International. 2020;107(4):513-26. doi: https://dx.doi.org/10.1556/2060.2020.00037.

211. Jurimae J, Karvelyte V, Remmel L, Tamm AL, Purge P, Gruodyte-Raciene R, et al. Serum sclerostin concentration is associated with specific adipose, muscle and bone tissue markers in lean adolescent females with increased physical activity. Journal of Pediatric Endocrinology and Metabolism. 2021;34(6):755-61. doi: https://dx.doi.org/10.1515/jpem-2020-0662.

212. Jürimäe J, Karvelyte V, Remmel L, Tamm AL, Purge P, Gruodyte-Raciene R, et al. Serum sclerostin concentration is associated with specific adipose, muscle and bone tissue markers in lean adolescent females with increased physical activity. Journal of pediatric endocrinology & metabolism : JPEM. 2021;34(6):755-61. Epub 2021/04/15. doi: 10.1515/jpem-2020-0662. PubMed PMID: 33851796.

213. Jurimae J, Purge P. Irisin and inflammatory cytokines in elite male rowers: adaptation to volume-extended training period. The Journal of sports medicine and physical fitness. 2021;61(1):102-8. doi: https://dx.doi.org/10.23736/S0022-4707.20.11076-4.

214. Jürimäe J, Purge P. Irisin and inflammatory cytokines in elite male rowers: adaptation to volume-extended training period. J Sports Med Phys Fitness. 2021;61(1):102-8. Epub 2020/09/17. doi: 10.23736/s0022-4707.20.11076-4. PubMed PMID: 32936569.

215. Jurimae J, Purge P, Remmel L, Ereline J, Kums T, Kamandulis S, et al. Changes in irisin, inflammatory cytokines and aerobic capacity in response to three weeks of supervised sprint interval training in older men. The Journal of sports medicine and physical fitness. 2022. doi: https://dx.doi.org/10.23736/S0022-4707.22.13949-6.

216. Jürimäe J, Purge P, Remmel L, Ereline J, Kums T, Kamandulis S, et al. Changes in irisin, inflammatory cytokines and aerobic capacity in response to three weeks of supervised sprint interval training in older men. J Sports Med Phys Fitness. 2022. Epub 2022/06/11. doi: 10.23736/s0022-4707.22.13949-6. PubMed PMID: 35686866.

217. Jurimae J, Purge P, Tillmann V. Serum sclerostin and cytokine responses to prolonged sculling exercise in highly-trained male rowers. Journal of sports sciences. 2021;39(5):591-7. doi: https://dx.doi.org/10.1080/02640414.2020.1837428.

218. Jürimäe J, Purge P, Tillmann V. Serum sclerostin and cytokine responses to prolonged sculling exercise in highly-trained male rowers. J Sports Sci. 2021;39(5):591-7. Epub 2020/11/03. doi: 10.1080/02640414.2020.1837428. PubMed PMID: 33135583.

219. Kaneda H, Nakajima T, Haruyama A, Shibasaki I, Hasegawa T, Sawaguchi T, et al. Association of serum concentrations of irisin and the adipokines adiponectin and leptin with epicardial fat in cardiovascular surgery patients. PLoS ONE. 2018;13(8):e0201499. doi: https://dx.doi.org/10.1371/journal.pone.0201499.

220. Kang MJY, Vazquez G. A Pilot Study: An Open-Label Biomarker Development of Ketamine for Unipolar Refractory Depression. Biological Psychiatry. 2021;89(9 Supplement):S92-S3. doi: https://dx.doi.org/10.1016/j.biopsych.2021.02.243.

221. Karstoft K, Pedersen BK. Skeletal muscle as a gene regulatory endocrine organ. Current Opinion in Clinical Nutrition and Metabolic Care. 2016;19(4):270-5. doi: https://dx.doi.org/10.1097/MCO.0000000000000283.

222. Karstoft K, Pedersen BK. Skeletal muscle as a gene regulatory endocrine organ. Curr Opin Clin Nutr Metab Care. 2016;19(4):270-5. Epub 2016/04/22. doi: 10.1097/mco.0000000000000283. PubMed PMID: 27101470.

223. Kassouf T, Sumara G. Impact of conventional and atypical mapks on the development of metabolic diseases. Biomolecules. 2020;10(9):1-34. doi: https://dx.doi.org/10.3390/biom10091256.

224. Kawada T. Serum irisin concentration in patients with bladder cancer. International Urology and Nephrology. 2022;54(6):1245-6. doi: https://dx.doi.org/10.1007/s11255-022-03199-0.

225. Kawada T. Serum irisin concentration in patients with bladder cancer. Int Urol Nephrol. 2022;54(6):1245-6. Epub 2022/04/02. doi: 10.1007/s11255-022-03199-0. PubMed PMID: 35362818.

226. Kawao N, Kawaguchi M, Ohira T, Ehara H, Mizukami Y, Takafuji Y, et al. Renal failure suppresses muscle irisin expression, and irisin blunts cortical bone loss in mice. Journal of Cachexia, Sarcopenia and Muscle. 2022;13(1):758-71. doi: https://dx.doi.org/10.1002/jcsm.12892.

227. Kazimierczak-Kabzinska A, Marek B, Borgiel-Marek H, Kajdaniuk D, Kos-Kudla B. Assessing the blood concentration of new adipocytokines in patients with ischaemic stroke. Endokrynologia Polska. 2020;71(6):504-11. doi: https://dx.doi.org/10.5603/EP.A2020.0082.

228. Kepczynski L, Wcislo S, Korzeniewska-Dyl I, Polatynska K, Gach A, Moczulski D. No evidence for change in expression of TBC1D1 and TBC1D4 genes in cultured human adipocytes stimulated by myokines and adipokines. Adipocyte. 2021;10(1):153-9. doi: https://dx.doi.org/10.1080/21623945.2021.1900497.

229. Kępczyński Ł, Wcisło S, Korzeniewska-Dyl I, Połatyńska K, Gach A, Moczulski D. No evidence for change in expression of TBC1D1 and TBC1D4 genes in cultured human adipocytes stimulated by myokines and adipokines. Adipocyte. 2021;10(1):153-9. Epub 2021/03/27. doi: 10.1080/21623945.2021.1900497. PubMed PMID: 33769190; PubMed Central PMCID: PMCPMC8007147.

230. Kiess W, Kratzsch J, Sergeyev E, Neef M, Adler M, Pfaeffle R, et al. Metabolic syndrome in childhood and adolescence. Clinical Biochemistry. 2014;47(9):695. doi: https://dx.doi.org/10.1016/j.clinbiochem.2014.05.011.

231. Kim H, Lee DS, An TH, Park HJ, Kim WK, Bae KH, et al. Metabolic spectrum of liver failure in type 2 diabetes and obesity: From nafld to nash to hcc. International Journal of Molecular Sciences. 2021;22(9):4495. doi: https://dx.doi.org/10.3390/ijms22094495.

232. Kim JS, Galvao DA, Newton RU, Gray E, Taaffe DR. Exercise-induced myokines and their effect on prostate cancer. Nature Reviews Urology. 2021;18(9):519-42. doi: https://dx.doi.org/10.1038/s41585-021-00476-y.

233. Kim JS, Galvão DA, Newton RU, Gray E, Taaffe DR. Exercise-induced myokines and their effect on prostate cancer. Nature reviews Urology. 2021;18(9):519-42. Epub 2021/06/24. doi: 10.1038/s41585-021-00476-y. PubMed PMID: 34158658.

234. Kim MK, Ahn SK, Kim JH. Steady low intensity physical activity and healthy dietary habits are differently affect breast cancer progression. Breast. 2019;44(Supplement 1):S34-S5. doi: https://dx.doi.org/10.1016/S0960-9776%2819%2930158-4.

235. Kim OY, Chung JY, Song J. Effect of resveratrol on adipokines and myokines involved in fat browning: Perspectives in healthy weight against obesity. Pharmacological Research. 2019;148:104411. doi: https://dx.doi.org/10.1016/j.phrs.2019.104411.

236. Kim OY, Song J. The role of Irisin in Alzheimer's disease. Journal of Clinical Medicine. 2018;7(11):407. doi: https://dx.doi.org/10.3390/jcm7110407.

237. Kim SB, Heo JI, Kim H, Kim KS. Acetylation of PGC1alpha by Histone Deacetylase 1 Downregulation Is Implicated in Radiation-Induced Senescence of Brain Endothelial Cells. The journals of gerontology Series A, Biological sciences and medical sciences. 2019;74(6):787-93. doi: https://dx.doi.org/10.1093/gerona/gly167.

238. Kim SH, Kim JW, Hwang IG, Jang JS, Hong S, Kim TY, et al. Serum frailty biomarkers to predict overall survival in older patients with metastatic solid tumors: A substudy of prospective multi-center cohort study (KCSG PC13-09). Journal of Clinical Oncology. 2017;35(15 Supplement 1).

239. Kim SH, Kim JW, Hwang IG, Jang JS, Hong S, Kim TY, et al. Serum biomarkers for predicting overall survival and early mortality in older patients with metastatic solid tumors. Journal of Geriatric Oncology. 2019;10(5):749-56. doi: https://dx.doi.org/10.1016/j.jgo.2019.03.015.

240. Kim SH, Kim JW, Hwang IG, Jang JS, Hong S, Kim TY, et al. Serum biomarkers for predicting overall survival and early mortality in older patients with metastatic solid tumors. J Geriatr Oncol. 2019;10(5):749-56. Epub 2019/04/07. doi: 10.1016/j.jgo.2019.03.015. PubMed PMID: 30952517.

241. Kirk B, Feehan J, Lombardi G, Duque G. Muscle, Bone, and Fat Crosstalk: the Biological Role of Myokines, Osteokines, and Adipokines. Current Osteoporosis Reports. 2020;18(4):388-400. doi: https://dx.doi.org/10.1007/s11914-020-00599-y.

242. Ko FH, Huang FT. Study the Effect of Irisin on Prostate Cancer Cells. FASEB Journal. 2019;33(SUPPL 1):647.16. doi: https://dx.doi.org/10.1096/fasebj.2019.33.1_supplement.647.16.

243. Kong G, Jiang Y, Sun X, Cao Z, Zhang G, Zhao Z, et al. Irisin reverses the IL-6 induced epithelial-mesenchymal transition in osteosarcoma cell migration and invasion through the STAT3/Snail signaling pathway. Oncology Reports. 2017;38(5):2647-56. doi: https://dx.doi.org/10.3892/or.2017.5973.

244. Kong G, Jiang Y, Sun X, Cao Z, Zhang G, Zhao Z, et al. Irisin reverses the IL-6 induced epithelial-mesenchymal transition in osteosarcoma cell migration and invasion through the STAT3/Snail signaling pathway. Oncol Rep. 2017;38(5):2647-56. Epub 2017/10/20. doi: 10.3892/or.2017.5973. PubMed PMID: 29048621; PubMed Central PMCID: PMCPMC5780017.

245. Korta P, Pochec E, Mazur-Bialy A. Irisin as a Multifunctional Protein: Implications for Health and Certain Diseases. Medicina (Kaunas, Lithuania). 2019;55(8). doi: https://dx.doi.org/10.3390/medicina55080485.

246. Korta P, Pocheć E, Mazur-Biały A. Irisin as a Multifunctional Protein: Implications for Health and Certain Diseases. Medicina (Kaunas). 2019;55(8). Epub 2019/08/25. doi: 10.3390/medicina55080485. PubMed PMID: 31443222; PubMed Central PMCID: PMCPMC6722973.

247. Kothari C, Diorio C, Durocher F. The importance of breast adipose tissue in breast cancer. International Journal of Molecular Sciences. 2020;21(16):1-33. doi: https://dx.doi.org/10.3390/ijms21165760.

248. Kristof E, Klusoczki A, Veress R, Shaw A, Combi ZS, Varga K, et al. Interleukin-6 released from differentiating human beige adipocytes improves browning. Experimental Cell Research. 2019;377(1-2):47-55. doi: https://dx.doi.org/10.1016/j.yexcr.2019.02.015.

249. Kristóf E, Klusóczki Á, Veress R, Shaw A, Combi ZS, Varga K, et al. Interleukin-6 released from differentiating human beige adipocytes improves browning. Exp Cell Res. 2019;377(1-2):47-55. Epub 2019/02/23. doi: 10.1016/j.yexcr.2019.02.015. PubMed PMID: 30794803.

250. Kuchay MS, Martinez-Montoro JI, Kaur P, Fernandez-Garcia JC, Ramos-Molina B. Non-alcoholic fatty liver disease-related fibrosis and sarcopenia: An altered liver-muscle crosstalk leading to increased mortality risk. Ageing Research Reviews. 2022;80:101696. doi: https://dx.doi.org/10.1016/j.arr.2022.101696.

251. Kuloglu T, Artas G, Yardim M, Sahin I, Aydin Y, Beyoglu N, et al. Immunostaining characteristics of irisin in benign and malignant renal cancers. Biotechnic & histochemistry : official publication of the Biological Stain Commission. 2019;94(6):435-41. doi: https://dx.doi.org/10.1080/10520295.2019.1586998.

252. Kuloğlu T, Artaş G, Yardim M, Sahin I, Aydin Y, Beyoğlu N, et al. Immunostaining characteristics of irisin in benign and malignant renal cancers. Biotech Histochem. 2019;94(6):435-41. Epub 2019/03/22. doi: 10.1080/10520295.2019.1586998. PubMed PMID: 30896263.

253. Kuloglu T, Celik O, Aydin S, Hanifi Ozercan I, Acet M, Aydin Y, et al. Irisin immunostaining characteristics of breast and ovarian cancer cells. Cellular and molecular biology (Noisy-le-Grand, France). 2016;62(8):40-4. Epub 2016/08/23. PubMed PMID: 27545213.

254. Kuloglu T, Celik O, Aydin S, HanifiOzercan I, Acet M, Aydin Y, et al. Irisin immunostaining characteristics of breast and ovarian cancer cells. Cellular and Molecular Biology. 2016;62(8):40-4. doi: https://dx.doi.org/10.14715/cmb/2016.62.8.7.

255. Kuloglu T, Ozercan MR, Albayrak S, Aydin S, Bakal U, Yilmaz M, et al. Irisin immunohistochemistry in gastrointestinal system cancers. Biotechnic & histochemistry : official publication of the Biological Stain Commission. 2016;91(4):242-50. doi: https://dx.doi.org/10.3109/10520295.2015.1136988.

256. Kumar DP, Koka S, Li C, Rajagopal S. Inflammatory Mediators in Obesity. Mediators of Inflammation. 2019;2019:9481819. doi: https://dx.doi.org/10.1155/2019/9481819.

257. Kurrat A, Blei T, Kulling S, Mueller D, Soukup S, Weigt C, et al. Effects of lifelong dietary phytoestrogen exposure on the susceptibility to develop obesity-a study in ovariectomized and intact female wistar rats. Endocrine Reviews. 2014;35(SUPPL. 3).

258. Lage VKDS, de Paula FA, Lima LP, Santos JNV, dos Santos JM, Viegas AA, et al. Plasma levels of myokines and inflammatory markers are related with functional and respiratory performance in older adults with COPD and sarcopenia. Experimental Gerontology. 2022;164:111834. doi: https://dx.doi.org/10.1016/j.exger.2022.111834.

259. Lang X, Zhao N, He Q, Li X, Sun C, Zhang X. Treadmill exercise mitigates neuroinflammation and increases BDNF via activation of SIRT1 signaling in a mouse model of T2DM. Brain Research Bulletin. 2020;165:30-9. doi: https://dx.doi.org/10.1016/j.brainresbull.2020.09.015.

260. Lee HW, Ahmad M, Wang HW, Leenen FHH. Effects of exercise training on brain-derived neurotrophic factor in skeletal muscle and heart of rats post myocardial infarction. Experimental Physiology. 2017;102(3):314-28. doi: https://dx.doi.org/10.1113/EP086049.

261. Leiva M, Matesanz N, Pulgarin-Alfaro M, Nikolic I, Sabio G. Uncovering the Role of p38 Family Members in Adipose Tissue Physiology. Frontiers in Endocrinology. 2020;11:572089. doi: https://dx.doi.org/10.3389/fendo.2020.572089.

262. Li DJ, Li YH, Yuan HB, Qu LF, Wang P. The novel exercise-induced hormone irisin protects against neuronal injury via activation of the Akt and ERK1/2 signaling pathways and contributes to the neuroprotection of physical exercise in cerebral ischemia. Metabolism: Clinical and Experimental. 2017;68:31-42. doi: https://dx.doi.org/10.1016/j.metabol.2016.12.003.

263. Li DJ, Li YH, Yuan HB, Qu LF, Wang P. The novel exercise-induced hormone irisin protects against neuronal injury via activation of the Akt and ERK1/2 signaling pathways and contributes to the neuroprotection of physical exercise in cerebral ischemia. Metabolism. 2017;68:31-42. Epub 2017/02/12. doi: 10.1016/j.metabol.2016.12.003. PubMed PMID: 28183451.

264. Li F, Li Y, Duan Y, Hu CA, Tang Y, Yin Y. Myokines and adipokines: Involvement in the crosstalk between skeletal muscle and adipose tissue. Cytokine & growth factor reviews. 2017;33:73-82. Epub 2016/10/22. doi: 10.1016/j.cytogfr.2016.10.003. PubMed PMID: 27765498.

265. Li F, Li Y, Duan Y, Hu CAA, Tang Y, Yin Y. Myokines and adipokines: Involvement in the crosstalk between skeletal muscle and adipose tissue. Cytokine and Growth Factor Reviews. 2017;33:73-82. doi: https://dx.doi.org/10.1016/j.cytogfr.2016.10.003.

266. Li H, Wang F, Yang M, Sun J, Zhao Y, Tang D. The Effect of Irisin as a Metabolic Regulator and Its Therapeutic Potential for Obesity. International Journal of Endocrinology. 2021;2021:6572342. doi: https://dx.doi.org/10.1155/2021/6572342.

267. Li H, Zhang Y, Wang F, Donelan W, Zona MC, Li S, et al. Effects of irisin on the differentiation and browning of human visceral white adipocytes. American Journal of Translational Research. 2019;11(12):7410-21.

268. Li R, Wang X, Wu S, Wu Y, Chen H, Xin J, et al. Irisin ameliorates angiotensin II-induced cardiomyocyte apoptosis through autophagy. Journal of Cellular Physiology. 2019;234(10):17578-88. doi: https://dx.doi.org/10.1002/jcp.28382.

269. Li X, Cao X, Zhao M, Bao Y. The Changes of Irisin and Inflammatory Cytokines in the Age-Related Macular Degeneration and Retinal Vein Occlusion. Frontiers in Endocrinology. 2022;13:861757. doi: https://dx.doi.org/10.3389/fendo.2022.861757.

270. Li X, Jamal M, Guo P, Jin Z, Zheng F, Song X, et al. Irisin alleviates pulmonary epithelial barrier dysfunction in sepsis-induced acute lung injury via activation of AMPK/SIRT1 pathways. Biomedicine and Pharmacotherapy. 2019;118:109363. doi: https://dx.doi.org/10.1016/j.biopha.2019.109363.

271. Li X, Jamal M, Guo P, Jin Z, Zheng F, Song X, et al. Irisin alleviates pulmonary epithelial barrier dysfunction in sepsis-induced acute lung injury via activation of AMPK/SIRT1 pathways. Biomedicine & pharmacotherapy = Biomedecine & pharmacotherapie. 2019;118:109363. Epub 2019/09/24. doi: 10.1016/j.biopha.2019.109363. PubMed PMID: 31545277.

272. Li Y, Tan Q, Guo Y, Wang Q, Ding L, Li H, et al. The influence of exercise training on endothelial function, serum irisin and inflammatory markers in the elderly with metabolic syndrome. Clinical Laboratory. 2021;67(3):689-96. doi: https://dx.doi.org/10.7754/Clin.Lab.2020.200446.

273. Lin K, Sun X, Wang X, Wang H, Chen X. Circulating Adipokine Levels in Nonobese Women With Polycystic Ovary Syndrome and in Nonobese Control Women: A Systematic Review and Meta-Analysis. Frontiers in Endocrinology. 2020;11:537809. doi: https://dx.doi.org/10.3389/fendo.2020.537809.

274. Liu H, Wang M, Jin Z, Sun D, Zhu T, Liu X, et al. FNDC5 induces M2 macrophage polarization and promotes hepatocellular carcinoma cell growth by affecting the PPARgamma/NF-kappaB/NLRP3 pathway. Biochemical and Biophysical Research Communications. 2021;582:77-85. doi: https://dx.doi.org/10.1016/j.bbrc.2021.10.041.

275. Liu H, Zhao L, Wang M, Yang K, Jin Z, Zhao C, et al. FNDC5 Causes Resistance to Sorafenib by Activating the PI3K/Akt/Nrf2 Pathway in Hepatocellular Carcinoma Cells. Frontiers in Oncology. 2022;12:852095. doi: https://dx.doi.org/10.3389/fonc.2022.852095.

276. Liu J, Huang Y, Liu Y, Chen Y. Irisin enhances doxorubicin-induced cell apoptosis in pancreatic cancer by inhibiting the PI3K/Akt/NF-kB pathway. Medical Science Monitor. 2019;25:6085-96. doi: https://dx.doi.org/10.12659/MSM.917625.

277. Liu J, Huang Y, Liu Y, Chen Y. Irisin Enhances Doxorubicin-Induced Cell Apoptosis in Pancreatic Cancer by Inhibiting the PI3K/AKT/NF-κB Pathway. Medical science monitor : international medical journal of experimental and clinical research. 2019;25:6085-96. Epub 2019/08/15. doi: 10.12659/msm.917625. PubMed PMID: 31412018; PubMed Central PMCID: PMCPMC6705179.

278. Liu J, Song N, Huang Y, Chen Y. Irisin inhibits pancreatic cancer cell growth via the AMPK-mTOR pathway. Scientific reports. 2018;8(1):15247. doi: https://dx.doi.org/10.1038/s41598-018-33229-w.

279. Liu J, Song N, Huang Y, Chen Y. Irisin inhibits pancreatic cancer cell growth via the AMPK-mTOR pathway. Sci Rep. 2018;8(1):15247. Epub 2018/10/17. doi: 10.1038/s41598-018-33229-w. PubMed PMID: 30323244; PubMed Central PMCID: PMCPMC6189061.

280. Liu TY, Shi CX, Gao R, Sun HJ, Xiong XQ, Ding L, et al. Irisin inhibits hepatic gluconeogenesis and increases glycogen synthesis via the PI3K/Akt pathway in type 2 diabetic mice and hepatocytes. Clinical science (London, England : 1979). 2015;129(10):839-50. Epub 2015/07/23. doi: 10.1042/cs20150009. PubMed PMID: 26201094.

281. Liu Y, Fu Y, Liu Z, Shu S, Wang Y, Cai J, et al. Irisin is induced in renal ischemia-reperfusion to protect against tubular cell injury via suppressing p53. Biochimica et Biophysica Acta - Molecular Basis of Disease. 2020;1866(7):165792. doi: https://dx.doi.org/10.1016/j.bbadis.2020.165792.

282. Liu Y, Xu F, Jiang P. Effect of sitagliptin on expression of skeletal muscle peroxisome proliferator-activated receptor gamma coactivator-1alpha and irisin in a rat model of type 2 diabetes mellitus. Journal of International Medical Research. 2020;48(5). doi: https://dx.doi.org/10.1177/0300060519885569.

283. Liu Y, Xu F, Jiang P. Effect of sitagliptin on expression of skeletal muscle peroxisome proliferator-activated receptor γ coactivator-1α and irisin in a rat model of type 2 diabetes mellitus. The Journal of international medical research. 2020;48(5):300060519885569. Epub 2020/05/05. doi: 10.1177/0300060519885569. PubMed PMID: 32364035; PubMed Central PMCID: PMCPMC7218978.

284. Lombardi G, Ziemann E, Banfi G. Whole-body cryotherapy in athletes: From therapy to stimulation. An updated review of the literature. Frontiers in Physiology. 2017;8(MAY):258. doi: https://dx.doi.org/10.3389/fphys.2017.00258.

285. Lombardi G, Ziemann E, Banfi G, Corbetta S. Physical activity-dependent regulation of parathyroid hormone and calcium-phosphorous metabolism. International Journal of Molecular Sciences. 2020;21(15):1-50. doi: https://dx.doi.org/10.3390/ijms21155388.

286. Lopez-Cortes R, Gomez BB, Vazquez-Estevez S, Perez-Fentes D, Nunez C. Blood-based protein biomarkers in bladder urothelial tumors. Journal of Proteomics. 2021;247:104329. doi: https://dx.doi.org/10.1016/j.jprot.2021.104329.

287. Lu Y, Li G. Auricular acupuncture induces FNDC5/irisin and attenuates obese inflammation in mice. Acupuncture in medicine : journal of the British Medical Acupuncture Society. 2020;38(4):264-71. doi: https://dx.doi.org/10.1136/acupmed-2017-011405.

288. Lu Y, Li G. Auricular acupuncture induces FNDC5/irisin and attenuates obese inflammation in mice. Acupunct Med. 2020;38(4):264-71. Epub 2020/03/21. doi: 10.1136/acupmed-2017-011405. PubMed PMID: 32195595.

289. Luna-Ceron E, Huerta-Delgado AS, Roffe-Vazquez DN, Gonzalez-Gil AM, Villarreal-Calderon JR, Castillo EC, et al. Association of serum irisin levels with inflammatory, biochemical, and cardiac imaging parameters in patients with chronic heart failure versus controls. Circulation. 2021;144(SUPPL 1). doi: https://dx.doi.org/10.1161/circ.144.suppl-1.8956.

290. Ma C, Ding H, Deng Y, Liu H, Xiong X, Yang Y. Irisin: A New Code Uncover the Relationship of Skeletal Muscle and Cardiovascular Health During Exercise. Frontiers in Physiology. 2021;12:620608. doi: https://dx.doi.org/10.3389/fphys.2021.620608.

291. Ma C, Lin M, Gao J, Xu S, Huang L, Zhu J, et al. The impact of physical activity on blood inflammatory cytokines and neuroprotective factors in individuals with mild cognitive impairment: a systematic review and meta-analysis of randomized-controlled trials. Aging Clinical and Experimental Research. 2022;34(7):1471-84. doi: https://dx.doi.org/10.1007/s40520-021-02069-6.

292. Ma C, Lin M, Gao J, Xu S, Huang L, Zhu J, et al. The impact of physical activity on blood inflammatory cytokines and neuroprotective factors in individuals with mild cognitive impairment: a systematic review and meta-analysis of randomized-controlled trials. Aging Clin Exp Res. 2022;34(7):1471-84. Epub 2022/01/14. doi: 10.1007/s40520-021-02069-6. PubMed PMID: 35025094.

293. Ma J, Chen K. The role of Irisin in multiorgan protection. Molecular Biology Reports. 2021;48(1):763-72. doi: https://dx.doi.org/10.1007/s11033-020-06067-1.

294. Ma LY, Liu JM, Du GL, Dang XB. Irisin attenuates lipopolysaccharide-induced acute lung injury by downregulating inflammatory cytokine expression through miR-199a-mediated Rad23b overexpression. Experimental Cell Research. 2021;404(2):112593. doi: https://dx.doi.org/10.1016/j.yexcr.2021.112593.

295. Maalouf GE, El Khoury D. Exercise-Induced Irisin, the Fat Browning Myokine, as a Potential Anticancer Agent. Journal of Obesity. 2019;2019:6561726. doi: https://dx.doi.org/10.1155/2019/6561726.

296. Maalouf GE, El Khoury D. Exercise-Induced Irisin, the Fat Browning Myokine, as a Potential Anticancer Agent. J Obes. 2019;2019:6561726. Epub 2019/05/09. doi: 10.1155/2019/6561726. PubMed PMID: 31065382; PubMed Central PMCID: PMCPMC6466922.

297. Macedo APA, da Silva ASR, Munoz VR, Ropelle ER, Pauli JR. Mitochondrial dysfunction plays an essential role in remodeling aging adipose tissue. Mechanisms of Ageing and Development. 2021;200:111598. doi: https://dx.doi.org/10.1016/j.mad.2021.111598.

298. Manole E, Ceafalan LC, Popescu BO, Dumitru C, Bastian AE. Myokines as possible therapeutic targets in cancer cachexia. Journal of Immunology Research. 2018;2018:8260742. doi: https://dx.doi.org/10.1155/2018/8260742.

299. Manole E, Ceafalan LC, Popescu BO, Dumitru C, Bastian AE. Myokines as Possible Therapeutic Targets in Cancer Cachexia. J Immunol Res. 2018;2018:8260742. Epub 2018/11/15. doi: 10.1155/2018/8260742. PubMed PMID: 30426026; PubMed Central PMCID: PMCPMC6217752.

300. Martinez Gayo A, Felix Soriano E, Sainz N, Gonzalez Muniesa P, Moreno Aliaga M. Muscle mRNA expression changes promoted by DHA and/or aerobic exercise in aged obese female mice. Obesity Reviews. 2020;21(SUPPL 1). doi: https://dx.doi.org/10.1111/obr.13118.

301. Matsuo Y, Gleitsmann K, Mangner N, Werner S, Fischer T, Bowen TS, et al. Fibronectin type III domain containing 5 expression in skeletal muscle in chronic heart failure-

relevance of inflammatory cytokines. Journal of Cachexia, Sarcopenia and Muscle. 2015:62-72. doi: https://dx.doi.org/10.1002/jcsm.12006.

302. Matsuo Y, Gleitsmann K, Mangner N, Werner S, Fischer T, Bowen TS, et al. Fibronectin type III domain containing 5 expression in skeletal muscle in chronic heart failure-relevance of inflammatory cytokines. J Cachexia Sarcopenia Muscle. 2015;6(1):62-72. Epub 2015/07/03. doi: 10.1002/jcsm.12006. PubMed PMID: 26136413; PubMed Central PMCID: PMCPMC4435098.

303. Mazur-Bial AI, Bilski J, Wojcik D, Brzozowski B, Surmiak M, Hubalewska-Mazgaj M, et al. Beneficial effect of voluntary exercise on experimental colitis in mice fed a High-Fat diet: The role of irisin, adiponectin and proinflammatory biomarkers. Nutrients. 2017;9(4):410. doi: https://dx.doi.org/10.3390/nu9040410.

304. Mazur-Bialy AI. Superiority of the non-glycosylated form over the glycosylated form of irisin in the attenuation of adipocytic meta-inflammation: A potential factor in the fight against insulin resistance. Biomolecules. 2019;9(9):394. doi: https://dx.doi.org/10.3390/biom9090394.

305. Mazur-Bialy AI. Superiority of the Non-Glycosylated Form Over the Glycosylated Form of Irisin in the Attenuation of Adipocytic Meta-Inflammation: A Potential Factor in the Fight Against Insulin Resistance. Biomolecules. 2019;9(9). Epub 2019/08/24. doi: 10.3390/biom9090394. PubMed PMID: 31438646; PubMed Central PMCID: PMCPMC6770638.

306. Mazur-Bialy AI, Bilski J, Pochec E, Brzozowski T. New insight into the direct anti-inflammatory activity of a myokine irisin against proinflammatory activation of adipocytes. Implication for exercise in obesity. Journal of Physiology and Pharmacology. 2017;68(2):243-51.

307. Mazur-Bialy AI, Bilski J, Pochec E, Brzozowski T. New insight into the direct anti-inflammatory activity of a myokine irisin against proinflammatory activation of adipocytes. Implication for exercise in obesity. J Physiol Pharmacol. 2017;68(2):243-51. Epub 2017/06/15. PubMed PMID: 28614774.

308. Mazur-Bialy AI, Bilski J, Wojcik D, Brzozowski B, Surmiak M, Hubalewska-Mazgaj M, et al. Beneficial Effect of Voluntary Exercise on Experimental Colitis in Mice Fed a High-Fat Diet: The Role of Irisin, Adiponectin and Proinflammatory Biomarkers. Nutrients. 2017;9(4). Epub 2017/04/21. doi: 10.3390/nu9040410. PubMed PMID: 28425943; PubMed Central PMCID: PMCPMC5409749.

309. Mazur-Bialy AI, Oplawski M, Wypasek E, Zarawski M. Irisin-A newly discovered adipomiokine-Impairs growth and progression of breast cancer MDA-MB-231 cell line. Cytokine. 2015;76(1):107. doi: https://dx.doi.org/10.1016/j.cyto.2015.08.232.

310. Mazur-Bialy AI, Pochec E, Zarawski M. Anti-inflammatory properties of irisin, mediator of physical activity, are connected with TLR4/Myd88 signaling pathway activation. International Journal of Molecular Sciences. 2017;18(4):701. doi: https://dx.doi.org/10.3390/ijms18040701.

311. Mazur-Bialy AI, Pocheć E, Zarawski M. Anti-Inflammatory Properties of Irisin, Mediator of Physical Activity, Are Connected with TLR4/MyD88 Signaling Pathway Activation.

Int J Mol Sci. 2017;18(4). Epub 2017/03/28. doi: 10.3390/ijms18040701. PubMed PMID: 28346354; PubMed Central PMCID: PMCPMC5412287.

312. Medhat D, El-Bana MA, El-Daly SM, Ashour MN, Elias TR, Mohamed RA, et al. Influence of irisin on diet-induced metabolic syndrome in experimental rat model. Journal of Complementary and Integrative Medicine. 2021;18(2):347-54. doi: https://dx.doi.org/10.1515/jcim-2020-0030.

313. Men XM, Xu ZW, Tao X, Deng B, Qi KK. FNDC5 expression closely correlates with muscle fiber types in porcine longissimus dorsi muscle and regulates myosin heavy chains (MyHCs) mRNA expression in C2C12 cells. PeerJ. 2021;9:e11065. doi: https://dx.doi.org/10.7717/peerj.11065.

314. Menezes AC, Giori IG, Alexandre B, Andrade MS, Freitas FM, Machado MP, et al. Impact of exercise training and enalapril, either alone or in combination, on white adipose tissue renin-angiotensin system in a diet-induced obesity model. FASEB Journal. 2018;32(1 Supplement 1).

315. Metzger CE, Anand Narayanan S, Phan PH, Bloomfield SA. Hindlimb unloading causes regional loading-dependent changes in osteocyte inflammatory cytokines that are modulated by exogenous irisin treatment. NPJ microgravity. 2020;6:28. Epub 2020/10/22. doi: 10.1038/s41526-020-00118-4. PubMed PMID: 33083525; PubMed Central PMCID: PMCPMC7542171.

316. Metzger CE, Narayanan SA, Anderson AM, Zaweija DC, Bloomfield SA. Exogenous irisin treatment ameliorates inflammatory changes in osteocyte proteins and altered bone turnover in chronic DSS-induced inflammatory bowel disease. Journal of Bone and Mineral Research. 2018;33(Supplement 1):382.

317. Metzger CE, Narayanan SA, Elizondo JP, Carter AM, Zawieja DC, Hogan HA, et al. DSS-induced colitis produces inflammation-induced bone loss while irisin treatment mitigates the inflammatory state in both gut and bone. Scientific reports. 2019;9(1):15144. doi: https://dx.doi.org/10.1038/s41598-019-51550-w.

318. Metzger CE, Narayanan SA, Zawieja DC, Bloomfield SA. Exogenous administration of irisin during chronic TNBS-induced gut inflammation reverses inflammation-induced alterations in bone turnover. Gastroenterology. 2018;154(1 Supplement 1):S18.

319. Metzger CE, Narayanan SA, Zawieja DC, Bloomfield SA. Exogenous administration of irisin during chronic TNBS-induced gut inflammation reverses inflammation-induced alterations in bone turnover. Inflammatory Bowel Diseases. 2018;24(Supplement 1):S12-S3. doi: https://dx.doi.org/10.1093/ibd/izy019.

320. Micielska K, Gmiat A, Zychowska M, Kozlowska M, Walentukiewicz A, Lysak-Radomska A, et al. The beneficial effects of 15 units of high-intensity circuit training in women is modified by age, baseline insulin resistance and physical capacity. Diabetes Research and Clinical Practice. 2019;152:156-65. doi: https://dx.doi.org/10.1016/j.diabres.2019.05.009.

321. Miki D, Nakahara T, Murakami E, Abe-Chayama H, Fujino H, Ono A, et al. Association of FNDC5, PNPLA3 and TM6SF2 polymorphisms with hepatic steatosis in NAFLD patients. Hepatology v70 suppl1 2019. 2019;70(Supplement 1):1050A-1A.

322. Mohammadi I, Mahdavi AH, Rabiee F, Nasr Esfahani MH, Ghaedi K. Positive effects of conjugated linoleic acid (CLA) on the PGC1-alpha expression under the inflammatory conditions induced by TNF-alpha in the C2C12 cell line. Gene. 2020;735:144394. doi: https://dx.doi.org/10.1016/j.gene.2020.144394.

323. Molnar A, Szentpeteri A, Lorincz H, Seres I, Harangi M, Balogh Z, et al. Change of Fibroblast Growth Factor 21 Level Correlates with the Severity of Diabetic Sensory Polyneuropathy after Six-Week Physical Activity. Reviews in Cardiovascular Medicine. 2022;23(5):160. doi: https://dx.doi.org/10.31083/J.RCM2305160.

324. Moon HS, Mantzoros CS. Regulation of cell proliferation and malignant potential by irisin in endometrial, colon, thyroid and esophageal cancer cell lines. Metabolism: Clinical and Experimental. doi: https://dx.doi.org/10.1016/j.metabol.2013.10.005.

325. Moon HS, Mantzoros CS. Regulation of cell proliferation and malignant potential by irisin in endometrial, colon, thyroid and esophageal cancer cell lines. Metabolism: Clinical and Experimental. 2014;63(2):188-93. doi: https://dx.doi.org/10.1016/j.metabol.2013.10.005.

326. Moon HS, Mantzoros CS. Regulation of cell proliferation and malignant potential by irisin in endometrial, colon, thyroid and esophageal cancer cell lines. Metabolism. 2014;63(2):188-93. Epub 2013/11/26. doi: 10.1016/j.metabol.2013.10.005. PubMed PMID: 24268368.

327. Morell-Azanza L, Ojeda-Rodriguez A, Rendo-Urteaga T, Chueca M, Martinez JA, Azcona-Sanjulian MC, et al. Effect of 10-week weight loss treatment on Fibroblast Growth Factor 21 plasma levels in obese children. Obesity Facts. 2018;11(Supplement 1):222-3. doi: https://dx.doi.org/10.1159/000489691.

328. Moslemi E, Dehghan P, Khani M. The effect of date seed (Phoenix dactylifera) supplementation on inflammation, oxidative stress biomarkers, and performance in active people: A blinded randomized controlled trial protocol. Contemporary Clinical Trials Communications. 2022;28:100951. doi: https://dx.doi.org/10.1016/j.conctc.2022.100951.

329. Moslemi E, Dehghan P, Khani M. The effect of date seed (Phoenix dactylifera) supplementation on inflammation, oxidative stress biomarkers, and performance in active people: A blinded randomized controlled trial protocol. Contemp Clin Trials Commun. 2022;28:100951. Epub 2022/07/01. doi: 10.1016/j.conctc.2022.100951. PubMed PMID: 35769196; PubMed Central PMCID: PMCPMC9234073.

330. Moustafa A. Effect of Omega-3 or Omega-6 Dietary Supplementation on Testicular Steroidogenesis, Adipokine Network, Cytokines, and Oxidative Stress in Adult Male Rats. Oxidative Medicine and Cellular Longevity. 2021;2021:5570331. doi: https://dx.doi.org/10.1155/2021/5570331.

331. Moustafa A. Effect of Omega-3 or Omega-6 Dietary Supplementation on Testicular Steroidogenesis, Adipokine Network, Cytokines, and Oxidative Stress in Adult Male Rats. Oxid Med Cell Longev. 2021;2021:5570331. Epub 2021/07/15. doi: 10.1155/2021/5570331. PubMed PMID: 34257810; PubMed Central PMCID: PMCPMC8260291.

332. Nadermann N, Seward RK, Volkoff H. Effects of potential climate change -induced environmental modifications on food intake and the expression of appetite regulators in

goldfish. Comparative Biochemistry and Physiology -Part A : Molecular and Integrative Physiology. 2019;235:138-47. doi: https://dx.doi.org/10.1016/j.cbpa.2019.06.001.

333. Nadermann N, Volkoff H. Effects of short-term exercise on food intake and the expression of appetite-regulating factors in goldfish. Peptides. 2020;123:170182. doi: https://dx.doi.org/10.1016/j.peptides.2019.170182.

334. Namasivayam V, Pryseley AN, Wong WY, Wan WK, Lim KH, Azhar R, et al. Is the association between physical activity and colorectal adenoma mediated by muscle-derived myokines? United European Gastroenterology Journal. 2016;4(5 Supplement 1):A658-A9. doi: https://dx.doi.org/10.1177/2050640616663689.

335. Narayanan SA, Metzger CE, Bloomfield SA, Zawieja DC. Inflammation-associated colonic lymphatic architecture disruption is ameliorated by irisin a chronic rodent model of IBD. Gastroenterology. 2018;154(1 Supplement 1):S32-S3.

336. Narayanan SA, Metzger CE, Bloomfield SA, Zawieja DC. Inflammation-associated colonic lymphatic architecture disruption is ameliorated by irisin a chronic rodent model of IBD. Inflammatory Bowel Diseases. 2018;24(Supplement 1):S23. doi: https://dx.doi.org/10.1093/ibd/izy019.

337. Nejati M, Dehghan P, Khani M, Sarbakhsh P. The effect of Tribulus terrestris supplementation on inflammation, oxidative stress, and performance of recreational runners: study protocol for a randomized placebo-controlled trial. Trials. 2022;23(1):689. Epub 2022/08/20. doi: 10.1186/s13063-022-06630-0. PubMed PMID: 35986353.

338. Nowinska K, Jablonska K, Pawelczyk K, Piotrowska A, Partynska A, Gomulkiewicz A, et al. Expression of irisin/FNDC5 in cancer cells and stromal fibroblasts of non-small cell lung cancer. Cancers. 2019;11(10):1538. doi: https://dx.doi.org/10.3390/cancers11101538.

339. Nowinska K, Jablonska K, Pawelczyk K, Piotrowska A, Partynska A, Gomulkiewicz A, et al. Expression of Irisin/FNDC5 in Cancer Cells and Stromal Fibroblasts of Non-small Cell Lung Cancer. Cancers (Basel). 2019;11(10). Epub 2019/10/17. doi: 10.3390/cancers11101538. PubMed PMID: 31614634; PubMed Central PMCID: PMCPMC6826442.

340. Oflazoglu U, Caglar S, Yilmaz HE, Onal HT, Varol U, Salman T, et al. The relationship between sarcopenia detected in newly diagnosed colorectal cancer patients and FGF21, irisin and CRP levels. European Geriatric Medicine. 2022. doi: https://dx.doi.org/10.1007/s41999-022-00635-3.

341. Oflazoglu U, Caglar S, Yılmaz HE, Önal HT, Varol U, Salman T, et al. The relationship between sarcopenia detected in newly diagnosed colorectal cancer patients and FGF21, irisin and CRP levels. Eur Geriatr Med. 2022;13(4):795-803. Epub 2022/03/13. doi: 10.1007/s41999-022-00635-3. PubMed PMID: 35277853.

342. Ouchi N, Ohashi K, Shibata R, Murohara T. Protective roles of adipocytokines and myokines in cardiovascular disease. Circulation Journal. 2016;80(10):2073-80. doi: https://dx.doi.org/10.1253/circj.CJ-16-0663.

343. Ou-Yang WL, Guo B, Xu F, Lin X, Li FXZ, Shan SK, et al. The Controversial Role of Irisin in Clinical Management of Coronary Heart Disease. Frontiers in Endocrinology. 2021;12:678309. doi: https://dx.doi.org/10.3389/fendo.2021.678309.

344. Pafili K, Roden M. Nonalcoholic fatty liver disease (NAFLD) from pathogenesis to treatment concepts in humans. Molecular Metabolism. 2021;50:101122. doi: https://dx.doi.org/10.1016/j.molmet.2020.101122.

345. Panagiotou G, Mu L, Na B, Mukamal KJ, Mantzoros CS. Circulating irisin, omentin-1, and lipoprotein subparticles in adults at higher cardiovascular risk. Metabolism: Clinical and Experimental. 2014;63(10):1265-71. doi: https://dx.doi.org/10.1016/j.metabol.2014.06.001.

346. Panagiotou G, Mu L, Na B, Mukamal KJ, Mantzoros CS. Circulating irisin, omentin-1, and lipoprotein subparticles in adults at higher cardiovascular risk. Metabolism. 2014;63(10):1265-71. Epub 2014/07/26. doi: 10.1016/j.metabol.2014.06.001. PubMed PMID: 25060690; PubMed Central PMCID: PMCPMC4175146.

347. Panagiotou G, Pazaitou-Panayiotou K, Paschou SA, Komninou D, Kalogeris N, Vryonidou A, et al. Changes in Thyroid Hormone Levels Within the Normal and/or Subclinical Hyper- or Hypothyroid Range Do Not Affect Circulating Irisin Levels in Humans. Thyroid. 2016;26(8):1039-45. doi: https://dx.doi.org/10.1089/thy.2016.0098.

348. Panagiotou G, Pazaitou-Panayiotou K, Paschou SA, Komninou D, Kalogeris N, Vryonidou A, et al. Changes in Thyroid Hormone Levels Within the Normal and/or Subclinical Hyper- or Hypothyroid Range Do Not Affect Circulating Irisin Levels in Humans. Thyroid. 2016;26(8):1039-45. Epub 2016/06/09. doi: 10.1089/thy.2016.0098. PubMed PMID: 27267080.

349. Panagiotou G, Triantafyllidou S, Tarlatzis BC, Papakonstantinou E. Serum Levels of Irisin and Omentin-1 in Breast Neoplasms and Their Association with Tumor Histology. International Journal of Endocrinology. 2021;2021:6656671. doi: https://dx.doi.org/10.1155/2021/6656671.

350. Panagiotou G, Triantafyllidou S, Tarlatzis BC, Papakonstantinou E. Serum Levels of Irisin and Omentin-1 in Breast Neoplasms and Their Association with Tumor Histology. Int J Endocrinol. 2021;2021:6656671. Epub 2021/03/11. doi: 10.1155/2021/6656671. PubMed PMID: 33688343; PubMed Central PMCID: PMCPMC7920698 in the Breast Surgery Department, Genesis Clinic, Thessaloniki, Greece.

351. Papadopetraki A, Maridaki M, Zagouri F, Dimopoulos, Koutsilieris M, Philippou A. Physical Exercise Restrains Cancer Progression through Muscle-Derived Factors. Cancers. 2022;14(8):1892. doi: https://dx.doi.org/10.3390/cancers14081892.

352. Park EJ, Myint PK, Ito A, Appiah MG, Darkwah S, Kawamoto E, et al. Integrin-Ligand Interactions in Inflammation, Cancer, and Metabolic Disease: Insights Into the Multifaceted Roles of an Emerging Ligand Irisin. Frontiers in Cell and Developmental Biology. 2020;8:588066. doi: https://dx.doi.org/10.3389/fcell.2020.588066.

353. Park EJ, Myint PK, Ito A, Appiah MG, Darkwah S, Kawamoto E, et al. Integrin-Ligand Interactions in Inflammation, Cancer, and Metabolic Disease: Insights Into the Multifaceted Roles of an Emerging Ligand Irisin. Front Cell Dev Biol. 2020;8:588066. Epub 2020/11/17. doi: 10.3389/fcell.2020.588066. PubMed PMID: 33195249; PubMed Central PMCID: PMCPMC7649757.

354. Park J, Bae J, Lee J. Complex Exercise Improves Anti-Inflammatory and Anabolic Effects in Osteoarthritis-Induced Sarcopenia in Elderly Women. Healthcare (Basel,

Switzerland). 2021;9(6). Epub 2021/07/03. doi: 10.3390/healthcare9060711. PubMed PMID: 34200794; PubMed Central PMCID: PMCPMC8230475.

355. Park MJ, Kim DI, Choi JH, Heo YR, Park SH. New role of irisin in hepatocytes: The protective effect of hepatic steatosis in vitro. Cellular Signalling. 2015;27(9):1831-9. doi: https://dx.doi.org/10.1016/j.cellsig.2015.04.010.

356. Paskeh MDA, Asadi A, Mirzaei S, Hashemi M, Entezari M, Raesi R, et al. Targeting AMPK signaling in ischemic/reperfusion injury: From molecular mechanism to pharmacological interventions. Cellular Signalling. 2022;94:110323. doi: https://dx.doi.org/10.1016/j.cellsig.2022.110323.

357. Patoulias D, Stavropoulos K, Imprialos K, Athyros V, Grassos H, Doumas M, et al. Inflammatory markers in cardiovascular disease; lessons learned and future perspectives. Current Vascular Pharmacology. 2021;19(3):323-42. doi: https://dx.doi.org/10.2174/1570161118666200318104434.

358. Paulo E, Wang B. Towards a better understanding of beige adipocyte plasticity. Cells. 2019;8(12):1552. doi: https://dx.doi.org/10.3390/cells8121552.

359. Pazgan-Simon M, Kukla M, Simon K, Lekstan A, Gabrek E, Zuwala-Jagiello J. Serum irisin level a new possible marker of HCC progression. Clinical and Experimental Hepatology. 2018;4(2):139.

360. Pazgan-Simon M, Zuwala-Jagiello J, Kukla M, Grzebyk E, Simon K. Serum concentrations of selected adipokines in virus-related liver cirrhosis and hepatocellular carcinoma. Clinical and Experimental Hepatology. 2020;6(3):235-42. doi: https://dx.doi.org/10.5114/ceh.2020.99517.

361. Pazgan-Simon M, Zuwała-Jagiełło J, Kukla M, Grzebyk E, Simon K. Serum concentrations of selected adipokines in virus-related liver cirrhosis and hepatocellular carcinoma. Clin Exp Hepatol. 2020;6(3):235-42. Epub 2020/11/05. doi: 10.5114/ceh.2020.99517. PubMed PMID: 33145430; PubMed Central PMCID: PMCPMC7592085.

362. Pazgan-Simon M, Zuwala-Jagiello J, Menzyk T, Bator M, Derra A, Lekstan A, et al. Serum betatrophin and irisin levels in hepatocellular carcinoma. Journal of physiology and pharmacology : an official journal of the Polish Physiological Society. 2020;71(1). doi: https://dx.doi.org/10.26402/jpp.2020.1.11.

363. Pazgan-Simon M, Zuwala-Jagiello J, Menzyk T, Bator M, Derra A, Lekstan A, et al. Serum betatrophin and irisin levels in hepatocellular carcinoma. J Physiol Pharmacol. 2020;71(1). Epub 2020/06/20. doi: 10.26402/jpp.2020.1.11. PubMed PMID: 32554846.

364. Pedersen BK, Febbraio MA. Muscles, exercise and obesity: Skeletal muscle as a secretory organ. Nature Reviews Endocrinology. 2012;8(8):457-65. doi: https://dx.doi.org/10.1038/nrendo.2012.49.

365. Pedersen BK, Febbraio MA. Muscles, exercise and obesity: skeletal muscle as a secretory organ. Nature reviews Endocrinology. 2012;8(8):457-65. Epub 2012/04/05. doi: 10.1038/nrendo.2012.49. PubMed PMID: 22473333.

366. Peradze N, Farr OM, Mantzoros CS. Research developments in metabolism 2018. Metabolism: Clinical and Experimental. 2019;91:70-9. doi: https://dx.doi.org/10.1016/j.metabol.2018.11.011.

367. Perakakis N, Arend L, Potasso L, Lamprinou A, Polyzou E, Kassanos A, et al. Effects of the gestational diabetes-risk polymorphism rs10501320 of MADD gene on metabolic phenotype and glucose homeostasis in women with history of gestational diabetes. Diabetologie und Stoffwechsel. 2015;10(Supplement 1). doi: https://dx.doi.org/10.1055/s-0035-1549700.

368. Pin F, Bonewald LF, Bonetto A. Role of myokines and osteokines in cancer cachexia. Experimental Biology and Medicine. 2021;246(19):2118-27. doi: https://dx.doi.org/10.1177/15353702211009213.

369. Pin F, Bonewald LF, Bonetto A. Role of myokines and osteokines in cancer cachexia. Experimental biology and medicine (Maywood, NJ). 2021;246(19):2118-27. Epub 2021/04/27. doi: 10.1177/15353702211009213. PubMed PMID: 33899538; PubMed Central PMCID: PMCPMC8524772.

370. Pinkowska A, Nowinska K, Ciesielska U, Podhorska-Okolow M. Irisin Association with Ki-67, MCM3 and MT-I/II in Squamous Cell Carcinomas of the Larynx. Biomolecules. 2021;12(1). Epub 2022/01/22. doi: 10.3390/biom12010052. PubMed PMID: 35053200; PubMed Central PMCID: PMCPMC8774284.

371. Pinkowska A, Nowinska K, Ciesielska U, Podhorska-Okolow M. Irisin association with ki-67, mcm3 and mt-i/ii in squamous cell carcinomas of the larynx. Biomolecules. 2022;12(1):52. doi: https://dx.doi.org/10.3390/biom12010052.

372. Pinkowska A, Podhorska-Okolow M, Dziegiel P, Nowinska K. The role of irisin in cancer disease. Cells. 2021;10(6):1479. doi: https://dx.doi.org/10.3390/cells10061479.

373. Pinkowska A, Podhorska-Okołów M, Dzięgiel P, Nowińska K. The Role of Irisin in Cancer Disease. Cells. 2021;10(6). Epub 2021/07/03. doi: 10.3390/cells10061479. PubMed PMID: 34204674; PubMed Central PMCID: PMCPMC8231117.

374. Polyzos SA, Mousiolis A, Mintziori G, Goulis DG. Nonalcoholic fatty liver disease in males with low testosterone concentrations. Diabetes and Metabolic Syndrome: Clinical Research and Reviews. 2020;14(5):1571-7. doi: https://dx.doi.org/10.1016/j.dsx.2020.07.049.

375. Provatopoulou X, Georgiou GP, Kalogera E, Kalles V, Matiatou MA, Papapanagiotou I, et al. Serum irisin levels are lower in patients with breast cancer: Association with disease diagnosis and tumor characteristics. BMC Cancer. 2015;15(1):898. doi: https://dx.doi.org/10.1186/s12885-015-1898-1.

376. Provatopoulou X, Georgiou GP, Kalogera E, Kalles V, Matiatou MA, Papapanagiotou I, et al. Serum irisin levels are lower in patients with breast cancer: association with disease diagnosis and tumor characteristics. BMC Cancer. 2015;15:898. Epub 2015/11/13. doi: 10.1186/s12885-015-1898-1. PubMed PMID: 26560078; PubMed Central PMCID: PMCPMC4642638.

377. Ptak T, Younes Z, Shafran I, Lee S, Miner PB, Kayhan C, et al. Long-term safety and efficacy of certolizumab pegol therapy in patients with moderate to severe crohn's disease: 5-

year results. American Journal of Gastroenterology. 2015;110(Supplement 1):S774-S5. doi: https://dx.doi.org/10.14309/00000434-201510001-01821.

378. Raafat Ibrahim R, Shafik NM, El-Esawy RO, El-Sakaa MH, Arakeeb HM, El-Sharaby RM, et al. The emerging role of irisin in experimentally induced arthritis: a recent update involving HMGB1/MCP1/Chitotriosidase I-mediated necroptosis. Redox Report. 2022;27(1):21-31. doi: https://dx.doi.org/10.1080/13510002.2022.2031516.

379. Raafat Ibrahim R, Shafik NM, El-Esawy RO, El-Sakaa MH, Arakeeb HM, El-Sharaby RM, et al. The emerging role of irisin in experimentally induced arthritis: a recent update involving HMGB1/MCP1/Chitotriosidase I-mediated necroptosis. Redox report : communications in free radical research. 2022;27(1):21-31. Epub 2022/02/01. doi: 10.1080/13510002.2022.2031516. PubMed PMID: 35094663; PubMed Central PMCID: PMCPMC8803109.

380. Rabiee F, Lachinani L, Ghaedi S, Nasr-Esfahani MH, Megraw TL, Ghaedi K. New insights into the cellular activities of Fndc5/Irisin and its signaling pathways. Cell and Bioscience. 2020;10(1):51. doi: https://dx.doi.org/10.1186/s13578-020-00413-3.

381. Rajbi H, Gharakhanlou R, Khalighfard S, Setoudeh V, Alizadeh A. The effect of 8 weeks of interval aerobic exercise before and after induction of breast cancer on serum level of irisin and tumor growth in balb/c mice. Journal of Isfahan Medical School. 2018;35(459):1775-84.

382. Reinehr T, Roth CL. Inflammation Markers in Type 2 Diabetes and the Metabolic Syndrome in the Pediatric Population. Current Diabetes Reports. 2018;18(12):131. doi: https://dx.doi.org/10.1007/s11892-018-1110-5.

383. Reinehr T, Roth CL. Inflammation Markers in Type 2 Diabetes and the Metabolic Syndrome in the Pediatric Population. Curr Diab Rep. 2018;18(12):131. Epub 2018/10/20. doi: 10.1007/s11892-018-1110-5. PubMed PMID: 30338401.

384. Ren Y, Qiu M, Zhang J, Bi J, Wang M, Hu L, et al. Low Serum Irisin Concentration Is Associated with Poor Outcomes in Patients with Acute Pancreatitis, and Irisin Administration Protects Against Experimental Acute Pancreatitis. Antioxidants and Redox Signaling. 2019;31(11):771-85. doi: https://dx.doi.org/10.1089/ars.2019.7731.

385. Ren YF, Wang MZ, Bi JB, Zhang J, Zhang L, Liu WM, et al. Irisin attenuates intestinal injury, oxidative and endoplasmic reticulum stress in mice with L-arginine-induced acute pancreatitis. World Journal of Gastroenterology. 2019;25(45):6653-67. doi: https://dx.doi.org/10.3748/wjg.v25.i45.6653.

386. Ren YF, Wang MZ, Bi JB, Zhang J, Zhang L, Liu WM, et al. Irisin attenuates intestinal injury, oxidative and endoplasmic reticulum stress in mice with L-arginine-induced acute pancreatitis. World J Gastroenterol. 2019;25(45):6653-67. Epub 2019/12/14. doi: 10.3748/wjg.v25.i45.6653. PubMed PMID: 31832004; PubMed Central PMCID: PMCPMC6906211.

387. Rifai N, Watson ID, Miller WG. Commercial immunoassays in biomarkers studies: Researchers beware! Clinical Chemistry. 2012;58(10):1387-8. doi: https://dx.doi.org/10.1373/clinchem.2012.192351.

388. Rizk FH, Soliman NA, Heabah NA, Abdel Ghafar MT, El-Attar SH, Elsaadany A. Fenofibrate Improves Cognitive Impairment Induced by High-Fat High-Fructose Diet: A Possible Role of Irisin and Heat Shock Proteins. ACS Chemical Neuroscience. 2022;13(12):1782-9. doi: https://dx.doi.org/10.1021/acschemneuro.2c00186.

389. Rizk FH, Soliman NA, Heabah NA, Abdel Ghafar MT, El-Attar SH, Elsaadany A. Fenofibrate Improves Cognitive Impairment Induced by High-Fat High-Fructose Diet: A Possible Role of Irisin and Heat Shock Proteins. ACS Chem Neurosci. 2022;13(12):1782-9. Epub 2022/06/03. doi: 10.1021/acschemneuro.2c00186. PubMed PMID: 35652596.

390. Rodriguez A, Fernandez Quintana B, Paniagua M, Hernandez Pardos AW, Valenti V, Moncada R, et al. Fibronectin type III domain-containing 4 drives fat browning and inhibits lipogenesis in human omental adipocytes. Obesity Facts. 2021;14(SUPPL 1):22-3. doi: https://dx.doi.org/10.1159/000515911.

391. Rosa TS, Neves RVP, Deus LA, Sousa CV, da Silva Aguiar S, de Souza MK, et al. Sprint and endurance training in relation to redox balance, inflammatory status and biomarkers of aging in master athletes. Nitric Oxide - Biology and Chemistry. 2020;102:42-51. doi: https://dx.doi.org/10.1016/j.niox.2020.05.004.

392. Roth CL, Molica F, Kwak BR. Browning of white adipose tissue as a therapeutic tool in the fight against atherosclerosis. Metabolites. 2021;11(5):319. doi: https://dx.doi.org/10.3390/metabo11050319.

393. Saad FA. Novel insights into the complex architecture of osteoporosis molecular genetics. Annals of the New York Academy of Sciences. 2020;1462(1):37-52. doi: https://dx.doi.org/10.1111/nyas.14231.

394. Sadim M, Xu Y, Selig K, Paulus J, Uthe R, Agarwl S, et al. A prospective evaluation of clinical and genetic predictors of weight changes in breast cancer survivors. Cancer. 2017;123(13):2413-21. doi: https://dx.doi.org/10.1002/cncr.30628.

395. Saeedi Sadr A, Ehteram H, Seyed Hosseini E, Alizadeh Zarei M, Alizadeh Bafrani H, Haddad Kashani H. The Effect of Irisin on Proliferation, Apoptosis, and Expression of Metastasis Markers in Prostate Cancer Cell Lines. Oncology and Therapy. 2022. doi: https://dx.doi.org/10.1007/s40487-022-00194-4.

396. Saeedi Sadr A, Ehteram H, Seyed Hosseini E, Alizadeh Zarei M, Hassani Bafrani H, Haddad Kashani H. Correction to: The Effect of Irisin on Proliferation, Apoptosis, and Expression of Metastasis Markers in Prostate Cancer Cell Lines (Oncology and Therapy, (2022), 10.1007/s40487-022-00194-4). Oncology and Therapy. 2022. doi: https://dx.doi.org/10.1007/s40487-022-00199-z.

397. Saeedi Sadr A, Ehteram H, Seyed Hosseini E, Alizadeh Zarei M, Hassani Bafrani H, Haddad Kashani H. The Effect of Irisin on Proliferation, Apoptosis, and Expression of Metastasis Markers in Prostate Cancer Cell Lines. Oncol Ther. 2022. Epub 2022/04/26. doi: 10.1007/s40487-022-00194-4. PubMed PMID: 35467303.

398. Saeedi Sadr A, Ehteram H, Seyed Hosseini E, Alizadeh Zarei M, Hassani Bafrani H, Haddad Kashani H. Correction to: The Effect of Irisin on Proliferation, Apoptosis, and Expression of Metastasis Markers in Prostate Cancer Cell Lines. Oncol Ther. 2022. Epub 2022/05/14. doi: 10.1007/s40487-022-00199-z. PubMed PMID: 35556237.

399. Saidi O, Rochette E, Dore E, Maso F, Raoux J, Andrieux F, et al. Randomized double-blind controlled trial on the effect of proteins with different tryptophan/large neutral amino acid ratios on sleep in adolescents: The protmorpheus study. Nutrients. 2020;12(6):1-17. doi: https://dx.doi.org/10.3390/nu12061885.

400. Sanchis-Gomar F, Perez-Quilis C. Irisinemia: A novel concept to coin in clinical medicine? Annals of Nutrition and Metabolism. 2013;63(1-2):60-1. doi: https://dx.doi.org/10.1159/000354090.

401. Sanderson M, McKinlay BJ, Theocharidis A, Kouvelioti R, Falk B, Klentrou P. Changes in Inflammatory Cytokines and Irisin in Response to High Intensity Swimming in Adolescent versus Adult Male Swimmers. Sports (Basel, Switzerland). 2020;8(12). Epub 2020/12/05. doi: 10.3390/sports8120157. PubMed PMID: 33271764; PubMed Central PMCID: PMCPMC7760547.

402. Saraev GB, Mironova ES, Linkova NS, Bunin VA, Paltsev MA, Kvetnoy IM. Investigation of signal molecules in saliva: prospects of application for diagnostics of myocardial infarction and the aging rate of different age people. Advances in gerontology = Uspekhi gerontologii. 2019;32(3):364-9.

403. Schaalan MF, Ramadan BK, Abd Elwahab AH. Synergistic effect of carnosine on browning of adipose tissue in exercised obese rats; a focus on circulating irisin levels. Journal of Cellular Physiology. 2018;233(6):5044-57. doi: https://dx.doi.org/10.1002/jcp.26370.

404. Schulze-Tanzil G. Experimental therapeutics for the treatment of osteoarthritis. Journal of Experimental Pharmacology. 2021;13:101-25. doi: https://dx.doi.org/10.2147/JEP.S237479.

405. Seifert M, Noczynska A, Wikiera B. Irisin in the growth hormone deficient children: before and on rhGH therapy. Hormone Research in Paediatrics. 2021;94(SUPPL 1):164. doi: https://dx.doi.org/10.1159/000518849.

406. Seo DY, Lee SR, Heo JW, No MH, Rhee BD, Ko KS, et al. Ursolic acid in health and disease. Korean Journal of Physiology and Pharmacology. 2018;22(3):235-48. doi: https://dx.doi.org/10.4196/kjpp.2018.22.3.235.

407. Seo DY, Lee SR, Heo JW, No MH, Rhee BD, Ko KS, et al. Ursolic acid in health and disease. The Korean journal of physiology & pharmacology : official journal of the Korean Physiological Society and the Korean Society of Pharmacology. 2018;22(3):235-48. Epub 2018/05/03. doi: 10.4196/kjpp.2018.22.3.235. PubMed PMID: 29719446; PubMed Central PMCID: PMCPMC5928337.

408. Shahidi S, Hejazi J, Moghimi M, Borji S, Zabihian S, Fathi M. Circulating Irisin Levels and Redox Status Markers in Patients with Gastric Cancer: A Case-Control Study. Asian Pacific journal of cancer prevention : APJCP. 2020;21(10):2847-51. doi: https://dx.doi.org/10.31557/APJCP.2020.21.10.2847.

409. Shahidi S, Hejazi J, Moghimi M, Borji S, Zabihian S, Fathi M. Circulating Irisin Levels and Redox Status Markers in Patients with Gastric Cancer: A Case-Control Study. Asian Pac J Cancer Prev. 2020;21(10):2847-51. Epub 2020/10/29. doi: 10.31557/apjcp.2020.21.10.2847. PubMed PMID: 33112539; PubMed Central PMCID: PMCPMC7798161.

410. Shao L, Chen J, Song H, Zhang Y, Wu F, Wang W, et al. Irisin suppresses the migration, proliferation, and invasion of lung cancer cells via inhibition of epithelial-to-mesenchymal transition. Biochemical and Biophysical Research Communications. 2016. doi: https://dx.doi.org/10.1016/j.bbrc.2016.12.084.

411. Shao L, Chen J, Song H, Zhang Y, Wu F, Wang W, et al. Irisin suppresses the migration, proliferation, and invasion of lung cancer cells via inhibition of epithelial-to-mesenchymal transition. Biochemical and Biophysical Research Communications. 2017;485(3):598-605. doi: https://dx.doi.org/10.1016/j.bbrc.2016.12.084.

412. Shao L, Li H, Chen J, Song H, Zhang Y, Wu F, et al. Irisin suppresses the migration, proliferation, and invasion of lung cancer cells via inhibition of epithelial-to-mesenchymal transition. Biochem Biophys Res Commun. 2017;485(3):598-605. Epub 2016/12/18. doi: 10.1016/j.bbrc.2016.12.084. PubMed PMID: 27986567.

413. Shao L, Meng D, Yang F, Song H, Tang D. Irisin-mediated protective effect on LPS-induced acute lung injury via suppressing inflammation and apoptosis of alveolar epithelial cells. Biochemical and Biophysical Research Communications. 2017;487(2):194-200. doi: https://dx.doi.org/10.1016/j.bbrc.2017.04.020.

414. Shen HH, Huang SY, Kung CW, Chen SY, Chen YF, Cheng PY, et al. Genistein ameliorated obesity accompanied with adipose tissue browning and attenuation of hepatic lipogenesis in ovariectomized rats with high-fat diet. Journal of Nutritional Biochemistry. 2019;67:111-22. doi: https://dx.doi.org/10.1016/j.jnutbio.2019.02.001.

415. Shi G, Tang N, Qiu J, Zhang D, Huang F, Cheng Y, et al. Irisin stimulates cell proliferation and invasion by targeting the PI3K/AKT pathway in human hepatocellular carcinoma. Biochemical and Biophysical Research Communications. 2017;493(1):585-91. doi: https://dx.doi.org/10.1016/j.bbrc.2017.08.148.

416. Shi G, Tang N, Qiu J, Zhang D, Huang F, Cheng Y, et al. Irisin stimulates cell proliferation and invasion by targeting the PI3K/AKT pathway in human hepatocellular carcinoma. Biochem Biophys Res Commun. 2017;493(1):585-91. Epub 2017/09/05. doi: 10.1016/j.bbrc.2017.08.148. PubMed PMID: 28867187.

417. Shi J, Fan J, Su Q, Yang Z. Cytokines and Abnormal Glucose and Lipid Metabolism. Frontiers in Endocrinology. 2019;10:703. doi: https://dx.doi.org/10.3389/fendo.2019.00703.

418. Shimba Y, Togawa H, Senoo N, Ikeda M, Miyoshi N, Morita A, et al. Skeletal muscle-specific PGC-1a overexpression prevents atherosclerosis in apolipoprotein e-knockout mice. Atherosclerosis Supplements. 2018;32:17-8.

419. Shimba Y, Togawa H, Senoo N, Ikeda M, Miyoshi N, Morita A, et al. Skeletal Muscle-specific PGC-1alpha Overexpression Suppresses Atherosclerosis in Apolipoprotein E-Knockout Mice. Scientific reports. 2019;9(1):4077. doi: https://dx.doi.org/10.1038/s41598-019-40643-1.

420. Shimba Y, Togawa H, Senoo N, Ikeda M, Miyoshi N, Morita A, et al. Skeletal Muscle-specific PGC-1α Overexpression Suppresses Atherosclerosis in Apolipoprotein E-Knockout Mice. Sci Rep. 2019;9(1):4077. Epub 2019/03/13. doi: 10.1038/s41598-019-40643-1. PubMed PMID: 30858489; PubMed Central PMCID: PMCPMC6411944.

421. Sillars A, Gao X, Gill JM, Freeman DJ. Adipocyte differentiation and inflammation relative to cardiovascular fitness. Obesity Facts. 2019;12(Supplement 1):89. doi: https://dx.doi.org/10.1159/000489691.

422. Silvester AJ, Aseer KR, Jang HJ, Ryu R, Kwon EY, Park JG, et al. Loss of DJ-1 promotes browning of white adipose tissue in diet-induced obese mice. Journal of Nutritional Biochemistry. 2018;61:56-67. doi: https://dx.doi.org/10.1016/j.jnutbio.2018.07.004.

423. Sliwicka E, Cison T, Pilaczynska-Szczesniak L, Ziemba A, Straburzynska-Lupa A. Effects of marathon race on selected myokines and sclerostin in middle-aged male amateur runners. Scientific reports. 2021;11(1):2813. doi: https://dx.doi.org/10.1038/s41598-021-82288-z.

424. Śliwicka E, Cisoń T, Pilaczyńska-Szcześniak Ł, Ziemba A, Straburzyńska-Lupa A. Effects of marathon race on selected myokines and sclerostin in middle-aged male amateur runners. Sci Rep. 2021;11(1):2813. Epub 2021/02/04. doi: 10.1038/s41598-021-82288-z. PubMed PMID: 33531538; PubMed Central PMCID: PMCPMC7854637.

425. Sneha D, Krishna KL, Mehdi S, Shreyas A, Swerna E. Skeletal muscle atrophy; a review on pathogenesis and biomarkers. International Journal of Pharmaceutical Research. 2021;13(2):4247-60. doi: https://dx.doi.org/10.31838/ijpr/2021.13.02.142.

426. Sobieszek G, Powrozek T, Mazurek M, Skwarek-Dziekanowska A, Malecka-Massalska T. Electrical and hormonal biomarkers in cachectic elderly women with chronic heart failure. Journal of Clinical Medicine. 2020;9(4):1021. doi: https://dx.doi.org/10.3390/jcm9041021.

427. Soleymanjahi S, Blanc V, Molitor EA, Riehl TE, Ciorba MA, Xie Y, et al. INTESTINE-SPECIFIC DELETION OF RNA-BINDING MOTIF PROTEIN 47 (RBM47) PROMOTES EPITHELIAL DEVELOPMENT AND ADAPTION TO ACUTE INJURY BY UP-REGULATING PROLIFERATIVE AND ANTI-OXIDATIVE RESPONSE PATHWAYS. Gastroenterology. 2020;158(6 Supplement 1):S-116. doi: https://dx.doi.org/10.1016/S0016-5085%2820%2930969-0.

428. Son H, Jang Y, Ahn J, Jung C, Ha T. P.1282, 6-Dimethoxy-1, 4-benzoquinone increases skeletal muscle mass through Akt/mTOR signaling pathway. Neuromuscular Disorders. 2019;29(Supplement 1):S85-S6. doi: https://dx.doi.org/10.1016/j.nmd.2019.06.184.

429. Son JS, Chae SA, Testroet ED, Du M, Jun HP. Exercise-induced myokines: a brief review of controversial issues of this decade. Expert Review of Endocrinology and Metabolism. 2018;13(1):51-8. doi: https://dx.doi.org/10.1080/17446651.2018.1416290.

430. Spiegelman BM. Banting lecture 2012: Regulation of adipogenesis: Toward new therapeutics for metabolic disease. Diabetes. 2013;62(6):1774-82. doi: https://dx.doi.org/10.2337/db12-1665.

431. Spiegelman BM. Banting Lecture 2012: Regulation of adipogenesis: toward new therapeutics for metabolic disease. Diabetes. 2013;62(6):1774-82. Epub 2013/05/25. doi: 10.2337/db12-1665. PubMed PMID: 23704518; PubMed Central PMCID: PMCPMC3661621.

432. Stenvinkel P, Carrero JJ, Von Walden F, Ikizler TA, Nader GA. Muscle wasting in end-stage renal disease promulgates premature death: Established, emerging and potential novel treatment strategies. Nephrology Dialysis Transplantation. 2016;31(7):1070-7. doi: https://dx.doi.org/10.1093/ndt/gfv122.

433. Sumsuzzman DM, Jin Y, Choi J, Yu JH, Lee TH, Hong Y. Pathophysiological role of endogenous irisin against tumorigenesis and metastasis: Is it a potential biomarker and therapeutic? Tumor Biology. 2019;41(12). doi: https://dx.doi.org/10.1177/1010428319892790.

434. Sumsuzzman DM, Jin Y, Choi J, Yu JH, Lee TH, Hong Y. Pathophysiological role of endogenous irisin against tumorigenesis and metastasis: Is it a potential biomarker and therapeutic? Tumour biology : the journal of the International Society for Oncodevelopmental Biology and Medicine. 2019;41(12):1010428319892790. Epub 2019/12/10. doi: 10.1177/1010428319892790. PubMed PMID: 31815594.

435. Sun X, Han F, Hou N. Irisin improves perivascular adipose tissue dysfunction via regulation of the heme oxygenase-1/adiponectin axis in diet-induced obese mice. Diabetologia. 2016;59(1 Supplement 1):S288. doi: https://dx.doi.org/10.1007/s00125-016-4046-9.

436. Sun X, Hou N, Han F. Irisin improves endothelial dysfunction by upregulating haeme oxygenase-1/adiponectin axis in perivascular adipose tissue in obese mice. Diabetologia. 2017;60(1 Supplement 1):S56. doi: https://dx.doi.org/10.1007/s00125-017-4350-z.

437. Suriano F, Van Hul M, Cani PD. Gut microbiota and regulation of myokine-adipokine function. Current Opinion in Pharmacology. 2020;52:9-17. doi: https://dx.doi.org/10.1016/j.coph.2020.03.006.

438. Takasawa S, Shobatake R, Itaya-Hironaka A, Makino M, Sakuramoto-Tsuchida S, Uchiyama T, et al. Up-regulation of IL-8, osteonectin and myonectin mRNAs by intermittent hypoxia via OCT1- and NRF2-mediatedmechanisms in skeletal muscle cells. Diabetologia. 2020;63(SUPPL 1):S221-S2. doi: https://dx.doi.org/10.1007/s00125-020-05221-5.

439. Taken K, Aslan R, Eryilmaz R, Alp HH, Huyut Z, Dönmez M. Serum irisin is a novel biomarker for bladder cancer detection. Int Urol Nephrol. 2022;54(1):55-61. Epub 2021/11/23. doi: 10.1007/s11255-021-03074-4. PubMed PMID: 34807348.

440. Taken K, Aslan R, Eryilmaz R, Alp HH, Huyut Z, Dönmez M. Response to Letter to the Editor 'Serum irisin concentration in patients with bladder cancer'. Int Urol Nephrol. 2022;54(6):1247-8. Epub 2022/04/21. doi: 10.1007/s11255-022-03215-3. PubMed PMID: 35441909.

441. Taken K, Aslan R, Eryilmaz R, Alp HH, Huyut Z, Donmez MI. Response to Letter to the Editor 'Serum irisin concentration in patients with bladder cancer'. International Urology and Nephrology. 2022;54(6):1247-8. doi: https://dx.doi.org/10.1007/s11255-022-03215-3.

442. Taken K, Aslan R, Eryilmaz R, Alp HH, Huyut Z, Donmez MI. Serum irisin is a novel biomarker for bladder cancer detection. International Urology and Nephrology. 2022;54(1):55-61. doi: https://dx.doi.org/10.1007/s11255-021-03074-4.

443. Tan X, van Egmond LT, Cedernaes J, Benedict C. The role of exercise-induced peripheral factors in sleep regulation. Molecular Metabolism. 2020;42:101096. doi: https://dx.doi.org/10.1016/j.molmet.2020.101096.

444. Tan Y, Ouyang H, Xiao X, Zhong J, Dong M. Irisin ameliorates septic cardiomyopathy via inhibiting DRP1-related mitochondrial fission and normalizing the JNK-LATS2 signaling

pathway. Cell Stress and Chaperones. 2019;24(3):595-608. doi: https://dx.doi.org/10.1007/s12192-019-00992-2.

445. Tappia PS, Blewett H. Nutrition and cardiovascular health. International Journal of Molecular Sciences. 2020;21(7):2284. doi: https://dx.doi.org/10.3390/ijms21072284.

446. Tejeda ME, Canto P, Tenorio-Torres A, Orozco-Arguelles L, Coral-Vazquez RM, Zentella-Dehesa A, et al. Increased FNDC5/IRISIN protein expression in breast cancer tissue is associated with obesity in postmenopausal women. Journal of Clinical Pathology. 2021. doi: https://dx.doi.org/10.1136/jclinpath-2020-207249.

447. Tejeda ME, Canto P, Tenorio-Torres A, Orozco-Arguelles L, Coral-Vázquez RM, Zentella-Dehesa A, et al. Increased FNDC5/IRISIN protein expression in breast cancer tissue is associated with obesity in postmenopausal women. J Clin Pathol. 2021. Epub 2021/06/05. doi: 10.1136/jclinpath-2020-207249. PubMed PMID: 34083413.

448. Tekin S, Erden Y, Etem E, Tektemur A, Kirbag S, Sandal S. Effect of intracerebroventricular irisin injection on the uncoupling protein expression in the rat brain. Acta Physiologica. 2014;211(SUPPL. 697):157. doi: https://dx.doi.org/10.1111/apha.12362.

449. Testai L, De Leo M, Flori L, Polini B, Braca A, Nieri P, et al. Contribution of irisin pathway in protective effects of mandarin juice (Citrus reticulata Blanco) on metabolic syndrome in rats fed with high fat diet. Phytotherapy Research. 2021;35(8):4324-33. doi: https://dx.doi.org/10.1002/ptr.7128.

450. Thomas RJ, Kenfield SA, Jimenez A. Exercise-induced biochemical changes and their potential influence on cancer: a scientific review. British journal of sports medicine. 2017;51(8):640-4. doi: https://dx.doi.org/10.1136/bjsports-2016-096343.

451. Thomas RJ, Kenfield SA, Jimenez A. Exercise-induced biochemical changes and their potential influence on cancer: a scientific review. Br J Sports Med. 2017;51(8):640-4. Epub 2016/12/21. doi: 10.1136/bjsports-2016-096343. PubMed PMID: 27993842; PubMed Central PMCID: PMCPMC5466928.

452. Tokunbo O, Abayomi T, Adekomi D, Oyeyipo I. Covid-19: Sitting is the new smoking; the role of exercise in augmenting the immune system among the elderly. African Health Sciences. 2021;21(1):189-93. doi: https://dx.doi.org/10.4314/ahs.v21i1.25.

453. Trovato E, Di Felice V, Barone R. Extracellular vesicles: Delivery vehicles of myokines. Frontiers in Physiology. 2019;10(MAY):522. doi: https://dx.doi.org/10.3389/fphys.2019.00522.

454. Tsai JS, Wang SY, Chang CH, Chen CY, Wen CJ, Chen GY, et al. Identification of traumatic acid as a potential plasma biomarker for sarcopenia using a metabolomics-based approach. J Cachexia Sarcopenia Muscle. 2022;13(1):276-86. Epub 2021/12/24. doi: 10.1002/jcsm.12895. PubMed PMID: 34939349; PubMed Central PMCID: PMCPMC8818620.

455. Tsai JS, Wang SY, Chang CH, Wen CJ, Chen GY, Kuo CH, et al. Identification of traumatic acid as a potential plasma biomarker for sarcopenia using a metabolomics-based approach. Journal of Cachexia, Sarcopenia and Muscle. 2022;13(1):276-86. doi: https://dx.doi.org/10.1002/jcsm.12895.

456. Tsai YC, Chen JH, Lee YM, Yen MH, Cheng PY. Raspberry ketone promotes FNDC5 protein expression via HO-1 upregulation in 3T3-L1 adipocytes. Chinese Journal of Physiology. 2022;65(2):80-6. doi: https://dx.doi.org/10.4103/cjp.cjp_95_21.

457. Tseng YH, Yeh YH, Chen WJ, Lin KH. Emerging regulation and function of betatrophin. International Journal of Molecular Sciences. 2014;15(12):23640-57. doi: https://dx.doi.org/10.3390/ijms151223640.

458. Tsiani E, Tsakiridis N, Kouvelioti R, Jaglanian A, Klentrou P. Current evidence of the role of the myokine irisin in cancer. Cancers. 2021;13(11):2628. doi: https://dx.doi.org/10.3390/cancers13112628.

459. Tsiani E, Tsakiridis N, Kouvelioti R, Jaglanian A, Klentrou P. Current Evidence of the Role of the Myokine Irisin in Cancer. Cancers (Basel). 2021;13(11). Epub 2021/06/03. doi: 10.3390/cancers13112628. PubMed PMID: 34071869; PubMed Central PMCID: PMCPMC8199282.

460. Ugur K, Aydin S, Kuloglu T, Artas G, Kocdor MA, Sahin İ, et al. Comparison of irisin hormone expression between thyroid cancer tissues and oncocytic variant cells. Cancer Manag Res. 2019;11:2595-603. Epub 2019/05/23. doi: 10.2147/cmar.s201979. PubMed PMID: 31114326; PubMed Central PMCID: PMCPMC6497896.

461. Ugur K, Aydin S, Kuloglu T, Artas G, Kocdor MA, Sahin I, et al. Comparison of irisin hormone expression between thyroid cancer tissues and oncocytic variant cells. Cancer Management and Research. 2019;11:2595-603. doi: https://dx.doi.org/10.2147/CMAR.S201979.

462. Us Altay D, Keha E, Alver A, Karaguzel E, Mentese A, Fidan E. The diagnostic value of FNDC5/irisin in renal and gastric cancer. Clinical Chemistry and Laboratory Medicine. 2014;52(SUPPL. 1):S477. doi: https://dx.doi.org/10.1515/cclm-2014-4013.

463. Us Altay D, Keha EE, Ozer Yaman S, Ince I, Alver A, Erdogan B, et al. Investigation of the expression of irisin and some cachectic factors in mice with experimentally induced gastric cancer. Qjm. 2016;109(12):785-90. Epub 2016/06/04. doi: 10.1093/qjmed/hcw074. PubMed PMID: 27256459.

464. Us Altay D, Onder S, Etgu F, Uner A, Noyan T. A newly identified myokine: irisin, and its relationship with chronic spontaneous urticaria and inflammation. Archives of dermatological research. 2022. doi: https://dx.doi.org/10.1007/s00403-022-02378-4.

465. Us Altay D, Onder S, Etgu F, Uner A, Noyan T. A newly identified myokine: irisin, and its relationship with chronic spontaneous urticaria and inflammation. Arch Dermatol Res. 2022:1-6. Epub 2022/08/11. doi: 10.1007/s00403-022-02378-4. PubMed PMID: 35948647; PubMed Central PMCID: PMCPMC9365215.

466. Uslu H, Uslu GA. Evaluating the effects of Prunus laurocerasus seed, fruit and leaf extracts on hyperglycaemia, insulin sensitivity and anti-oxidative activities in experimental diabetes in rat. Thai Journal of Veterinary Medicine. 2021;51(4):667-73. doi: https://dx.doi.org/10.14456/tjvm.2021.80.

467. Vaughan RA, Gannon NP, Mermier CM, Conn CA. Irisin, a unique non-inflammatory myokine in stimulating skeletal muscle metabolism. Journal of Physiology and Biochemistry. 2015;71(4):679-89. doi: https://dx.doi.org/10.1007/s13105-015-0433-9.

468. Vaughan RA, Gannon NP, Mermier CM, Conn CA. Irisin, a unique non-inflammatory myokine in stimulating skeletal muscle metabolism. J Physiol Biochem. 2015;71(4):679-89. Epub 2015/09/25. doi: 10.1007/s13105-015-0433-9. PubMed PMID: 26399516.

469. Vesel CB, Lubbers ER, List EO, Kopchick JJ, Berryman DE. Quantification of brown adipocyte markers in white adipose tissues of mice with altered Growth Hormone action. Growth Hormone and IGF Research. 2012;22(SUPPL. 1):S63-S4.

470. Vicenti G, Bortone I, Bizzoca D, Sardone R, Belluati A, Solarino G, et al. Bridging the gap between serum biomarkers and biomechanical tests in musculoskeletal ageing. Journal of Biological Regulators and Homeostatic Agents. 2020;34(4 Supplement 3):263-74.

471. Viitasalo A, Atalay M, Pihlajamaki J, Jaaskelainen J, Korkmaz A, Kaminska D, et al. The 148 M allele of the PNPLA3 is associated with plasma irisin levels in a population sample of Caucasian children: The PANIC Study. Metabolism: Clinical and Experimental. 2015;64(7):793-6. doi: https://dx.doi.org/10.1016/j.metabol.2015.01.010.

472. Viitasalo A, Atalay M, Pihlajamäki J, Jääskeläinen J, Korkmaz A, Kaminska D, et al. The 148 M allele of the PNPLA3 is associated with plasma irisin levels in a population sample of Caucasian children: The PANIC Study. Metabolism. 2015;64(7):793-6. Epub 2015/02/14. doi: 10.1016/j.metabol.2015.01.010. PubMed PMID: 25676803.

473. Wagner IV, Yango P, Tran ND, Svechnikov K, Soder O. Adipocytokines prevent pubertal maturation of human sertoli cells. Journal fur Reproduktionsmedizin und Endokrinologie. 2015;12(5):473-4.

474. Wang B, Zheng B, Lu Y, Huang D, Liu J, Song J, et al. FNDC4 acts as an extracellular factor to promote the invasiveness of hepatocellular carcinoma partly via the PI3K/Akt signalling pathway. Cancer Medicine. 2021;10(20):7242-52. doi: https://dx.doi.org/10.1002/cam4.4225.

475. Wang B, Zheng B, Lu Y, Huang D, Liu J, Song J, et al. FNDC4 acts as an extracellular factor to promote the invasiveness of hepatocellular carcinoma partly via the PI3K/Akt signalling pathway. Cancer Med. 2021;10(20):7242-52. Epub 2021/08/22. doi: 10.1002/cam4.4225. PubMed PMID: 34418326; PubMed Central PMCID: PMCPMC8525097.

476. Wang J, Xu F, Zheng Y, Cheng X, Zhang P, Zhao H. Irisin Ameliorates Hypoxia/Reoxygenation-Induced Inflammation and Apoptosis in PC12 Cells by Inhibiting TLR4/MYD88 Signaling Pathway. Current Topics in Nutraceutical Research. 2019;17(3):329-36. doi: https://dx.doi.org/10.37290/ctnr2641-452x.17:329-336.

477. Wang T. Searching for the link between inflammaging and sarcopenia. Ageing Research Reviews. 2022;77:101611. doi: https://dx.doi.org/10.1016/j.arr.2022.101611.

478. Wang T, Maltez MT, Lee HW, Ahmad M, Wang HW, Leenen FHH. Effect of exercise training on the FNDC5/BDNF pathway in spontaneously hypertensive rats. Physiological Reports. 2019;7(24):e14323. doi: https://dx.doi.org/10.14814/phy2.14323.

479. Wang X, Li M, Zheng R, Cui T, Qin J, Su Z, et al. High irisin and low bdnf levels in aqueous humor of high myopia. Advances in Clinical and Experimental Medicine. 2020;30(9). doi: https://dx.doi.org/10.17219/ACEM/125428.

480. Wang X, Li M, Zheng R, Cui T, Qin J, Su Z, et al. High irisin and low BDNF levels in aqueous humor of high myopia. Advances in clinical and experimental medicine : official organ Wroclaw Medical University. 2021;30(9):893-904. Epub 2021/06/24. doi: 10.17219/acem/125428. PubMed PMID: 34160912.

481. Wang X, Mao L, Li C, Hui Y, Yu Z, Sun M, et al. The potential role of FNDC5/irisin in various liver diseases: awakening the sleeping beauties. Expert Reviews in Molecular Medicine. 2022. doi: https://dx.doi.org/10.1017/erm.2022.19.

482. Wang X, Mao L, Li C, Hui Y, Yu Z, Sun M, et al. The potential role of FNDC5/irisin in various liver diseases: awakening the sleeping beauties. Expert Rev Mol Med. 2022;24:e23. Epub 2022/06/14. doi: 10.1017/erm.2022.19. PubMed PMID: 35695040.

483. Wang Y, Liu H, Sun N, Li J, Peng X, Jia Y, et al. Irisin: A Promising Target for Ischemia-Reperfusion Injury Therapy. Oxidative Medicine and Cellular Longevity. 2021;2021:5391706. doi: https://dx.doi.org/10.1155/2021/5391706.

484. Wang Y, Tian M, Tan J, Pei X, Lu C, Xin Y, et al. Irisin ameliorates neuroinflammation and neuronal apoptosis through integrin alphaVbeta5/AMPK signaling pathway after intracerebral hemorrhage in mice. Journal of Neuroinflammation. 2022;19(1):82. doi: https://dx.doi.org/10.1186/s12974-022-02438-6.

485. Wang YF, Chen X, Sha L, Kendrick KM, Lee LTO, Cheng CHK. Editorial: Neuroendocrine Research in Health and Disease. Frontiers in Neuroscience. 2020;14:176. doi: https://dx.doi.org/10.3389/fnins.2020.00176.

486. Waseem R, Anwar S, Khan S, Shamsi A, Hassan MI, Anjum F, et al. Map/microtubule affinity regulating kinase 4 inhibitory potential of irisin: A new therapeutic strategy to combat cancer and alzheimer's disease. International Journal of Molecular Sciences. 2021;22(20):10986. doi: https://dx.doi.org/10.3390/ijms222010986.

487. Waseem R, Anwar S, Khan S, Shamsi A, Hassan MI, Anjum F, et al. MAP/Microtubule Affinity Regulating Kinase 4 Inhibitory Potential of Irisin: A New Therapeutic Strategy to Combat Cancer and Alzheimer's Disease. Int J Mol Sci. 2021;22(20). Epub 2021/10/24. doi: 10.3390/ijms222010986. PubMed PMID: 34681645; PubMed Central PMCID: PMCPMC8537121.

488. Waseem R, Shamsi A, Mohammad T, Hassan MI, Kazim SN, Chaudhary AA, et al. FNDC5/Irisin: Physiology and Pathophysiology. Molecules (Basel, Switzerland). 2022;27(3). doi: https://dx.doi.org/10.3390/molecules27031118.

489. Waseem R, Shamsi A, Mohammad T, Hassan MI, Kazim SN, Chaudhary AA, et al. FNDC5/Irisin: Physiology and Pathophysiology. Molecules. 2022;27(3). Epub 2022/02/16. doi: 10.3390/molecules27031118. PubMed PMID: 35164383; PubMed Central PMCID: PMCPMC8838669.

490. Whitham M, Febbraio MA. The ever-expanding myokinome: Discovery challenges and therapeutic implications. Nature Reviews Drug Discovery. 2016;15(10):719-29. doi: https://dx.doi.org/10.1038/nrd.2016.153.

491. Witek K, Zurek P, Zmijewski P, Jaworska J, Lipinska P, Dzedzej-Gmiat A, et al. Myokines in response to a tournament season among young tennis players. BioMed Research International. 2016;2016:1460892. doi: https://dx.doi.org/10.1155/2016/1460892.

492. Wojcik D, Bilski J, Mazur-Bialy A, Surmiak M, Hubalewska-Mazgaj M, Magierowski M, et al. NOVEL INSIGHT INTO MECHANISM OF PROTECTIVE ACTION OF INTESTINAL ALKALINE PHOSPHATASE AGAINST EXPERIMENTAL COLITIS IN OBESE MICE WITH MODERATE PHYSICAL ACTIVITY. INVOLVEMENT OF MICROBIOTA, MYOKINES RELEASED FROM SKELETAL MUSCLE AND PROINFLAMMATORY FACTORS. Gastroenterology. 2019;156(6 Supplement 1):S-621. doi: https://dx.doi.org/10.1016/S0016-5085%2819%2938448-3.

493. Wojcik D, Danielak A, Mazur-Bialy A, Surmiak M, Magierowski M, Sliwowski Z, et al. INTESTINAL PROTECTION INDUCED BY TREATMENT WITH ALKALINE PHOSPHATASE ON THE SEVERITY OF EXPERIMENTAL COLITIS IN OBESE MICE SUBJECTED TO FORCED TREADMILL RUNNING. ROLE OF PROINFLAMMATORY BIOMARKERS, INTESTINAL BARRIER PROTEINS AND OXIDATIVE STRESS. Gastroenterology. 2020;158(6 Supplement 1):S-763. doi: https://dx.doi.org/10.1016/S0016-5085%2820%2932582-8.

494. Wojcik D, Mazur-Bialy A, Bilski J, Surmiak M, Hubalewska-Mazgaj M, Magierowski M, et al. Role of intestinal alkaline phosp hatase , myokines and microbiome in amelioration of experimental colitis in voluntary running wheel activity obese mice fed fat diet. United European Gastroenterology Journal. 2019;7(8 Supplement):37-8. doi: https://dx.doi.org/10.1177/205064061985467.

495. Wojcik D, Mazur-Bialy A, Surmiak M, Danielak A, Kwiecien S, Magierowski M, et al. Novel insight into mechanism of healing of experimental colitis by intestinal alkaline phosphatase in obese mice subjected to forced treadmill exercise. involvement of oxidative stress, proinflammatory biomarkers and intestinal barrier proteins. United European Gastroenterology Journal. 2020;8(8 SUPPL):320. doi: https://dx.doi.org/10.1177/2050640620927345.

496. Wozniak S, Nowinska K, Chabowski M, Dziegiel P. Significance of Irisin (FNDC5) Expression in Colorectal Cancer. In Vivo. 2022;36(1):180-8. doi: https://dx.doi.org/10.21873/invivo.12689.

497. Wu SE, Hsu JC, Chang YL, Chuang HC, Chiu YL, Chen WL. Benzo[a]pyrene exposure in muscle triggers sarcopenia through aryl hydrocarbon receptor-mediated reactive oxygen species production. Ecotoxicology and Environmental Safety. 2022;239:113599. doi: https://dx.doi.org/10.1016/j.ecoenv.2022.113599.

498. Wuensch T, Wizenty J, Quint J, Spitz W, Bosma M, Becker O, et al. Expression analysis of fibronectin type III domain-containing (FNDC) genes in inflammatory bowel disease and colorectal cancer. Gastroenterology Research and Practice. 2019;2019:3784172. doi: https://dx.doi.org/10.1155/2019/3784172.

499. Xia T, Zhang X, Wang Y, Deng D. Effect of maternal hypothyroidism during pregnancy on insulin resistance, lipid accumulation, and mitochondrial dysfunction in skeletal muscle of fetal rats. Bioscience Reports. 2018;38(4):BSR20171731. doi: https://dx.doi.org/10.1042/BSR20171731.

500. Xie T, Leung PS. Roles of FGF21 and irisin in obesity-related diabetes and pancreatic diseases. Journal of Pancreatology. 2020;3(1):29-34. doi: https://dx.doi.org/10.1097/JP9.0000000000000039.

501. Xu L, Shen L, Yu X, Li P, Wang Q, Li C. Effects of irisin on osteoblast apoptosis and osteoporosis in postmenopausal osteoporosis rats through upregulating Nrf2 and inhibiting

NLRP3 inflammasome. Experimental and Therapeutic Medicine. 2020;19(2):1084-90. doi: https://dx.doi.org/10.3892/etm.2019.8313.

502. Xu L, Shen L, Yu X, Li P, Wang Q, Li C. Effects of irisin on osteoblast apoptosis and osteoporosis in postmenopausal osteoporosis rats through upregulating Nrf2 and inhibiting NLRP3 inflammasome. Exp Ther Med. 2020;19(2):1084-90. Epub 2020/02/06. doi: 10.3892/etm.2019.8313. PubMed PMID: 32010273; PubMed Central PMCID: PMCPMC6966163.

503. Yaffe MB, Gough NR. 2012: Signaling breakthroughs of the year. Science Signaling. 2013;6(256):eg1. doi: https://dx.doi.org/10.1126/scisignal.2003881.

504. Yalcin AD, Bulut T, Celik B, Genc GE, Gumuslu S. Anti-IgE mab treatment decreased IL-1B and Irisin but increased chemerin without any impact on NK cells and APC cells in cases of severe persistent asthma. Respirology. 2016;21(Supplement 3):46. doi: https://dx.doi.org/10.1111/resp.12939-14.

505. Yamakage H, Tanaka M, Inoue T, Odori S, Kusakabe T, Satoh-Asahara N. Effects of dapagliflozin on the serum levels of fibroblast growth factor 21 and myokines and muscle mass in Japanese patients with type 2 diabetes: A randomized, controlled trial. Journal of Diabetes Investigation. 2020;11(3):653-61. doi: https://dx.doi.org/10.1111/jdi.13179.

506. Yang BC, Leung PS. Irisin Is a Positive Regulator for Ferroptosis in Pancreatic Cancer. Molecular Therapy - Oncolytics. 2020;18:457-66. doi: https://dx.doi.org/10.1016/j.omto.2020.08.002.

507. Yang BC, Leung PS. Irisin Is a Positive Regulator for Ferroptosis in Pancreatic Cancer. Molecular therapy oncolytics. 2020;18:457-66. Epub 2020/09/22. doi: 10.1016/j.omto.2020.08.002. PubMed PMID: 32953980; PubMed Central PMCID: PMCPMC7475648.

508. Yang Z, Zhang W, Ren X, Tu C, Li Z. Exosomes: A Friend or Foe for Osteoporotic Fracture? Frontiers in Endocrinology. 2021;12:679914. doi: https://dx.doi.org/10.3389/fendo.2021.679914.

509. Yardim M, Kuloglu T, Ozercan IH, Kenanoglu O, Aydin S. Irisin immunoreactivity in hematological malignancies. FEBS Journal. 2016;283(Supplement 1):212. doi: https://dx.doi.org/10.1111/febs.13808.

510. Yasukawa K, Liew LC, Hagiwara K, Hironaka-Mitsuhashi A, Qin XY, Furutani Y, et al. MicroRNA-493-5p-mediated repression of the MYCN oncogene inhibits hepatic cancer cell growth and invasion. Cancer Science. 2020;111(3):869-80. doi: https://dx.doi.org/10.1111/cas.14292.

511. Ye W, Wang J, Lin D, Ding Z. The immunomodulatory role of irisin on osteogenesis via AMPK-mediated macrophage polarization. International Journal of Biological Macromolecules. 2020;146:25-35. doi: https://dx.doi.org/10.1016/j.ijbiomac.2019.12.028.

512. Yin C, Hu W, Wang M, Lv W, Jia T, Xiao Y. Irisin as a mediator between obesity and vascular inflammation in Chinese children and adolescents. Nutrition, Metabolism and Cardiovascular Diseases. 2020;30(2):320-9. doi: https://dx.doi.org/10.1016/j.numecd.2019.09.025.

513. Yu K, Liu D, Yang J, Tan L, Zhang D. Irisin enhances osteogenic differentiation of mouse MC3T3-E1 cells via upregulating osteogenic genes. Experimental and Therapeutic Medicine. 2021;21(6):580. doi: https://dx.doi.org/10.3892/etm.2021.10012.

514. Yu M, Tsai SF, Kuo YM. The therapeutic potential of anti-inflammatory exerkines in the treatment of atherosclerosis. International Journal of Molecular Sciences. 2017;18(6):1260. doi: https://dx.doi.org/10.3390/ijms18061260.

515. Yu Y, Tian T, Tan S, Wu P, Guo Y, Li M, et al. MicroRNA-665-3p exacerbates nonalcoholic fatty liver disease in mice. Bioengineered. 2022;13(2):2927-42. doi: https://dx.doi.org/10.1080/21655979.2021.2017698.

516. Zebrowska A, Sikora M, Konarska A, Zwierzchowska A, Kaminski T, Robins A, et al. Moderate intensity exercise in hypoxia increases IGF-1 bioavailability and serum irisin in individuals with type 1 diabetes. Therapeutic Advances in Endocrinology and Metabolism. 2020;11. doi: https://dx.doi.org/10.1177/2042018820925326.

517. Zhang D, Tan X, Shi G. Research progress in the role of irisin in tumors. Tumor. 2017;37(5):524-9. doi: https://dx.doi.org/10.3781/j.issn.1000-7431.2017.55.099.

518. Zhang D, Tan X, Tang N, Huang F, Chen Z, Shi G. Review of research on the role of irisin in tumors. OncoTargets and Therapy. 2020;13:4423-30. doi: https://dx.doi.org/10.2147/OTT.S245178.

519. Zhang D, Tan X, Tang N, Huang F, Chen Z, Shi G. Review of Research on the Role of Irisin in Tumors. Onco Targets Ther. 2020;13:4423-30. Epub 2020/06/18. doi: 10.2147/ott.s245178. PubMed PMID: 32547073; PubMed Central PMCID: PMCPMC7245464.

520. Zhang D, Zhang P, Li L, Tang N, Huang F, Kong X, et al. Irisin functions to inhibit malignant growth of human pancreatic cancer cells via downregulation of the PI3K/AKT signaling pathway. OncoTargets and Therapy. 2019;12:7243-9. doi: https://dx.doi.org/10.2147/OTT.S214260.

521. Zhang D, Zhang P, Li L, Tang N, Huang F, Kong X, et al. Irisin functions to inhibit malignant growth of human pancreatic cancer cells via downregulation of the PI3K/AKT signaling pathway. Onco Targets Ther. 2019;12:7243-9. Epub 2019/10/01. doi: 10.2147/ott.s214260. PubMed PMID: 31564907; PubMed Central PMCID: PMCPMC6732507.

522. Zhang H, Wu X, Liang J, Kirberger M, Chen N. Irisin, an exercise-induced bioactive peptide beneficial for health promotion during aging process. Ageing Research Reviews. 2022;80:101680. doi: https://dx.doi.org/10.1016/j.arr.2022.101680.

523. Zhang H, Wu X, Liang J, Kirberger M, Chen N. Irisin, an exercise-induced bioactive peptide beneficial for health promotion during aging process. Ageing Res Rev. 2022;80:101680. Epub 2022/07/07. doi: 10.1016/j.arr.2022.101680. PubMed PMID: 35793739.

524. Zhang J, Ke M, Ren Y, Bi J, Du Z, Zhang M, et al. Serum Irisin Predicts Posthepatectomy Complications in Patients with Hepatocellular Carcinoma. Disease Markers. 2019;2019:9850191. doi: https://dx.doi.org/10.1155/2019/9850191.

525. Zhang J, Ke M, Ren Y, Bi J, Du Z, Zhang M, et al. Serum Irisin Predicts Posthepatectomy Complications in Patients with Hepatocellular Carcinoma. Dis Markers.

2019;2019:9850191. Epub 2020/01/25. doi: 10.1155/2019/9850191. PubMed PMID: 31976024; PubMed Central PMCID: PMCPMC6955133.

526. Zhang L, Sun Y. Muscle-Bone Crosstalk in Chronic Obstructive Pulmonary Disease. Frontiers in Endocrinology. 2021;12:724911. doi: https://dx.doi.org/10.3389/fendo.2021.724911.

527. Zhang R, Ji J, Zhou X, Li R. Irisin Pretreatment Protects Kidneys against Acute Kidney Injury Induced by Ischemia/Reperfusion via Upregulating the Expression of Uncoupling Protein 2. BioMed Research International. 2020;2020:6537371. doi: https://dx.doi.org/10.1155/2020/6537371.

528. Zhang X, Tong Y, Zhu F, Liang F, Lu A. Fndc5 reprograms m1/m2 macrophage polarization and attenuates atherosclerosis-associated rheumatoid arthritis via modulation of ampk/nfat5 signalling. Latin American Journal of Pharmacy. 2020;39(11):2272-8.

529. Zhang Y, Kim JS, Wang TZ, Newton RU, Galvao DA, Gardiner RA, et al. Potential Role of Exercise Induced Extracellular Vesicles in Prostate Cancer Suppression. Frontiers in Oncology. 2021;11:746040. doi: https://dx.doi.org/10.3389/fonc.2021.746040.

530. Zhang Z, Zhang C, Zhang S. Irisin Activates M1 Macrophage and Suppresses Th2-Type Immune Response in Rats with Pelvic Inflammatory Disease. Evidence-based Complementary and Alternative Medicine. 2022;2022:5215915. doi: https://dx.doi.org/10.1155/2022/5215915.

531. Zhang ZP, Zhang XF, Li H, Liu TJ, Zhao QP, Huang LH, et al. Serum irisin associates with breast cancer to spinal metastasis. Medicine (United States). 2018;97(17):e0524. doi: https://dx.doi.org/10.1097/MD.0000000000010524.

532. Zhang ZP, Zhang XF, Li H, Liu TJ, Zhao QP, Huang LH, et al. Serum irisin associates with breast cancer to spinal metastasis. Medicine. 2018;97(17):e0524. Epub 2018/04/29. doi: 10.1097/md.0000000000010524. PubMed PMID: 29703023; PubMed Central PMCID: PMCPMC5944558 declare that this manuscript has not been submitted or is not simultaneously being submitted elsewhere, and that no portion of the data has been or will be published in proceedings or transactions of meetings or symposium volumes. The authors report no conflicts of interest.

533. Zhu H, Liu M, Zhang N, Pan H, Lin G, Li N, et al. Serum and adipose tissue mRNA levels of ATF3 and FNDC5/irisin in colorectal cancer patients with or without obesity. Frontiers in Physiology. 2018;9(SEP):1125. doi: https://dx.doi.org/10.3389/fphys.2018.01125.

534. Zhu H, Liu M, Zhang N, Pan H, Lin G, Li N, et al. Serum and Adipose Tissue mRNA Levels of ATF3 and FNDC5/Irisin in Colorectal Cancer Patients With or Without Obesity. Front Physiol. 2018;9:1125. Epub 2018/09/25. doi: 10.3389/fphys.2018.01125. PubMed PMID: 30246803; PubMed Central PMCID: PMCPMC6140752.

535. Zhu T, Zhang W, Zhang Y, Lu E, Liu H, Liu X, et al. Irisin/FNDC5 inhibits the epithelial-mesenchymal transition of epithelial ovarian cancer cells via the PI3K/Akt pathway. Archives of Gynecology and Obstetrics. 2022. doi: https://dx.doi.org/10.1007/s00404-022-06427-1.

536. Zhu T, Zhang W, Zhang Y, Lu E, Liu H, Liu X, et al. Irisin/FNDC5 inhibits the epithelial-mesenchymal transition of epithelial ovarian cancer cells via the PI3K/Akt pathway. Arch

Gynecol Obstet. 2022. Epub 2022/02/15. doi: 10.1007/s00404-022-06427-1. PubMed PMID: 35156135.

537. Zsuga J, Tajti G, Papp C, Juhasz B, Gesztelyi R. FNDC5/irisin, a molecular target for boosting reward-related learning and motivation. Medical Hypotheses. 2016;90:23-8. doi: https://dx.doi.org/10.1016/j.mehy.2016.02.020.

538. Zsuga J, Tajti G, Papp C, Juhasz B, Gesztelyi R. FNDC5/irisin, a molecular target for boosting reward-related learning and motivation. Med Hypotheses. 2016;90:23-8. Epub 2016/04/12. doi: 10.1016/j.mehy.2016.02.020. PubMed PMID: 27063080.

539. Zunner BEM, Wachsmuth NB, Eckstein ML, Scherl L, Schierbauer JR, Haupt S, et al. Myokines and Resistance Training: A Narrative Review. International Journal of Molecular Sciences. 2022;23(7):3501. doi: https://dx.doi.org/10.3390/ijms23073501.

540. Zunner BEM, Wachsmuth NB, Eckstein ML, Scherl L, Schierbauer JR, Haupt S, et al. Myokines and Resistance Training: A Narrative Review. Int J Mol Sci. 2022;23(7). Epub 2022/04/13. doi: 10.3390/ijms23073501. PubMed PMID: 35408868; PubMed Central PMCID: PMCPMC8998961.

541. Zybek-Kocik A, Sawicka-Gutaj N, Szczepanek-Parulska E, Andrusiewicz M, Waligorska-Stachura J, Bialas P, et al. The association between irisin and muscle metabolism in different thyroid disorders. Clinical Endocrinology. 2018;88(3):460-7. doi: https://dx.doi.org/10.1111/cen.13527.

542. Zybek-Kocik A, Sawicka-Gutaj N, Szczepanek-Parulska E, Andrusiewicz M, Waligórska-Stachura J, Białas P, et al. The association between irisin and muscle metabolism in different thyroid disorders. Clin Endocrinol (Oxf). 2018;88(3):460-7. Epub 2017/12/03. doi: 10.1111/cen.13527. PubMed PMID: 29197093.

543. Zsuga J, Tajti G, Papp C, Juhasz B, Gesztelyi R. FNDC5/irisin, a molecular target for boosting reward-related learning and motivation. Medical hypotheses. 2016;90:23-8. Epub 2016/04/12. doi: 10.1016/j.mehy.2016.02.020. PubMed PMID: 27063080.

544. Pazgan-Simon M. Serum irisin level a new possible marker of HCC progression. Conference abstract.

545. Kurrat A, Blei, T, Kulling, SE, Müller, D, Piechotta, M, Soukup, ST, Weigt, C & Diel, PR. Effects of lifelong dietary phytoestrogen exposure on the susceptibility to develop obesity – A study in ovariectomized and intact female Wistar rats. ENDOCRINE REVIEWS,. 2015;30.

546. Irisin immunoreactivity in hematological malignancies. Registered trial

547. Irisin a candidate biomarker for distinguishing thyroid oncocytic variant carcinomas, DE GRUYTER- II. Türkiye in vitro Diyagnostik Sempozyumu Turk J Biochem 2017 | Volume 42 I Supplement Issue 2 “BİYOBELİRTEÇLER”

548. Irisin immunoreactivity in hematological malignancies., FEBS Journal. Conference: 41st FEBS Congress: Molecular and Systems Biology for a Better Life. Turkey. 283 (Supplement 1) (pp 212), 2016. Date of Publication: September 2016., Yardim M., Kuloglu T., Ozercan I.H., Kenanoglu O., Aydin S.

549. New method of Irisin levels identification in gastric cancer patients with cachexia., FEBS Journal. Conference: 42nd Congress of the Federation of European Biochemical Societies,

FEBS 2017. Israel. 284 (Supplement 1) (pp 284), 2017. Date of Publication: September 2017., Drabik A., Mudlaff K., Silberring J., Kulig J., Sierzega M.

550. Is the association between physical activity and colorectal adenoma mediated by muscle-derived myokines?., United European Gastroenterology Journal. Conference: 24th United European Gastroenterology Week, UEG 2016. Austria. 4 (5 Supplement 1) (pp A658-A659), 2016. Date of Publication: October 2016., Namasivayam V. Pryseley A.N., Wong W.Y., Wan W.K., Lim K.H., Azhar R., Connolly J.

551. Irisin, an exercise-induced hormone, targets glioblastoma tumor in vivo PET/CT imaging may serve as a novel theranostic agent., European Journal of Nuclear Medicine and Molecular Imaging. Conference: 30th Annual Congress of the European Association of Nuclear Medicine, EANM 2017. Austria. 44 (2 Supplement 1) (pp S468), 2017. Date of Publication: 2017. Chiu H., Chang W., Lin Y., Chung Y. ,Yen T., Huang F., Huang C.

552. Fibronectin type III domain containing 5(FNDC5) gene expression level in colorectal cancer cell lines., European Journal of Human Genetics. Conference: 51st European Society of Human Genetics Conference. Italy. 27 (Supplement 1) (pp 1014), 2019. Date of Publication: 2019., Ghazvini Zadegan F., Seyed Frotan F., Ghaedi K., Nasr Esfahani M.

553. Irisin a candidate biomarker for distinguishing thyroid oncocytic variant carcinomas., Turkish Journal of Biochemistry. Conference: 2nd In-Vitro Diagnostic Symposium, IVD 2017. Turkey. 42 (Supplement 2) (pp 79), 2017. Date of Publication: December 2017., Aydin S., Kuloglu T., Artas G., Kocdor M.A., Sahin I., Yardim M., Ozercan I.H.

554. Skeletal muscle-specific PGC-1a overexpression prevents atherosclerosis in apolipoprotein e-knockout mice., Atherosclerosis Supplements. Conference: 18th International Symposium on Atherosclerosis, ISA 2018. Canada. 32 (pp 17-18), 2018. Date of Publication: June 2018., Pazgan-Simon M., Kukla M., Simon K., Lekstan A. Gabrek E., Zuwala-Jagiello J.

555. Steady low intensity physical activity and healthy dietary habits are differently affect breast cancer progression, Breast. Conference: 16th St. Gallen International Breast Cancer Conference. Austria. 44 (Supplement 1) (pp S34-S35), 2019. Date of Publication: April 1 2018., Kim M.K., Ahn S.K., Kim J.-H.

556. Non-small cell lung cancer cells induce the expression of adipokines in brown adipose tissue in the context of cancer cachexia., Pneumologie. Conference: 60. Kongress der Deutschen Gesellschaft fur Pneumologie und Beatmungsmedizin e. V.. Germany. 73 (SUPPPL. 1) (no pagination), 2019. Date of Publication: March 2019., Frille A., Kuhn H., Ebert T., Seyfarth H.J., Wirtz H.

557. Nutrition Education and Quality of Life in Cancer Cachexia NCT05262192 https://clinicaltrials.gov/show/NCT05262192, 2022 | added to CENTRAL: 31 March 2022 | 2022 Issue 03

558. Physical activity and Alzheimer's disease-2: clinical trial protocol JL Etnier, L Wideman, WB Karper, JD Labban, CN Wahlheim, TM Williams, YP Mobley, AB Slutsky, KS Park, NT Berry Journal of prevention of alzheimer's disease, 2019, 6, S50- | added to CENTRAL: 31 July 2020 | 2020 Issue 07

559. The effect of date seed (Phoenix dactylifera) supplementation on inflammation, oxidative stress biomarkers, and performance in active people: a blinded randomized controlled trial protocol E Moslemi, P Dehghan, M Khani Contemporary clinical trials communications, 2022, 28 | added to CENTRAL: 31 July 2022 | 2022 Issue 07

560. Slimming Myokines, Cancers,Nutritional and Psychology Support NCT05278078 https://clinicaltrials.gov/show/NCT05278078, 2022 | added to CENTRAL: 31 March 2022 | 2022 Issue 03

561. The impact of high-intensity interval exercise training on NK-cell function and circulating myokines for breast cancer prevention among women at high risk for breast cancer AM Coletta, NH Agha, FL Baker, GM Niemiro, PL Mylabathula, AM Brewster, TB Bevers, E Fuentes-Mattei, K Basen-Engquist, SC Gilchrist, RJ Simpson Breast cancer research and treatment, 2021, 187(2), 407-416 | added to CENTRAL: 31 March 2021 | 2021 Issue 3
